# Ordering Bent and Straight Dicarboxylate Linkers in an fcu Zirconium Metal–Organic Framework

**DOI:** 10.1021/jacs.5c05854

**Published:** 2025-07-23

**Authors:** Grace S. G. Farmer, Daniel J. Cheney, Khai-Nghi Truong, Nusik Gedikoglu, Bhupendra P. Mali, Datta Markad, Dmytro Antypov, Frédéric Blanc, Alexandros P. Katsoulidis, Matthew J. Rosseinsky

**Affiliations:** † Department of Chemistry, 4591 University of Liverpool, Crown Street, L69 7ZD Liverpool, U.K.; ‡ Leverhulme Research Centre for Functional Materials Design, Materials Innovation Factory, University of Liverpool, L7 3NY Liverpool, U.K.; § Rigaku Europe SE, Hugenottenallee 167, 63263 Neu-Isenburg, Germany; ∥ Stephenson Institute for Renewable Energy, University of Liverpool, L69 7ZF Liverpool, U.K.

## Abstract

The ordering of multiple organic linkers with different sizes and geometries on metal–organic frameworks (MOFs) offers structures with advanced complexity and functionality for enhanced properties. The selection of the components and the number of synthesis steps are commonly rationalized to match existing structural motifs in order to increase the probability of producing the designed new frameworks. Alternatively, combining multiple components, without a preconceived structural model, allows for the exploration of unprecedented structural characteristics. However, this approach requires a large number of experiments to identify both the composition and conditions for a successful synthesis. Here, we report a new Zr-MOF containing ordered straight and bent dicarboxylate linkers prepared by a one-pot synthesis. The discovery was made by high-throughput exploration of the chemical space composed of two linkers, the source of Zr, and the modulator. The linkers and the Zr_6_ cluster are ordered on an **fcu** framework that is tetragonally distorted from its typical cubic symmetry. The arrangement of linkers with different sizes and geometries affords cages and windows with shapes that have not been reported previously, despite the plethora of known **fcu**-based MOFs. The new MOF exhibits chromatographic separation of the *n*-hexane/benzene/cyclohexane mixture and demonstrates reversible unbinding and rebinding of the bent linkers upon the addition and removal of protic solvents. The unusual structural properties of the new MOF arise from ordering linkers in a non-predetermined manner following high-throughput synthesis exploration.

## Graphical abstract

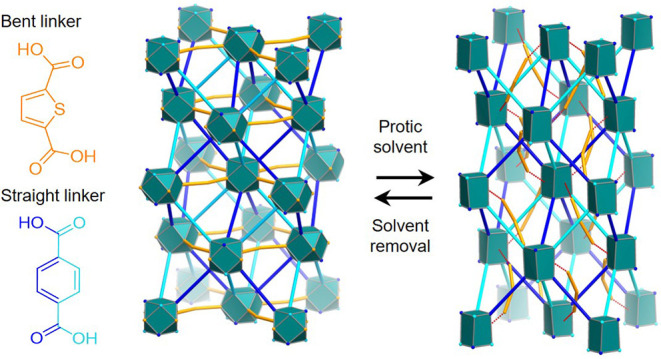

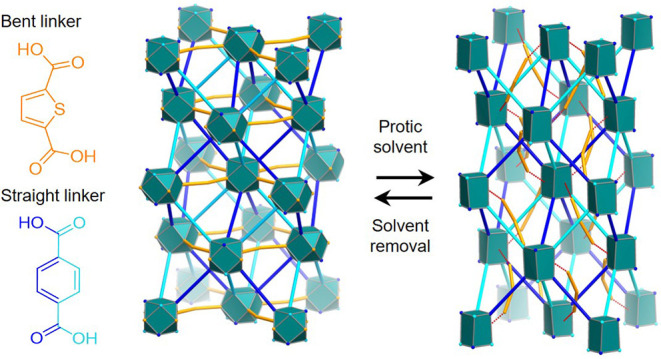

## Introduction

Metal–organic frameworks (MOFs) consist of metal-based nodes bridged by organic linkers to form crystalline and porous frameworks.^1^ Zirconium MOFs (Zr-MOFs) are well-known for their high structural and thermal stability, with a large variety of reported structures and applications.^2,3^ UiO-66 is the most well-known Zr-MOF, comprising [Zr_6_(μ_3_-O)_4_(μ_3_–OH)_4_]^12+^ clusters and 1,4-benzene dicarboxylate (BDC) linkers.^4,5^ Each Zr_6_ cluster is surrounded by 12 BDC linkers connected to further Zr_6_ clusters, forming the high-symmetry, 12-connected (12-c) framework with the **fcu** topology.^6^ The use of ditopic linkers with differing geometry and lower symmetry compared to BDC provides access to a range of frameworks with different symmetries and connectivities. The available ditopic linkers typically exhibit straight, zigzag, and bent geometries.^7,8^ Zigzag linkers, such as fumaric acid and 2,6-naphthalenedicarboxylic acid (NDC), can form the **fcu** framework topology,^9,10^ as well as 8-c frameworks with the **bcu** topology.^11^ However, the framework connectivity for bent linkers is considerably more complex. Several **fcu** Zr-MOFs, such as Zr-UiO-66-PZDC MOF,^12^ and BUT-10,^13^ with bent linkers with an angle of 160° between carboxylates in the framework have been reported. Within these structures, the Zr_6_ clusters rotate around multiple axes to accommodate the bent linker shape in three dimensions. However, bent linkers with a more acute angle between carboxylates result in further rotation of the clusters, which reduces the favorability of a 12-c framework.^14^ 2,5-Thiophenedicarboxylic acid (TDC) is an extensively explored bent linker in Zr-MOF chemistry. The Kaskel group has discovered four polymorphs of single-linker Zr-TDC MOFs, highlighting the different ways in which TDC can be arranged around the Zr_6_ cluster. In contrast, other known bent linkers, such as isophthalic acid^15^ and 2,5-furandicarboxylic acid,^16^ have not demonstrated a comparable number of reported structures or the same level of structural diversity as TDC in Zr-MOF chemistry. Each Zr-TDC polymorph displays a distinct topology, with three of them being 8-connected frameworks (DUT-67, -68, and -126) with **reo**, **bon**, and **hbr** topologies, respectively, while one is a 10-c Zr-MOF (DUT-69) with a **bct** topology.^17,18^ An **fcu** zirconium framework with TDC linkers has not been reported, and this can be understood as a mismatch between linker geometry and framework topology.^8^

Multicomponent MOFs^19,20^ display ordered arrangements of multiple linkers and differ from multivariate MOFs,^21^ which have the linkers disordered over the framework.^22^ The ordering of multiple linkers can produce new cages and windows that are not achievable with a single linker, resulting in enhanced performance in applications such as adsorption and separation.^23,24^ Multicomponent Zr-MOFs are of interest due to their high stability and structural diversity, but they are predominantly synthesized by multistep procedures.^25,26^ Therefore, their structures are restricted to those that can be derived by functionalizing the structure formed in the first synthetic step.^27^ The direct combination of multiple linkers in one synthetic step can deliver new structural features due to the assembly of linkers in a non-predetermined manner; however, this approach requires screening of many possible combinations of the composition parameters. A high-throughput synthesis (HTS) workflow facilitates the systematic exploration of large chemical spaces defined by variables such as the ratio of two or more linkers, the ratio of the linkers to the metal source, and the amount of modulator. HTS implementation in materials synthesis is growing, with notable examples in the porous materials community for zeolites,^28^ organic porous cages,^29^ covalent organic frameworks (COFs),^30^ and MOFs.^31^ Our HTS workflow, involving robotic liquid dispensing, was previously utilized to discover the first multicomponent Zr-MOF with equal amounts of straight (BDC) and zigzag (fumarate) linkers via a one-step synthesis.^32^ This framework is described by the **fcu** topology and rhombohedral symmetry due to the ordered decoration of the net by the two linkers of different lengths and geometries.

In this work, we implement an HTS workflow to explore efficiently the combination of bent TDC and straight BDC ditopic linkers within Zr-MOF chemistry. The goal is to target the assembly of Zr-MOFs with distinct structural features arising from component linkers that individually afford Zr-MOFs with different topologies. We present a new **fcu** Zr-MOF containing both BDC and TDC in tetragonal symmetry, discovered in a facile one-step synthesis using the ZrOCl_2_.8H_2_O/BDC/TDC/formic acid (FA)/dimethylformamide (DMF) system. TDC forms part of a 12-connected framework in this chemistry for the first time. The effect of geometry mismatch between the bent TDC geometry and the **fcu** topology is illustrated by the behavior of the framework after guest exchange with methanol, wherein the TDC linker reversibly disconnects from the framework in a structural transition. The distinct cage geometry of this ordered two-linker **fcu** structure enables separation of small molecules in gas chromatography.

## Results and Discussion

The combination of BDC and TDC was explored with formic acid (FA) as the modulator and DMF as the solvent. This ZrOCl_2_·8H_2_O/BDC/TDC/FA/DMF system was selected from the available literature data for the synthesis of existing single-linker MOFs. UiO-66 can be formed under many conditions using different solvents, modulators, reaction times, and reaction temperatures.^33,34^ DUT-67, the most explored TDC Zr-MOF, has been reported under conditions that overlap with those used for UiO-66.^35,36^ The solvent, DMF, and modulator, FA, successfully synthesized both MOFs and were chosen for the two-linker exploration. The explored chemical space spans a broad range of compositions, covered by a set of 60 points. Five molar ratios of the two linkers were explored, corresponding to BDC:TDC = 0.25:0.75, 0.33:0.67, 0.50:0.50, 0.67:0.33, and 0.75:0.25. Four molar ratios of ZrOCl_2_·8H_2_O to linker, Zr:(BDC + TDC), were investigated: 0.67:0.33, 0.50:0.50, 0.40:0.60, and 0.33:0.67. Three molar ratios of formic acid to ZrOCl_2_·8H_2_O (FA:Zr) were explored: 120, 310, and 500 (Figure 1a). The amount of modulator is of particular importance for exploratory Zr-MOF synthesis, as varying modulator amounts can result in UiO-66 formation with different defect densities^37^ or induce the formation of different phases, as reported for the TDC Zr-MOF polymorphs.^17^ Finally, the total liquid amount in the synthesis vial was fixed to 10 mL, and the reaction time and temperature were fixed to 48 h and 120 °C, respectively.

**Figure 1:**
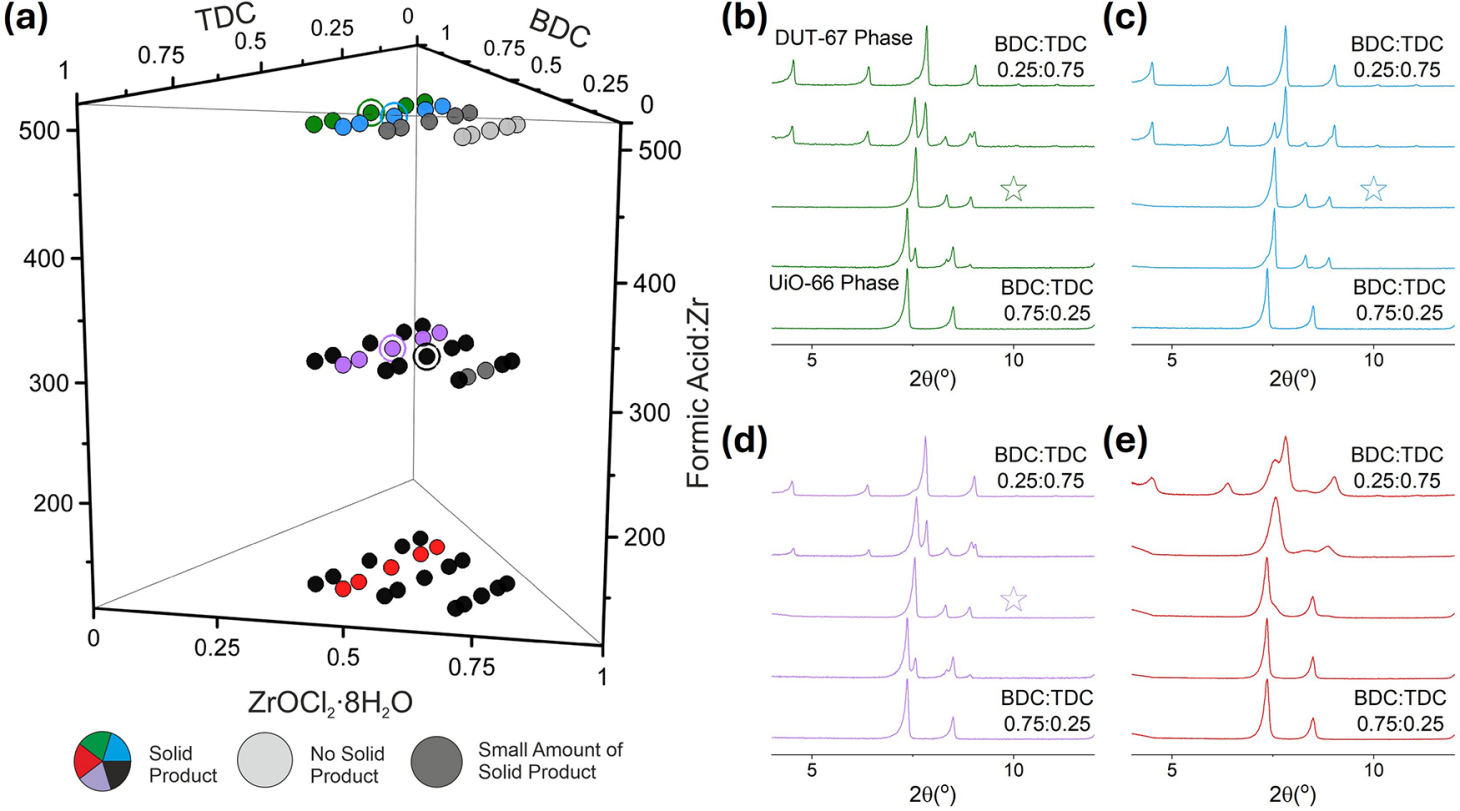
(a) 3D plot depicting the chemical space explored for the first batch of 60 reactions in the ZrOCl_2_·8H_2_O/BDC/TDC/FA/DMF system. The colored points (green, blue, black, purple, and red) indicate that sufficient solids are produced for their PXRD patterns to be collected (Cu K_α1_, λ = 1.5406 Å). The dark gray points yielded only a small amount of the solid product; thus, no PXRD measurements were performed. Light gray points indicate that no solid products are produced. The reaction compositions that produced the pure new phase are circled. (b–e) Sets of low-angle PXRD patterns from the points corresponding to the colors used in (a). The remainder of the patterns is provided in Figure S1. The patterns corresponding to the single-linker phases (DUT-67 and UiO-66) are noted in (b). Stars in (b–d) indicate patterns of pure new phase produced from the green, blue, and purple circled points, respectively, in (a). The PXRD pattern corresponding to the black circled point is provided in Figure S1c.

Visual inspection of the vials after the reaction showed that 55 out of the 60 batch samples had produced solid products, and the remaining five reaction mixtures produced clear solutions. Of the 55 samples with solid products, 48 produced enough solid for powder X-ray diffraction (PXRD) measurements. PXRD measurements showed that the majority of these yielded crystalline materials (Figures 1b–e and S1). The set of five PXRD patterns in Figure 1b is prepared from reaction mixtures that have a Zr:(BDC + TDC) ratio of 0.33:0.67. The remaining three sets (Figure 1c–e) are prepared from reaction mixtures that have the same Zr:(BDC + TDC) ratio of 0.40:0.60. Each set has a different FA:Zr ratio: 500 (Figure 1b,c), 310 (Figure 1d), and 120 (Figure 1e). Within each set, the linker ratio varies from BDC:TDC = 0.25:0.75 to BDC:TDC = 0.75:0.25. The formation of the known single-linker MOF phases UiO-66 and DUT-67 is observed in each set at high and low BDC:TDC ratios, respectively, and the appearance of a new phase is evident at intermediate ratios. As shown in Figure 1b, a cubic phase is present at BDC:TDC = 0.75:0.25, which corresponds to the UiO-66 structure. As BDC:TDC decreases to 0.67:0.33, a phase with low-intensity peaks emerges alongside the UiO-66-like phase, and at BDC:TDC = 0.50:0.50, the unknown phase appears as the only crystalline phase in the sample. These new peaks do not correspond to any of the known phases derived from the components of this system, indicating the discovery of a new phase. A further increase in the amount of TDC results in the appearance of low intensity peaks in the BDC:TDC = 0.33:0.67 pattern, which are identified as arising from a phase with the DUT-67 structure. At BDC:TDC = 0.25:0.75, the new phase disappears, and only the DUT-67 phase is present. In the panels of Figure 1c,d, the pure new phase is also observed at the two points where BDC:TDC = 0.50:0.50. Comparing the relative intensities of the peaks of the new phase to those of DUT-67 and UiO-66, with the same BDC:TDC ratio for these two sets, it is observed that the phase composition of the solid product differs depending on the Zr:(BDC + TDC) and FA:Zr ratios. For example, ratio BDC:TDC = 0.33:0.67 in Figure 1c shows more intense peaks of DUT-67 compared to the new phase, whereas in Figure 1d and for the same BDC:TDC ratio, the peaks for the new phase are more intense compared to those for DUT-67. In the set with the lowest FA:Zr = 120 ratio, the new phase is observed at BDC:TDC = 0.33:0.67, but the peaks are broad, and it is difficult to conclude about the phase purity of this sample. All of the samples produced at a FA:Zr ratio of 120 display relatively broad peaks compared to the higher ratios, which demonstrates the effect of the modulator amount in the present system to produce crystalline materials with sharp peaks that help distinguish those samples that correspond to phase-pure products.

The new phase appears broadly across the chemical space in a mixture with phases of the known single-linker MOF structures in 23 out of the 60 compositions explored and as a pure phase product in 4 out of 60 compositions. In addition to the three compositions delivering the pure phase shown in Figure 1b–d, the new phase was also identified as pure at BDC:TDC = 0.50:0.50, Zr:(BDC + TDC) = 0.67:0.33, and FA:Zr = 310 (Figure S1c).

A second iteration of 42 reactions in the same system was performed to understand the limits of the region of chemical space that produces the new phase pure material (Figure 2a). The composition points were selected to cover the space around those that yielded the new phase pure in the first batch, excluding points that yielded the known phases or lower-crystallinity products (Tables S3 and S4). Solid products were produced for all 42 reaction mixtures of the second batch, with 37 of 42 points identified as the pure target phase via PXRD (Figure S2). The four points replicated from batch 1 provided patterns identical to those of the prior reactions (Figure S3). The remaining five points contain low intensity peaks attributed to the DUT-67-like phase in addition to the new phase. ^1^H nuclear magnetic resonance (NMR) analysis on the digested solids of phase-pure samples demonstrates the presence of both linkers, with variance in their ratio (BDC:TDC from 0.64:0.36 to 0.53:0.47) depending on the reaction mixture compositions (Table S5, Figures S4 and S16). The HTS exploration demonstrated that a new phase can be formed over a large chemical space. All of the hits from the first batch were at equimolar BDC:TDC ratios (0.50:0.50); however, the exploration within the narrower space of batch 2 demonstrated that pure phase is produced at reaction mixture linker ratios varying from BDC:TDC = 0.43:0.57 to BDC:TDC = 0.57:0.43. Interestingly, the new phase is synthesized over a broader range of reaction compositions compared to the previously reported two-linker ordered MOF, Zr_6_(BDC)_3_(Fum)_3_, which is formed over a restricted and narrow region of the ZrOCl_2_·8H_2_O/BDC/fumarate/DMF/FA system.^32^

**Figure 2:**
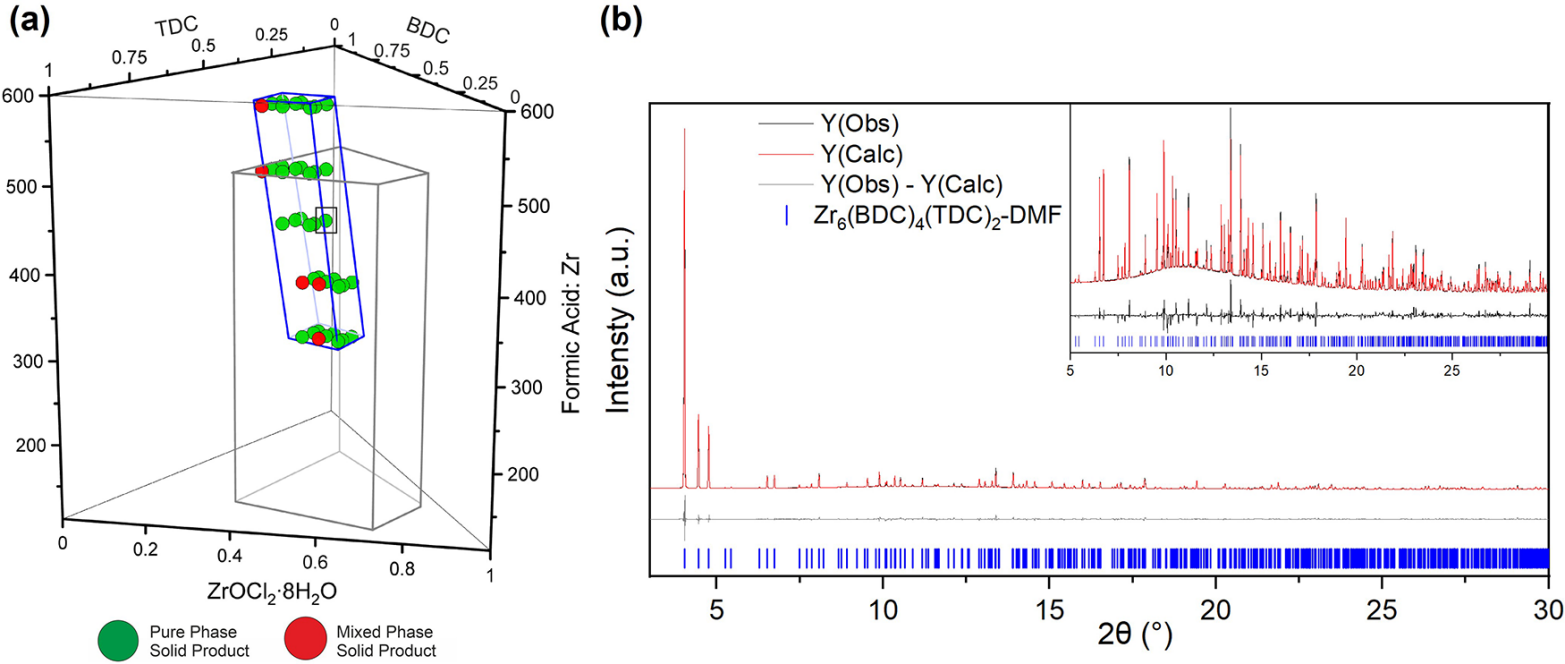
(a) 3D plot to represent the chemical space explored in the second batch by 42 reactions. The area bound by the gray trapezoidal prism represents the chemical space explored in the first batch of reactions, and the area outlined in blue represents the space explored in the second batch. The green points represent the reactions that yielded the pure new phase, and the red points represent those reactions that yielded the new phase alongside a small impurity of the DUT-67 phase. The PXRD patterns of the second batch are provided in Figure S2. (b) Rietveld refinement of Zr_6_(BDC)_4_(TDC)_2_-DMF from the PXRD pattern of the solid obtained from the point highlighted by a square in (a) with the following composition: BDC:TDC = 0.57:0.43, Zr:(BDC+TDC) = 0.67:0.33, and FA:Zr = 440.

## Structural Description

The new phase, Zr_6_(BDC)_4_(TDC)_2_-DMF, crystallizes in tetragonal space group *I*4_1_/*acd* with unit cell parameters *a* = 19.88173(5) Å and *c* = 42.49860(13) Å and with a unit cell volume *V* = 16798.99(10) Å^3^ (Figure 3a). The size of the crystallites ranges from 500 to 600 nm (Figure S5), which is too small for single-crystal X-ray diffraction measurements. Therefore, the crystal structure was solved using synchrotron PXRD data. Simulated annealing was implemented to construct the structural model (Figure S6), and the refinement was carried out with the Rietveld method (see Structure Determination in the Supporting Information) on the data collected for the as-made sample with formula Zr_6_O_4_(OH)_4_(BDC)_3.56_(TDC)_1.96_(HCOO)_0.96_·6.99DMF (Figure 2b). This sample, synthesized from the reaction mixture with BDC:TDC = 0.57:0.43, Zr:(BDC + TDC) = 0.67:0.33, and FA:Zr = 440, exhibits high crystallinity, and its formula close approximates the theoretical formula of the framework Zr_6_O_4_(OH)_4_(BDC)_4_(TDC)_2_. The center of the [Zr_6_O_4_(OH)_4_]^12+^ clusters corresponds to Wyckoff positions 8*a* of the *I*4_1_/*acd* space group. The clusters adopt the **fcu** net with 12 neighbors, which is most clearly described in terms of their distribution over successive 00l planes (Figure 3a). Each cluster has four adjacent coplanar clusters at a distance of 14.06 Å and to eight other clusters—four located on the plane above and four on the plane below, at 14.55 Å (Figure S6). These distances are similar to the cluster–cluster distances in DUT-67 (13.83 Å) and UiO-66 (14.66 Å), suggesting the location of four TDC and eight BDC linkers around each [Zr_6_O_4_(OH)_4_]^12+^ (Figure 3b). The linkers were introduced at these positions and refined to sensible geometries, with their occupancies fixed to values obtained from ^1^H NMR analysis of the digested sample, in combination with thermogravimetric analysis (TGA) results (see the Chemical Formula of Bulk Samples in the Supporting Information). The structural model of Zr_6_(BDC)_4_(TDC)_2_-DMF is described as an **fcu** net featuring an ordered arrangement of eight straight (BDC) and four bent (TDC) linkers (Figure 3a). The reduced tetragonal *I*4_1_/*acd* space group symmetry of Zr_6_(BDC)_4_(TDC)_2_-DMF compared to cubic *Fm*3̅*m* of single-linker UiO-66 arises from the ordering of linkers that differ in size and geometry. The difference in linker length induces tetragonal distortion, resulting in the doubling of the *c*-axis (Figure 4a) to accommodate the bent TDC around Zr_6_ clusters in two orientations: one with the sulfur of the thiophene rings pointing clockwise and the other pointing anticlockwise (Figure 4b–e). Thus, through the 8 + 4 ordering with BDC, TDC gains access to the high-symmetry **fcu** net, which is inaccessible in single-linker TDC Zr-MOF analogues, like DUT-67. The other known **fcu** Zr-MOF with 8 + 4 linkers, ZRN-XB, was accessed by the postsynthetic linker installation of X-BDC (X = OH, −NO_2_, and −NH_2_) into the 8-c ZRN-**bcu** framework described by eight NDC zigzag linkers. The addition of the straight BDC linker in that case does not reduce further the *I*4/*mmm* symmetry of the starting 8-c framework because the point group symmetry of BDC (*mmm*) matches the symmetry of the site where the BDC is inserted.^38^ Zr_6_(BDC)_4_(TDC)_2_-DMF is the first reported Zr-MOF with the *I*4_1_/*acd* space group and the first **fcu** Zr-MOF containing ordered straight and bent dicarboxylate linkers.

**Figure 3:**
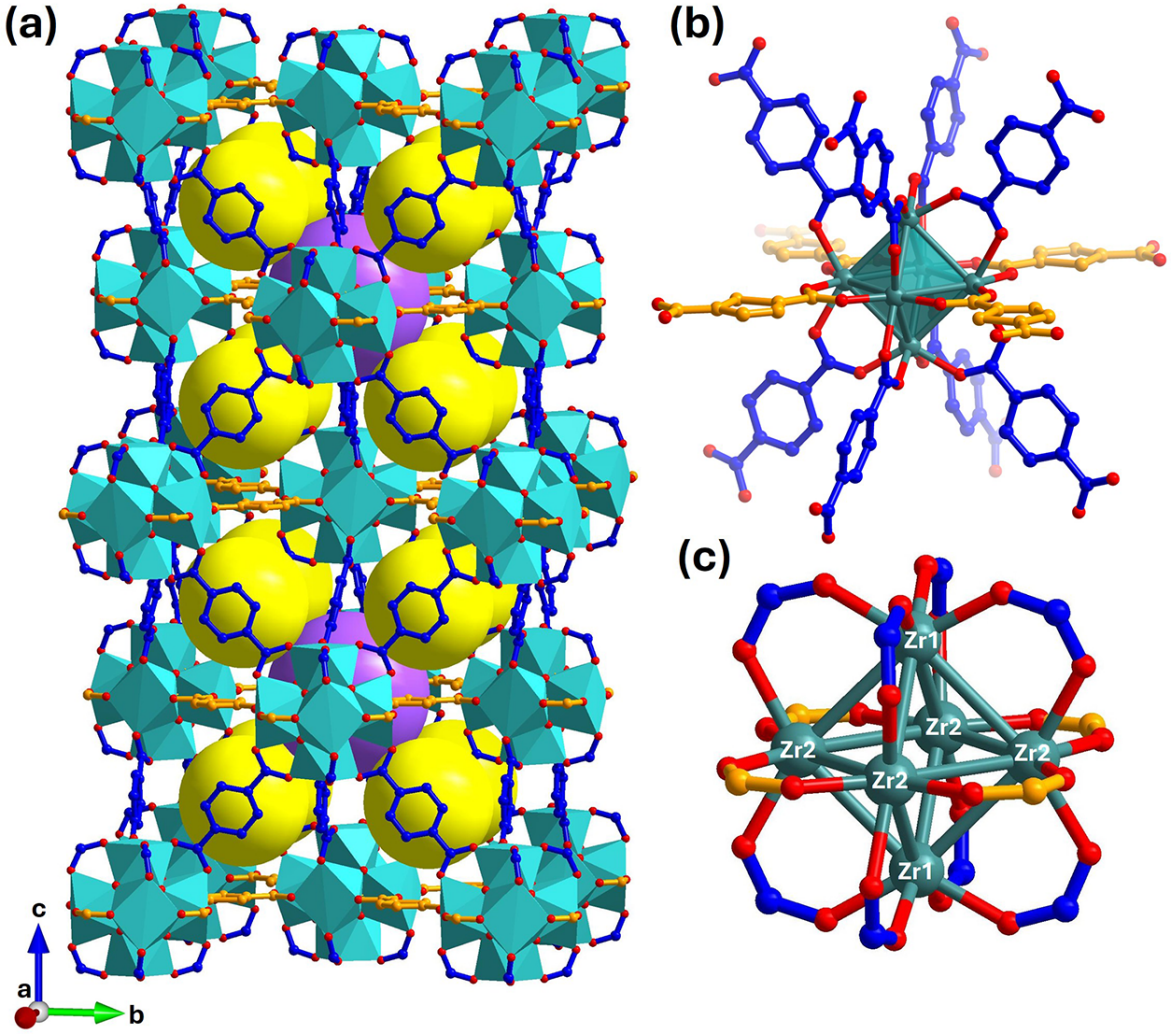
(a) Tetragonal unit cell of the Zr_6_(BDC)_4_(TDC)_2_-DMF crystal structure. [Zr_6_O_4_(OH)_4_]^12+^ clusters are represented by teal polyhedra, with Zr atoms coordinated by O atoms in red. BDC linkers are shown in blue, and TDC linkers are shown in orange. Yellow and purple spheres represent the guest-accessible tetrahedral and octahedral cages, respectively. (b) Zr_6_ octahedron surrounded by eight BDC and four TDC linkers. (c) Zr_6_ octahedron coordination by the carboxylate groups of the BDC and TDC linkers. The crystallographically distinct Zr atoms are labeled as Zr1 and Zr2. H atoms and solvent molecules are omitted for clarity from all depictions, as are the hydroxides and oxides on the [Zr_6_O_4_(OH)_4_]^12+^ cluster.

**Figure 4:**
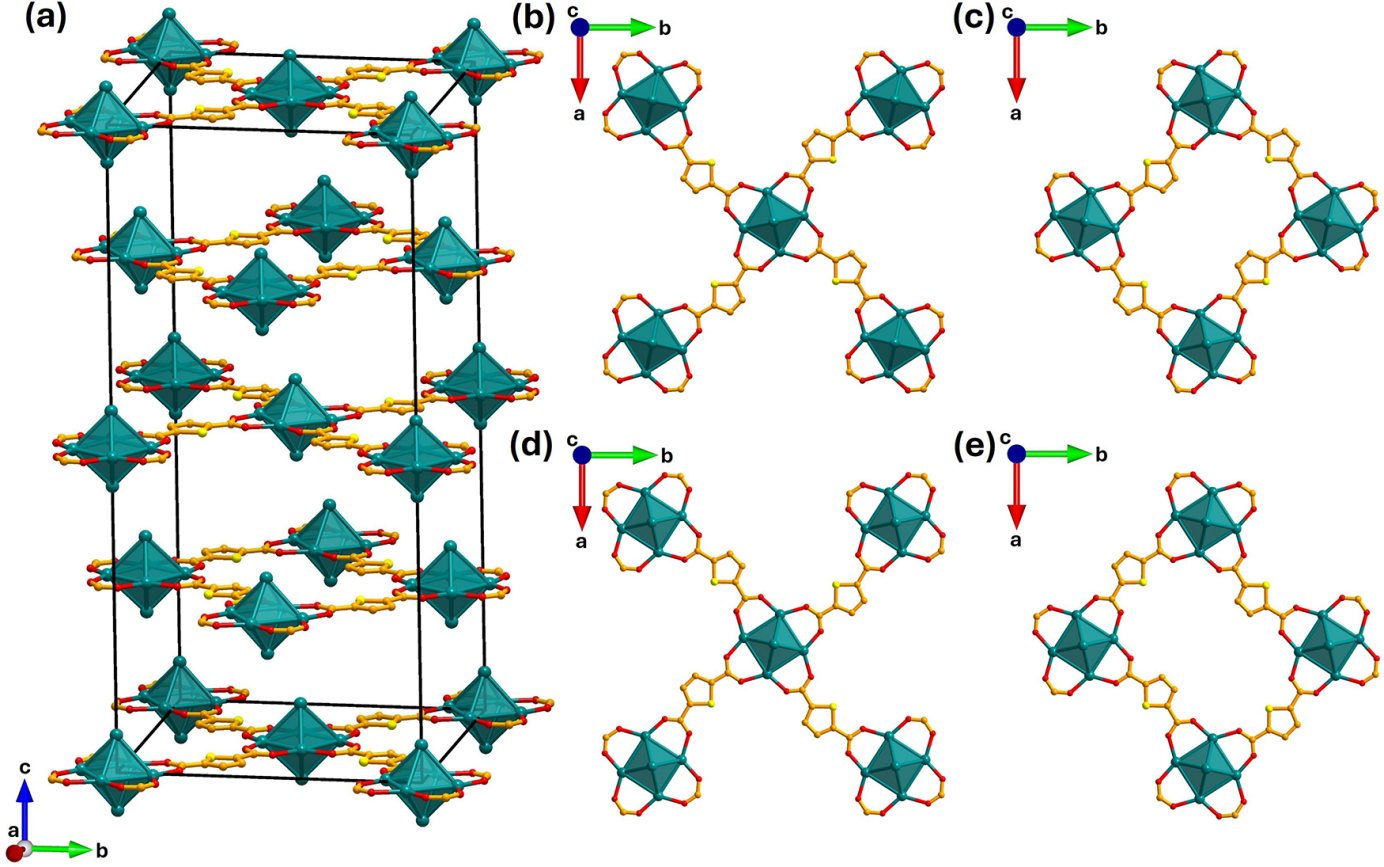
(a) Stacking of layers of Zr_6_ octahedra and TDC in the unit cell of Zr_6_(BDC)_4_(TDC)_2_-DMF corresponding to the doubling of the *c*-axis compared to the UiO-66 crystal structure (Figure S13). (b–e) Four Zr_6_(TDC)_2_ layers stacked from top to bottom in the unit cell of Zr_6_(BDC)_4_(TDC)_2_-DMF. Comparison of panel (b) with (d) and panel (c) with (e) revealed that TDC adopts two orientations, with the sulfur atoms of the thiophene ring pointing in opposite directions, connecting Zr_6_ octahedra that rotate in opposite senses. The sulfur atoms of the TDC linker are highlighted in yellow to emphasize the directionality of the thiophene ring. Zr is shown in teal, and O is shown in red; the C atoms of the TDC linkers are shown in orange, and the S atoms of TDC are shown in yellow. The hydroxides and oxides on the Zr_6_ cluster, the BDC linkers, H atoms, and solvent molecules are omitted for clarity.

The six Zr atoms of the [Zr_6_O_4_(OH)_4_]^12+^ cluster in Zr_6_(BDC)_4_(TDC)_2_-DMF form a slightly compressed octahedron, Zr_6_, along the *c*-axis resulting from the tetragonal distortion (Figure S12). There are two independent crystallographic Zr sites: Zr1 occupying the two axial sites and Zr2 occupying the four planar sites (Figure 3c). The Zr1–Zr2 edge is occupied by the carboxylates of BDC, and the Zr2–Zr2 edge is occupied by the carboxylates of TDC.

Each TDC linker coordinates solely to Zr2 and connects two Zr_6_ clusters, which are rotated in opposite senses by 12° about the *c*-axis to accommodate the bent TDC geometry (Figure 4b–e). The assembly of TDC linkers and Zr_6_ clusters forms Zr_6_(TDC)_2_ layers parallel to the *ab* plane, composed of rectangular rings consisting of four bent TDC and four Zr_6_ clusters (Figure 5a); these rings are elongated compared to the regular squares observed in UiO-66 (Figure S13). The long axes of the rectangles alternate in their orientation between edge-sharing rings within the plane. The structural motif with coplanar TDC linkers and Zr_6_ clusters, produced by rotation around only one axis, is not observed in DUT-67 or any other single-linker Zr-TDC MOFs. DUT-67 contains a Zr_6_(TDC)_2_ layer with two kinds of squares, where the S of TDC points above or below the layer (Figure S14). However, in the single-linker Zr-TDC MOF structures, the Zr_6_ clusters rotate around multiple axes, bringing the TDC linkers out of plane in order to accommodate their bent shape in three dimensions, yielding nets with connectivity lower than 12.

**Figure 5:**
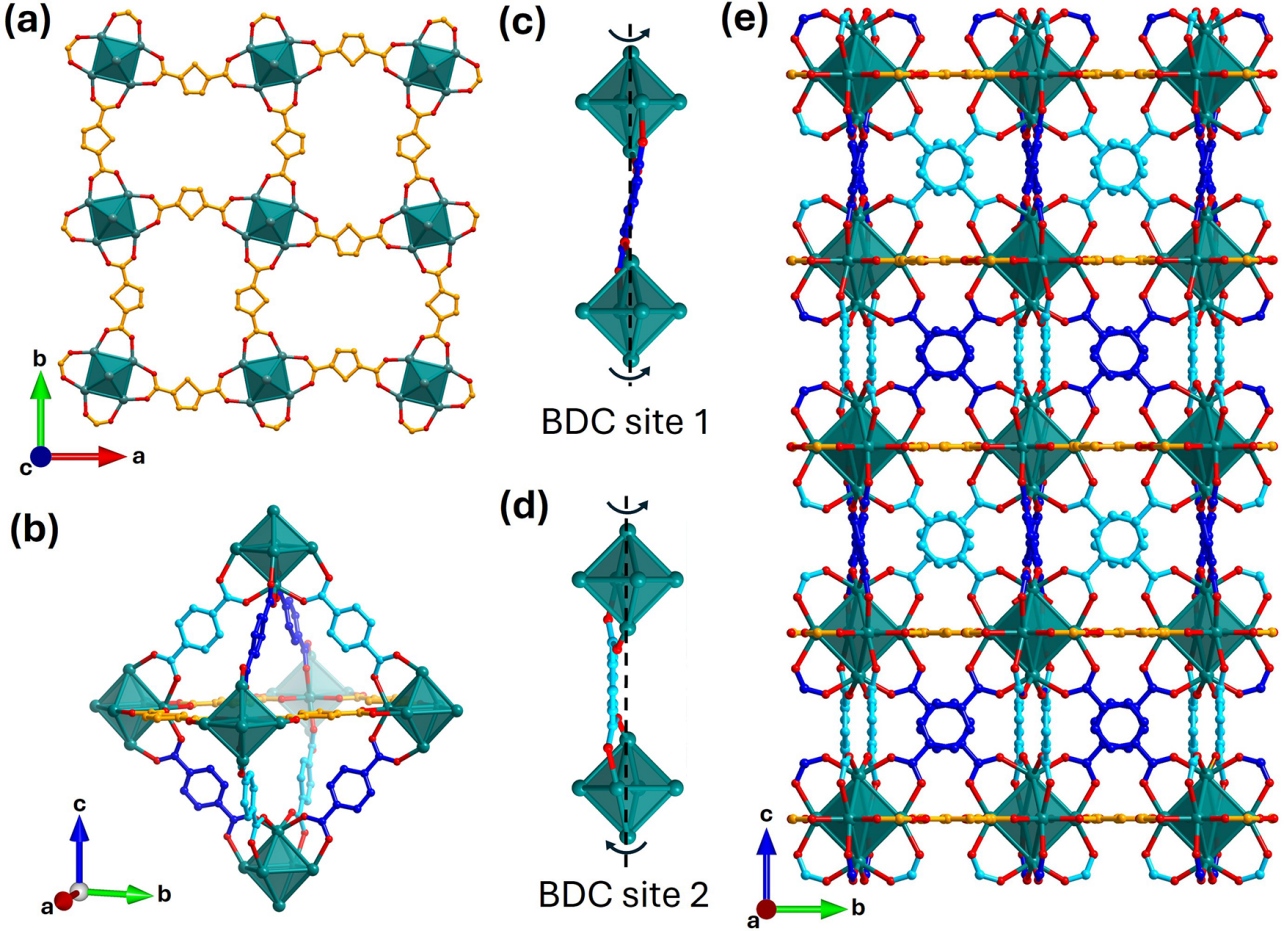
(a) Zr_6_(TDC)_2_ layer of rectangular rings formed by Zr_6_ octahedra and TDC. The rings are arranged in alternating orientations. (b) Octahedral cage of Zr_6_(BDC)_4_(TDC)_2_-DMF showing the clusters of a Zr_6_(TDC)_2_ layer connected to the above and below layers by two types of BDC linkers. (c) and (d) Zr_6_ octahedra connected by BDC linkers in two ways. (c) BDC on site 1 connects two clusters that rotate in the same sense and intersects the plane defined by Zr1 atoms (dashed line). The rotation of the clusters around the *c*-axis is highlighted with arrows. (d) BDC on site 2 connects two clusters that rotate in opposite senses and is displaced from the plane defined by the Zr1 atoms (dashed line). (e) Unit cell of Zr_6_(BDC)_4_(TDC)_2_-DMF highlighting the positions of BDC sites alternating down the unit cell, emphasized with a dashed line as shown in (c) and (d). Zr is shown in teal, O is shown in red, the C atoms of BDC site 1 are in blue, the C atoms of BDC site 2 are in cyan, and TDC is shown in orange. The hydroxides and oxides on the Zr_6_ cluster, H atoms, and solvent molecules are omitted for clarity.

Each Zr_6_(TDC)_2_ layer is connected to the neighboring layers of clusters by BDC linkers (Figure 5b). The pattern of alternating rotation of the clusters creates two crystallographic sites for BDC. Reflecting the effect of differing rotational pairs on the locations of Zr2 atoms in successive layers, BDC site 1 connects two Zr_6_ clusters that rotate in the same sense (Figure 5c) and thus is inclined such that it intersects the plane defined by their Zr1 sites. In contrast, BDC site 2 connects oppositely rotated clusters and therefore is displaced from this plane (Figure 5d). Both BDC sites are present in each Zr_6_(BDC)_2_ layer, which lie on the *ac* and *bc* planes, in alternate arrangement along the *c*-axis (Figure 5e). The BDC linkers in each layer are not coplanar, thus differing from the layers in UiO-66, which have a single BDC site connecting coplanar Zr atoms. The synergistic effect of the bent TDC and straight BDC linkers can accommodate the rotation of the Zr_6_ cluster about one axis and hence form the Zr_6_(BDC)_4_(TDC)_2_-DMF framework in the high-symmetry 12-c **fcu** net (Figure 5e).

Zr_6_(BDC)_4_(TDC)_2_-DMF was treated with methanol to replace DMF from the pores and produce a material, Zr_6_(BDC)_4_(TDC)_2_-MeOH, with a volatile solvent in the pores that could be activated at moderate temperatures. The completion of guest exchange was confirmed by ^1^H NMR (Figure S16), and the PXRD pattern of Zr_6_(BDC)_4_(TDC)_2_-MeOH presents the same diffraction peaks as Zr_6_(BDC)_4_(TDC)_2_-DMF at low angles; however, there are noticeable differences at higher angles (Figure 7a). Zr_6_(BDC)_4_(TDC)_2_-MeOH has the same space group as Zr_6_(BDC)_4_(TDC)_2_-DMF, *I*4_1_/*acd*, with similar unit cell parameters (*a* = 19.91546(12) Å, *c* = 42.6593(4) Å). The main structural difference between Zr_6_(BDC)_4_(TDC)_2_-MeOH and Zr_6_(BDC)_4_(TDC)_2_-DMF is the location and orientation of the bent TDC linker, which is no longer connected to the Zr_6_ clusters through Zr–O bonds (see Structure Determination in the Supporting Information). Instead, TDC is located in the space between the *ab* planes of Zr_6_ clusters (Figure 6a) with the carboxylate groups oriented toward Zr_6_ clusters that are also linked by the BDC of site 2 (Figure 6b). The Zr_6_ clusters and BDC linkers retain their sites and form an 8-c net with a **bcu** topology. The rotation of the Zr_6_ clusters has been reduced to 7°, from 12° in Zr_6_(BDC)_4_(TDC)_2_-DMF, resulting in BDC site 1 being less inclined and BDC site 2 being less displaced compared to Zr_6_(BDC)_4_(TDC)_2_-DMF. However, the displaced position of BDC site 2 creates space for the TDC linker to approach and form H-bonds with the cluster. The sites previously occupied by TDC carboxylates in the *ab* plane are filled by capping MeOH molecules. The TDC linker is stabilized by six hydrogen bonds: two with the μ_3_–OH groups of the Zr_6_ clusters and four with the capping MeOH molecules (Figure 6c). Its position is further stabilized by a favorable interaction between the TDC and BDC site 2 linker. The aromatic ring of the TDC is perpendicular to the aromatic ring of BDC site 2, with a distance of 4.718(3) Å between the thiophene ring and the benzene ring (Figure 6d).^39,40^ The structure of Zr_6_(BDC)_4_(TDC)_2_-MeOH demonstrates that methanol can disconnect and replace TDC from the Zr_6_ cluster, while TDC remains bound to the framework through hydrogen bonding (Figure 6e). This activity of methanol is similar to its role in postsynthetic linker exchange in UiO-66, where it facilitates the breaking of metal–linker Zr–O bonds and promotes the formation and stabilization of dangling linkers.^41^

**Figure 6:**
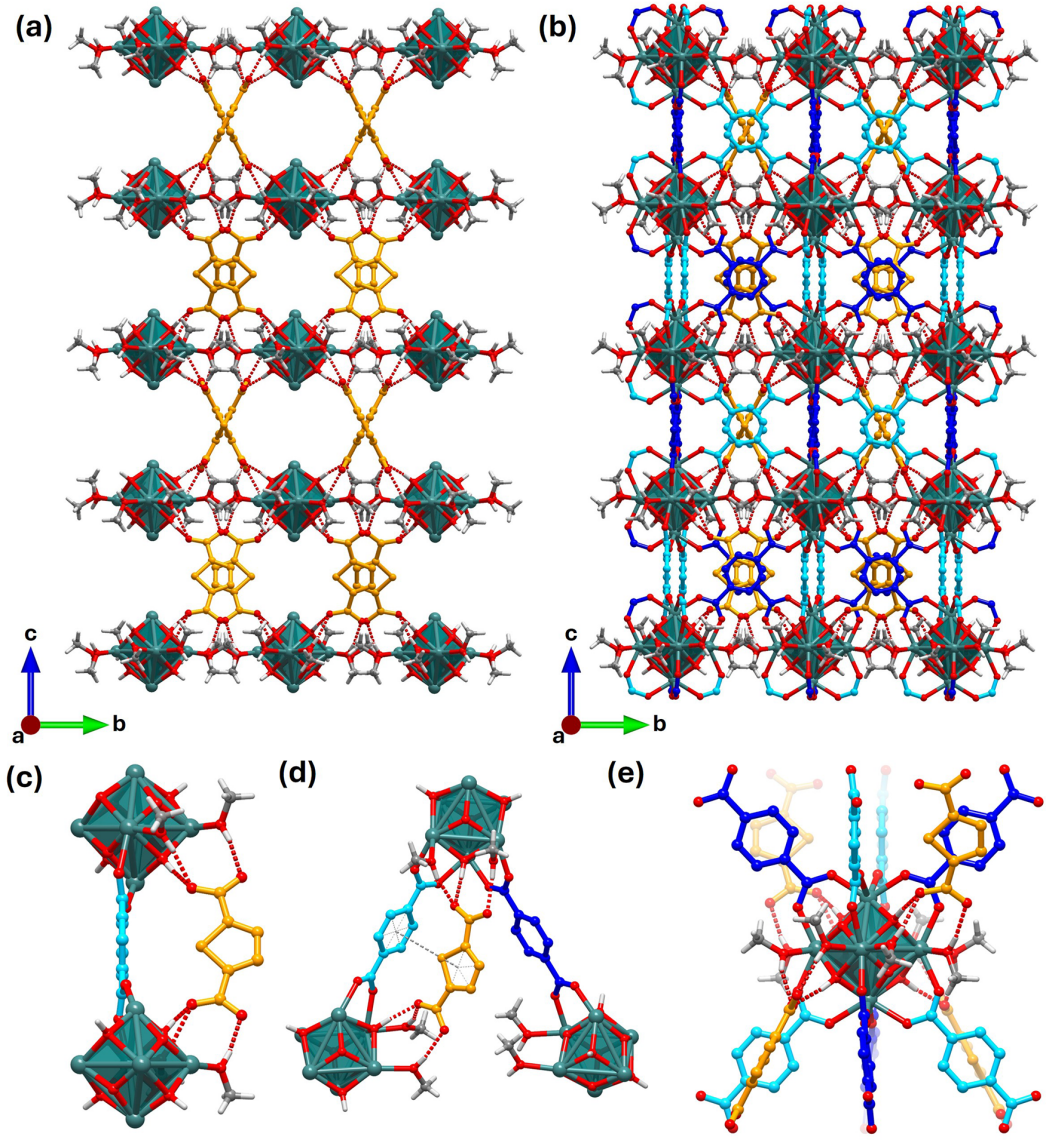
(a) In the Zr_6_(BDC)_4_(TDC)_2_-MeOH crystal structure, TDC is located between the layers of Zr_6_ octahedra (BDC is omitted). (b) Unit cell of the Zr_6_(BDC)_4_(TDC)_2_-MeOH crystal structure. (c) TDC linker is connected through hydrogen bonds to hydroxides and coordinated methanol molecules on two Zr_6_ clusters that are linked at BDC site 2. (d) Position of the TDC relative to BDC site 1 and BDC site 2. A gray dashed line is included to show the interaction between the TDC linker and the center of BDC site 2. This is further highlighted with light gray dashed lines within the aromatic rings. (e) Zr_6_ octahedron surrounded by eight BDC linkers and four TDC linkers in Zr_6_(BDC)_4_(TDC)_2_-MeOH. This can be compared to the analogous representation of Zr_6_(BDC)_4_(TDC)_2_-DMF (Figure 3b). For (c, d), only the methanol molecules involved in the TDC H-bonding are shown. For all depictions, the Zr_6_ nodes are shown as teal octahedra with the oxides and hydroxides included to show their interaction with the TDC. The methanol molecule coordinated to the Zr_6_ clusters is shown. The C atoms of BDC site 1 are in blue, the C atoms of BDC site 2 are in cyan, and the TDC linkers have orange C and S atoms. The oxygen atoms are shown in red. The methyl carbon of methanol is shown in gray, and all hydrogen atoms are shown in light gray. The red dotted lines represent hydrogen bonds between the TDC, the solvent coordinated to the cluster, and the cluster hydroxides. The solvent in the pores and the H atoms of both the linkers’ carbon atoms are omitted for clarity.

When Zr_6_(BDC)_4_(TDC)_2_-MeOH is exposed to air overnight, the methanol is exchanged for atmospheric water. This exchange produces Zr_6_(BDC)_4_(TDC)_2_-H_2_O and is confirmed by the disappearance of methanol signals in digestion ^1^H NMR over a 24 h time period (see Solvent exchange and activation in the Supporting Information, Table S10 and Figure S20). This is also supported by the disappearance of methanol signals in ^1^H magic angle spinning (MAS) NMR (Figure S22ii,iii) at 2.9 and 4.7 ppm and the emergence of a signal at 4.2 ppm (which is assigned to H_2_O bound to the Zr_6_ cluster). The PXRD pattern of Zr_6_(BDC)_4_(TDC)_2_-H_2_O is very similar to that of Zr_6_(BDC)_4_(TDC)_2_-MeOH (Figure 7a.ii and iii, respectively) and is indexed in the same space group, *I*4_1_/*acd*, with unit cell parameters *a* = 19.8623(1) Å and *c* = 42.7463(3) Å. The structure of Zr_6_(BDC)_4_(TDC)_2_-H_2_O was elucidated by continuous rotation three-dimensional electron diffraction (3D ED) measurements,^42,43^ which allowed the study of submicron-sized crystals; the results demonstrated that TDC occupies a similar position to that in Zr_6_(BDC)_4_(TDC)_2_-MeOH, with H_2_O molecules serving as capping ligands on the Zr_6_ clusters (Figure S15).

**Figure 7:**
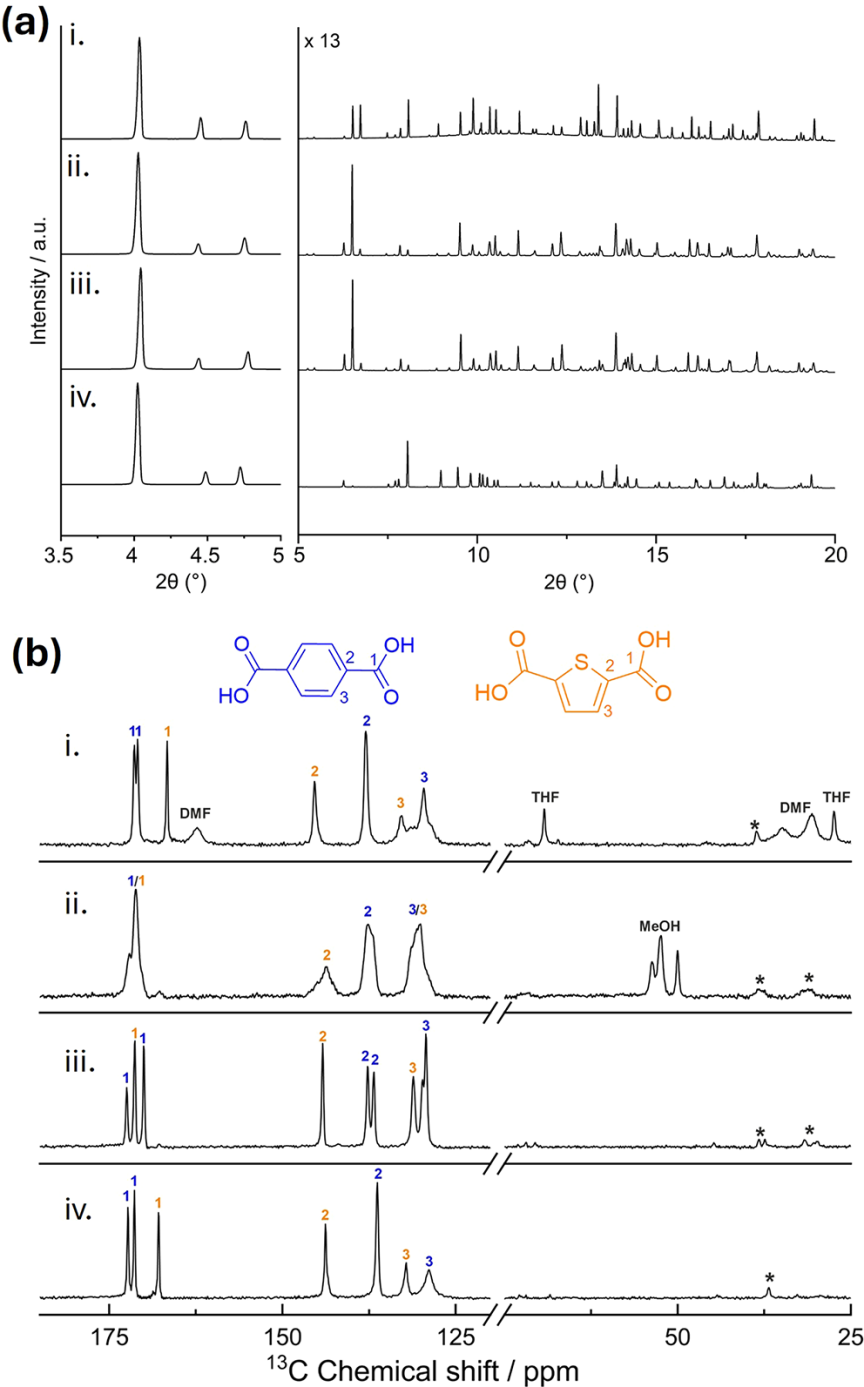
(a) Synchrotron PXRD patterns and (b) ^13^C CP MAS NMR spectra (shown for ranges 180–130 ppm and 100–25 ppm) of (i) Zr_6_(BDC)_4_(TDC)_2_-DMF, (ii) Zr_6_(BDC)_4_(TDC)_2_-MeOH, (iii) Zr_6_(BDC)_4_(TDC)_2_-H_2_O, and (iv) activated Zr_6_(BDC)_4_(TDC)_2_. NMR signal assignments are indicated by colored numbers correlating to the carbon environments of BDC (blue) and TDC (orange) linkers. In spectra ii of Zr_6_(BDC)_4_(TDC)_2_-MeOH, the broad signals at 171 and 130.5 ppm are attributed to carbon environments in both BDC and TDC and are labeled with two colored numbers. Signals attributed to solvents are indicated on the spectra, and spinning sidebands are marked with asterisks (*).

The observation of 8-c net Zr_6_(BDC)_4_ in Zr_6_(BDC)_4_(TDC)_2_-MeOH and Zr_6_(BDC)_4_(TDC)_2_-H_2_O structures prompts the investigation of any intermediate phase during the reaction synthesis of Zr_6_(BDC)_4_(TDC)_2_-DMF. A set of synthesis experiments at shorter reaction times (see Shorter reaction synthesis of Zr_6_(BDC)_4_(TDC)_2_-DMF in the Supporting Information) demonstrated that a small amount of UiO-66 is formed at the initial stage of the reaction but gradually disappears as Zr_6_(BDC)_4_(TDC)_2_-DMF forms and becomes the only phase present in the reaction mixture (Table S11 and Figure S21).

The structural transformation from Zr_6_(BDC)_4_(TDC)_2_-DMF to Zr_6_(BDC)_4_(TDC)_2_-MeOH and Zr_6_(BDC)_4_(TDC)_2_-H_2_O was corroborated by MAS NMR measurements. The ^13^C cross-polarization (CP) MAS spectra of Zr_6_(BDC)_4_(TDC)_2_-DMF are depicted in Figure 7b.i. Spectral assignment of the BDC and TDC signals was aided through ^1^H MAS NMR spectra (Figure S22i and Table S12) and two-dimensional (2D) ^1^H–^13^C HETeronuclear CORrelation (HETCOR) NMR spectra (Figure S23), with the chemical shifts being in good agreement with those previously reported for UiO-66 and DUT-67.^44,45^ There are also signals assigned to DMF and tetrahydrofuran (THF) (see Solvent exchange and activation in the Supporting Information). The ^13^C CP MAS spectrum of Zr_6_(BDC)_4_(TDC)_2_-MeOH (Figure 7b.ii) exhibits no signals arising from DMF, which confirms the removal of the DMF molecules from the pores. Three new signals appear between 50 and 55 ppm, which are associated with the methyl group of MeOH. This indicates that MeOH adopts three different environments in this structure: one as an unbound mobile guest in the pores at 50 ppm (as confirmed by the significantly increased intensity of this peak in the directly excited ^13^C MAS NMR spectrum versus the CP spectrum, Figure S24)^46^ and two as capping ligands on the Zr_6_ cluster at 52 and 54 ppm. In the HETCOR spectrum (Figure S25), these signals correlate with the aromatic ^1^H signals, indicating spatial proximity. Further correlations are observed between the carboxylate ^13^C signals and a ^1^H signal at 5.7 ppm (which likely corresponds to the OH protons of capping methanol), illustrating that methanol binds to the Zr_6_ cluster, in agreement with the refined structure of Zr_6_(BDC)_4_(TDC)_2_-MeOH. The peaks of BDC and TDC carbons are broad due to the presence of MeOH in the pores. In Zr_6_(BDC)_4_(TDC)_2_-H_2_O, the three MeOH ^13^C signals disappeared, confirming that this solvent has largely evaporated (Figure 7b.iii). The HETCOR spectrum (Figure S26) shows correlations between the linker ^13^C signals and a ^1^H signal at 5.4 ppm, which likely corresponds to H_2_O bound to the Zr_6_ cluster, confirming that water has replaced methanol as the capping ligand and is now in close proximity to the linkers. The lines are also significantly narrower than those for Zr_6_(BDC)_4_(TDC)_2_-MeOH, and signals arising from BDC and TDC can be distinguished in the HETCOR spectrum. The carboxylate signal of TDC has been shifted to 171 ppm compared to 167 ppm for Zr_6_(BDC)_4_(TDC)_2_-DMF. This shift indicates an environment change for the TDC carboxylate group in both Zr_6_(BDC)_4_(TDC)_2_-MeOH and Zr_6_(BDC)_4_(TDC)_2_-H_2_O, which is consistent with the change of the TDC position in the refined crystal structures. In both Zr_6_(BDC)_4_(TDC)_2_-DMF and Zr_6_(BDC)_4_(TDC)_2_-H_2_O, there are two signals attributed to the BDC carboxylate carbons due to the two crystallographic sites for BDC within the framework, leading to slightly different metal–O_carboxylate_ bonding to the Zr_6_ cluster. There is one ^13^C MAS NMR signal for the quaternary aromatic carbons (at 138 ppm), representing both BDC sites for Zr_6_(BDC)_4_(TDC)_2_-DMF; however, in Zr_6_(BDC)_4_(TDC)_2_-H_2_O, these BDC sites are now clearly resolved into two signals. The differences between the BDC sites are increased because BDC site 2 is now interacting with the thiophene ring of the relocated TDC linker, whereas BDC site 1 is not (Figure 6d).

H_2_O was removed from Zr_6_(BDC)_4_(TDC)_2_-H_2_O through activation at 150 °C and 10^–3^ mbar for 16 h, as confirmed by the absence of the H_2_O signal in the ^1^H MAS NMR spectrum (Figure S22.iv),^47^ where a signal at 2.2 ppm arising from the μ_3_–OH sites is now clearly visible. The PXRD pattern of the activated sample, Zr_6_(BDC)_4_(TDC)_2_, confirms that the framework retains its crystallinity and closely resembles the pattern of Zr_6_(BDC)_4_(TDC)_2_-DMF (Figure 7a.iv). Rietveld refinement of this pattern with the two structural models described above showed that Zr_6_(BDC)_4_(TDC)_2_ has the same arrangement of linkers as Zr_6_(BDC)_4_(TDC)_2_-DMF, with TDC linking clusters through Zr–O coordination bonds (Figure S11). The movement of TDC from the site occupied in Zr_6_(BDC)_4_(TDC)_2_-H_2_O back to the site of the as-made Zr_6_(BDC)_4_(TDC)_2_-DMF is confirmed by the ^13^C CP MAS spectra of Zr_6_(BDC)_4_(TDC)_2_, which shows that the signal for the TDC carboxylate C shifts from 171 to 168 ppm, as determined by spectral assignments aided by HETCOR (Figure S27). This movement is triggered by the removal of H_2_O capping ligands from the Zr_6_ cluster of Zr_6_(BDC)_4_(TDC)_2_-H_2_O during activation, which breaks the hydrogen bonds that stabilized the TDC and creates uncoordinated sites on the Zr_6_ clusters that attract the carboxylates of TDC. The rotation of Zr_6_ clusters in Zr_6_(BDC)_4_(TDC)_2_ increases to 10° from 7° in Zr_6_(BDC)_4_(TDC)_2_-H_2_O to accommodate the bent shape of TDC. Thus, the Zr_6_(TDC)_2_ layers are reformed, and Zr_6_(BDC)_4_(TDC)_2_ is described as a 12-c **fcu** net. The reintroduction of atmospheric water to the activated material again disconnects the TDC from the cluster, affording Zr_6_(BDC)_4_(TDC)_2_-H_2_O (Figure S28 for the variable-temperature PXRD data). Here, water acts in a manner similar to methanol, preferentially binding to the Zr_6_ clusters and securing the TDC linker in the framework with hydrogen bonding. Similarly to methanol, water has been reported for postsynthetic linker exchange and linker removal.^48,49^ This reversible structural transformation demonstrates that the rotation of the Zr_6_ clusters, emerging from the direct combination with the two linkers in one-pot synthesis, can act as a structural degree of freedom triggered by the chemical control of the TDC position.

The N_2_ adsorption–desorption isotherm of Zr_6_(BDC)_4_(TDC)_2_ exhibits a type I profile, which is typical for microporous materials, with a BET surface area of 1054 m^2^/g and a pore volume of 0.41 cm^3^/g (Figures 9a and S29 for the pore size distribution). Even though these values are smaller than the values reported for UiO-66, 1290 m^2^/g and 0.49 cm^3^/g, respectively,^50^ they are in line with differences between the two MOFs, as one-third of the BDC linkers in UiO-66 are replaced by the shorter TDC in Zr_6_(BDC)_4_(TDC)_2_. The theoretical values of surface area and pore volume of Zr_6_(BDC)_4_(TDC)_2_ were determined using Zeo++^51^ to be 927 m^2^/g and 0.30 cm^3^/g, respectively, and the difference from the experimental measured values is attributed to the missing linkers. The Zr_6_(BDC)_4_(TDC)_2_ sample after N_2_ adsorption measurements was analyzed by TGA and ^1^H NMR after base digestion to derive the chemical formula Zr_6_O_4_(OH)_4_(BDC)_3.48_(TDC)_1.93_(OH)_1.18_, where the presence of ^–^OH as the only terminal coordinated ligands on Zr atoms was supported by solid-state ^1^H MAS NMR (Figure S22.iv). The total number of linkers per formula unit, 5.41, indicates that 10% of the linkers of the theoretical formula are missing, and they predominantly correspond to missing BDC. The proportion of missing linkers from the chemical analysis of Zr_6_(BDC)_4_(TDC)_2_ is similar to that observed for UiO-66 samples.^34,52^

The porous features of MOFs, often described as cages, are defined by their topology, and their exact size and shape are determined by the geometric characteristics of the linker. The porosity of Zr_6_(BDC)_4_(TDC)_2_ more closely resembles that of UiO-66 than DUT-67, as these two phases share the same underlying **fcu** topology, characterized by octahedral and tetrahedral cages, which are accessed through triangular windows. In contrast, the pore system of DUT-67, **reo** topology, consists of octahedral and cuboctahedral cages, and it is accessible through both triangular windows and square-shaped windows. Therefore, there is an alternative diffusion pathway for guest molecules within DUT-67 through only the square-shaped windows, which is not available in the **fcu** structures (Figure S30). In **fcu** Zr_6_(BDC)_4_(TDC)_2_, the windows are composed of two BDC and one TDC linker, and they are smaller than those in UiO-66. The bent shape of the TDC results in two different arrangements with respect to BDC and two types of triangular windows. One window has the sulfur atom of the thiophene ring pointing away from the two BDC linkers (Figure 8a), while the other has the thiophene ring pointing toward the two BDC linkers (Figure 8b). The two BDC sites also affect the window shape. In particular, the displacement of BDC site 2, from the plane defined by Zr1 atoms of the two connected Zr_6_ clusters, creates a convex side in the wider window (Figure 8a) and a concave side in the narrower window (Figure 8b). Consequently, the tetrahedral cage of Zr_6_(BDC)_4_(TDC)_2_ is distorted from the regular shape adopted in UiO-66, with point symmetry 4̅3*m*, to an isosceles tetrahedron (Figure 8c) with point symmetry 2. The arrangement of the two windows on the octahedral cage affords a base built from four TDC linkers, creating an elongated accessible space (Figure 8d). This cage has a lower point symmetry, 222, compared to the *m*3̅*m* point symmetry of the regular octahedral cage in UiO-66. Therefore, the ordering of bent and straight linkers in Zr_6_(BDC)_4_(TDC)_2_ develops a pore system with unique features that cannot be depicted by the simple net representation of the **fcu** topology.

**Figure 8:**
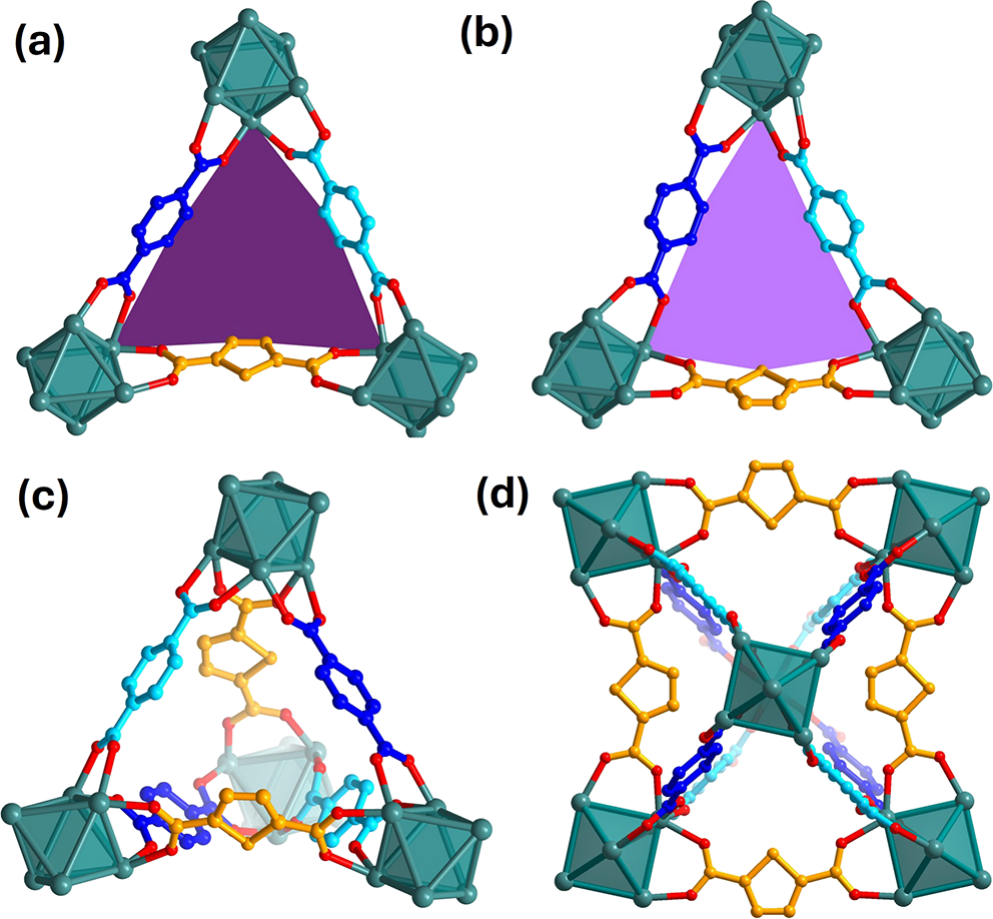
Shape of the triangular windows in Zr_6_(BDC)_4_(TDC)_2_ is determined by the orientation of TDC. (a) Wider triangular window with the thiophene ring of TDC pointing away from the BDC linkers. The shape of the window is illustrated by a dark purple distorted triangle. (b) Narrower triangular window with the thiophene ring pointing toward the BDC linkers. The shape of the window is illustrated by a light purple distorted triangle. The purple triangles are included to aid the visual differentiation between the two types of windows. (c) Tetrahedral cage of Zr_6_(BDC)_4_(TDC)_2_ is assembled by two windows of each type and has a distorted shape. (d) Octahedral cage of Zr_6_(BDC)_4_(TDC)_2_ consisting of four windows of each type, and its base has a rectangular shape. Zr is shown in teal, O is shown in red, the C atoms of BDC site 1 are in blue, the C atoms of BDC site 2 are in cyan, and TDC is shown in orange. The hydroxides and oxides on the Zr_6_ cluster and H atoms are omitted for clarity.

Zr_6_(BDC)_4_(TDC)_2_ was produced at a 0.5 g scale (see Scaled up synthesis in the Supporting Information) and utilized to pack a chromatography column for testing the separation of *n*-hexane, cyclohexane, and benzene (see Separation in Gas Chromatography in the Supporting Information). This application explores the effect of Zr_6_(BDC)_4_(TDC)_2_ cages on guests with different shapes and evaluates the material for the important separation of cyclohexane and benzene, which is challenging in typical distillation setups. Zr_6_(BDC)_4_(TDC)_2_ separates the mixture into three clear fractions under optimized conditions, whereby the column temperature is kept isothermal at 250 °C. Zr_6_(BDC)_4_(TDC)_2_ exhibits reverse shape selectivity, with *n*-hexane eluting first, followed by benzene and cyclohexane (Figure 9). The concept of reverse shape selectivity was described for zeolites, contrasting the usual selectivity preference for linear hydrocarbons; this phenomenon has been observed, for example, in SAPO-5 and MCM-22.^53–56^ This selectivity has been widely reported for UiO-66, where it is attributed to the greater rotational freedom of more compact hydrocarbons within framework cages, resulting in their increased retention time and preferential adsorption compared to longer linear hydrocarbons.^57–59^ The same rationale can be applied to understand the observed reverse shape selectivity of the Zr_6_(BDC)_4_(TDC)_2_ system. Denayer et al. reported the results from a packed GC column containing UiO-66, and the results of Zr_6_(BDC)_4_(TDC)_2_ followed a similar trend.^60,61^ The thermodynamic parameters indicate stronger separation performance for Zr_6_(BDC)_4_(TDC)_2_, with slightly larger differences in Gibbs free energy between benzene/cyclohexane and cyclohexane/*n*-hexane compared to the reported values for UiO-66 (Tables S13–S15 and Figure S26). We attribute these changes in selectivity to the unique cage and window geometries of Zr_6_(BDC)_4_(TDC)_2_, resulting from the ordering of the two linkers with differing geometries. Single-component adsorption isotherms of *n*-hexane, benzene, and cyclohexane were measured at 25 °C compared to the higher temperatures of the GC column; however, the differences in kinetics among the three guests (*n*-hexane > benzene > cyclohexane) are consistent with the chromatographic results (Figure S32).

**Figure 9:**
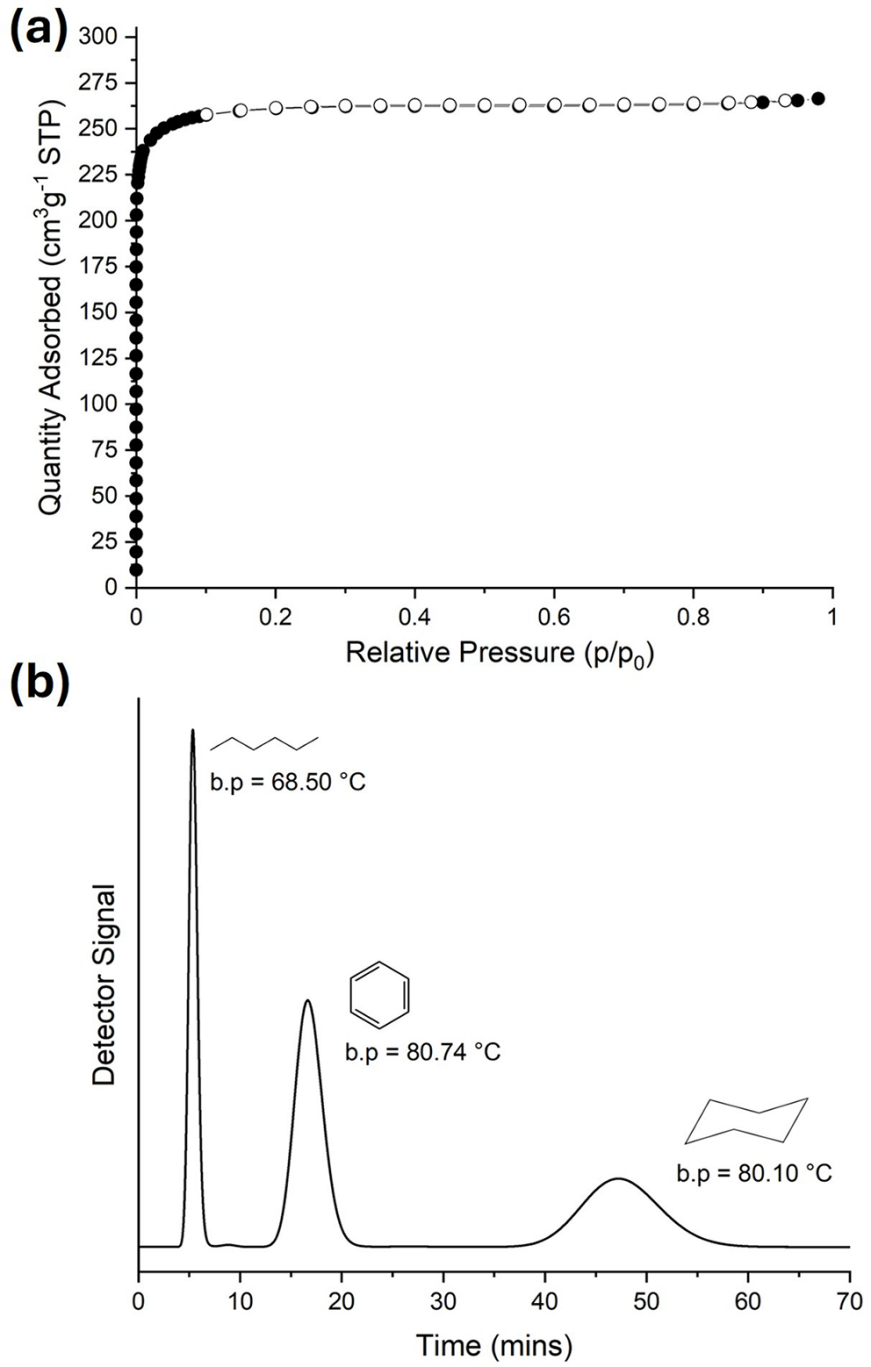
(a) N_2_ adsorption–desorption isotherm of Zr_6_(BDC)_4_(TDC)_2_. The closed symbols correspond to the adsorption branch, and the open symbols correspond to the desorption branch. (b) Chromatographic separation of *n*-hexane, cyclohexane, and benzene by a column packed with Zr_6_(BDC)_4_(TDC)_2_. The column temperature was kept isothermal at 250 °C during the separation.

We used molecular dynamics (MD) simulations to explore how the differences in window shapes between Zr_6_(BDC)_4_(TDC)_2_ and UiO-66 affect the separation of *n-*hexane, benzene, and cyclohexane (see the Molecular Dynamics Simulations section of the Supporting Information). First, we placed guest molecules in the tetrahedral cages of each MOF and monitored their displacement over time. In all simulations, the guest molecules remained within the tetrahedral cages, and no diffusion events were observed over 50 ps. These simulations indicate that, in both MOFs, the three guests preferentially adsorb in the tetrahedral cages and, at *T* = 523 K, reside there over 95% of the time, consistent with previous reports on UiO-66.^62^ In the second set of simulations, we placed guest molecules at the center of each octahedral cage and observed how long it takes for them to enter one of the neighboring tetrahedral cages. Table S16 shows that the time it takes for each guest to pass from octahedral to tetrahedral cages in Zr_6_(BDC)_4_(TDC)_2_ follows the same order as their separation behavior: *n-*hexane passes first, and cyclohexane migrates last. In contrast, in UiO-66, while *n-*hexane also migrates first, benzene and cyclohexane exhibit similar migration times. Overall, cyclohexane requires significantly more time to enter the tetrahedral cage in Zr_6_(BDC)_4_(TDC)_2_ than for any other guest molecule in either MOF, indicating an enhanced kinetic-based separation of *n-*hexane/benzene/cyclohexane due to the smaller triangular windows in Zr_6_(BDC)_4_(TDC)_2_ compared to those of UiO-66. When analyzing the guest trajectories in Zr_6_(BDC)_4_(TDC)_2_, it is observed that the guests preferentially use one of the two windows, the wider one (Figure 8a) instead of the narrower one (Figure 8b). Therefore, this selective usage restricts the available migration pathways to the tetrahedral cage compared to UiO-66, which features only one window type (Table S16). These simulation results align well with the experimental findings for Zr_6_(BDC)_4_(TDC)_2_ and demonstrate the effect of ordering two linkers with different sizes and shapes on the separation performance.

## Conclusions

A new two-linker ordered Zr-MOF containing both bent and straight linkers was identified by high-throughput experimental screening of the ZrOCl_2_·8H_2_O/BDC/TDC/FA/DMF chemical space. Zr_6_(BDC)_4_(TDC)_2_-DMF is formed as a pure phase in a wide region of this space, despite competition from the two MOFs formed by each linker individually. Its structure and reactivity reflect the synergy between the geometries of the linear BDC and bent TDC linkers. TDC is only known in frameworks with less than the complete 12-fold connectivity of the parent UiO-66 because of the requirement for the clusters to rotate about multiple axes in order for TDC to connect them, preventing complete bridging of the clusters by 12 ditopic linkers. Here, the bent TDC is accommodated within the 12-c **fcu** net by coordinating to the equatorial plane of the Zr_6_ clusters, which rotate about the axis defined by their two axial vertices to afford a Zr_6_(TDC)_2_ layer that matches the geometry of the linker. This single rotation defines two distinct sites for the BDC linkers, one displaced from and the other inclined to the single BDC site in UiO-66, that connect these layers to the 12-c Zr_6_(BDC)_4_(TDC)_2_ framework. The combination of the bent shape of TDC and the straight BDC induces distortions in the tetrahedral and octahedral cages of the **fcu** net, resulting in unique pore shapes, which are utilized for the separation of a *n*-hexane/benzene/cyclohexane mixture and reflect the ability of multiple linkers to tailor the porosity of established framework topologies.

The displaced BDC creates a second environment for TDC, which it occupies upon the addition of a protic solvent. The protic solvent displaces TDC from its original position, coordinating to the equatorial Zr sites, to the new location and thus forming an 8-c net. Here, the thiophene ring of TDC interacts favorably with the aromatic ring of the displaced BDC, while its carboxylate groups form hydrogen bonds both the cluster hydroxides and cluster-bound protic ligands, in an environment rich in noncovalent interactions that originally arises from the synergy between the linker geometries that enables the formation of the original 12-c net. This reversible transition is an intrinsic property of the Zr_6_(BDC)_4_(TDC)_2_ structure, which allows the disconnection of one linker while the rest of the framework remains intact, affording an environment that retains the disconnected linker within the structure available for the reconnection step. The ordering of multiple linkers to afford new MOF structures by decorating parent frameworks can be delivered by the efficient exploration of large chemical spaces through high-throughput experimental workflows, yielding structural motifs and reactivity patterns beyond those readily envisaged from the structures of single-linker MOFs.

## Supplementary Material



## Data Availability

The data underlying this study is available in the University of Liverpool Data Repository at 10.17638/datacat.liverpool.ac.uk/2986.

## Notes

Experimental procedures and characterization data, materials and general procedures, PXRD patterns, further structural refinement details, additional structure figures, sorption isotherms, and gas chromatography data (PDF)
