# Supplementary material for: Ordering Bent and Straight Dicarboxylate Linkers in an fcu Zirconium Metal–Organic Framework
Source: J Am Chem Soc. 2025 Jul 23;147(31):27586–98. doi: 10.1021/jacs.5c05854 (PMC12333367; doi:10.1021/jacs.5c05854)
Supplement: Supplementary file 1 [file ja5c05854_si_001.pdf]

Supporting Information

Ordering Bent and Straight Dicarboxylate Linkers in an fcu Zirconium Metal-Organic Framework

Grace S. G. Farmer[1,2], Daniel J. Cheney[1], Khai-Nghi Truong[3], Nusik Gedikoglu[1,2], Bhupendra P. Mali[1], Datta Markad[1], Dmytro Antypov[1,2], Frédéric Blanc[1,2,4], Alexandros P. Katsoulidis[1], and Matthew J. Rosseinsky[1,2]*

[1] Department of Chemistry, University of Liverpool, Crown Street, L69 7ZD Liverpool, UK,

[2]Leverhulme Research Centre for Functional Materials Design, Materials Innovation Factory, University of Liverpool, L7 3NY Liverpool, UK

[3]Rigaku Europe SE, Hugenottenallee 167, 63263 Neu-Isenburg, Germany

[4]Stephenson Institute for Renewable Energy, University of Liverpool, L69 7ZF Liverpool, UK

*Corresponding Author: m.j.rosseinsky@liverpool.ac.uk

Contents

Experimental Section 3
General Experimental Details 3
MOF synthesis with liquid-handling robots 3
Scaled up synthesis of $Zr_6(BDC)_4(TDC)_2$-DMF 4
Shorter reaction synthesis of $Zr_6(BDC)_4(TDC)_2$-DMF 4
Solvent exchange and activation 5
Names of compounds 6
Characterisation Techniques 7
Separation in Gas Chromatography 9
Batch 1 Synthesis Tables and PXRD Data (Tables S1-S2 and Figure S1) 10
Batch 2 Synthesis Tables and PXRD Data (Tables S3-S4 and Figure S2-S3) 14
^{1}H NMR Data of Batch 1 and 2 (Table S5 and Figure S4) 19
SEM image of the crystals (Figure S5) 21
Structure Determination 22
$Zr_6(BDC)_4(TDC)_2$-DMF (Figures S6-S7) 22
$Zr_6(BDC)_4(TDC)_2$-MeOH (Figure S8) 25
3D Electron Diffraction of $Zr_6(BDC)_4(TDC)_2$-H_2O (Figure S9) 26
$Zr_6(BDC)_4(TDC)_2$ (Figures S10-11) 28
Crystallographic Data Tables (Tables S6 –S9) 31
Further Details of the Crystal Structures (Figures S12 – S15) 35
Chemical Formula of Bulk Samples (Figures S16 – S19) 38
^{1}H NMR Data to Monitor the Replacement of Methanol by Atmospheric Water (Table S10 and Figure S20) 44
Shorter Reaction Synthesis Results and PXRD Data (Table S11 and Figure S21) 46
Solid State MAS NMR Spectra (Figures S22 – S27 and Table S12) 48
Variable temperature PXRD data of $Zr_6(BDC)_4(TDC)_2$-H_2O (Figure S28) 55
Pore size distribution of $Zr_6(BDC)_4(TDC)_2$ (Figure S29) 57
Porous Features of UiO-66, DUT-67, and $Zr_6(BDC)_4(TDC)_2$ (Figure S30) 58
Gas Chromatography of $Zr_6(BDC)_4(TDC)_2$ (Tables S13– S15 and Figure S31) 59
Vapour adsorption-desorption isotherms (Figure S32) 63
Molecular Dynamics Simulations (Table S16) 65
References 66

Experimental Section

General Experimental Details

All chemicals were purchased from commercial sources and used without further purification. Zirconyl chloride octahydrate ($ZrOCl_2$•$8H_2O$, purity 98%), 1,4-benzene dicarboxylic acid (BDC, purity ≥98%), sodium hydroxide-d (40 wt. % in D_2O) and deuterium oxide (99.9 atom % D) were obtained from Sigma-Aldrich. 2,5-Thiophene dicarboxylic acid (TDC, purity ≥98%) was obtained from Tokyo Chemical Industry Co. *N,N*-Dimethylformamide (DMF), methanol (MeOH), and tetrahydrofuran (THF) were obtained from Fisher Scientific. Formic acid (purity 97%) was obtained from Alfa Aesar.

MOF synthesis with liquid-handling robots

Multiple linker Zr-based MOFs with BDC and TDC linkers, were prepared in two batches with automation liquid dispensation. The following procedure has been adapted from the workflow as reported by A.Tollitt.[1] Stock solutions were made of the reaction components and they were transferred to a 20 mL headspace screw neck glass vial, by automated dispensation. Solutions were all in DMF, aside from formic acid, which was dispensed neat. For the batch syntheses, stock solutions of $ZrOCl_2$•$8H_2O$ was dissolved in DMF (0.0225 M), the concentration was chosen to ensure a homogenous solution for the duration of the liquid dispensation (up to 2.5 hours). The volume of 0.0225 M $ZrOCl_2$•$8H_2O$ stock solution dispensed into each reaction mixture was fixed, therefore there was 38 mg (0.12 mmol) in each 10 mL scale reaction. The linker stock solutions were prepared by dissolving BDC and TDC in DMF at a total linker, (BDC+TDC), concentration of 0.15 M, with different BDC:TDC molar ratios. The different reaction mixtures were prepared using the Eppendorf epMotion 5075t liquid handling platform, which dispensed into the reaction vessel. The individual reaction mixtures were prepared in parallel and the components were added to the mixture in the same order (formic acid – $ZrOCl_2$•$8H_2O$ stock – BDC:TDC linker stock – DMF) for both batches. DMF was dispensed into each vial to ensure each reaction had a total reaction solution volume of 10 mL. The vials were manually sealed with metal screw caps, and heated to 120 °C with a ramp rate of 5 °C/min. The vials were held at this temperature for 48 hours, and then ramped down to 25 °C with a rate of 0.1 °C/min. The vials and caps were purchased from VWR International.

Scaled up synthesis of $Zr_6(BDC)_4(TDC)_2$-DMF

Synthesis of a selected composition was scaled up to a total liquid amount of 100 mL in a 500 mL Duran reaction bottle. The composition of the selected point was Zr:(BDC + TDC) = 0.67:0.33, BDC:TDC = 0.57:0.43, and FA:Zr = 440. $ZrOCl_2 \bullet 8H_2O$ (1.2 mmol, 0.3867 g), BDC (1.03 mmol, 0.1705 g), TDC (0.77 mmol, 0.1333 g) were weighed and combined. DMF (80.1 mL) was added, and the solution was sonicated for five minutes, resulting in a clear solution. The formic acid (19.9 mL) was added, and the resulting solution was sonicated for five minutes to ensure homogeneity of the solution. The Duran reaction bottle was then sealed with a screw lid, before being heated at 120 °C for 48 hours. The ramping rate of heating was 5 °C/min, and the cooling rate was 0.1 °C/min. The powder product (0.5 g) was checked for phase purity with powder x-ray diffraction, which was consistent with the product obtained from the 10 mL scale robotic synthesis, then washed and used for further analysis. This synthesis was repeated several times to obtain enough material for further measurements.

Shorter reaction synthesis of $Zr_6(BDC)_4(TDC)_2$-DMF

The robotic platform was used to prepare 18 vials with the synthesis composition of Zr:(BDC + TDC) = 0.67:0.33, BDC:TDC = 0.57:0.43, and FA:Zr = 440. This is the same point that is discussed in detail in the main text. The synthesis procedure followed was as described in supplementary information section, 'MOF synthesis with liquid-handling robots'. Five ovens were used, and three vials were placed into each oven. Three vials were chosen per quenching temperature to ensure enough material for PXRD measurements. All of the ovens were heated to 120 °C with a ramping rate of 5 °C/min. At different times, detailed in Table S11, the vials were quenched to room temperature by removing them from the respective oven. A control experiment was also carried out, which included the normal ramping down rate (0.1 °C/min) as controlled by the oven. The summary of the observations of the reactions are provided in Table S11. The PXRD patterns are also provided below (Figure S21).

Solvent exchange and activation

The material was collected by centrifugation to start the washing procedure. The supernatant was decanted, replaced with DMF (25 mL) and the mixture was stirred for 1 hour. The process of washing with DMF was repeated three times over 24 hours. The DMF was then decanted after centrifugation, and the material was stirred in methanol (25 mL), for a total of 8 days, during which the methanol was replaced 9 times. During the methanol washing, the material was collected by centrifugation, solvent decanted, and the material was allowed to dry, to be handled as a powder. At this point, liquid phase ^{1}H nuclear magnetic resonance (NMR) was obtained after the digestion of a small amount of material in NaOD and D_2O, this confirmed the completion of the DMF removal and the presence of MeOH in the pores (Figure S16). When the sample is left drying under ambient conditions (22 °C), subsequent NMR measurements show that the MeOH evaporates. To monitor the replacement of methanol by atmospheric water, $Zr_6(BDC)_4(TDC)_2$ -MeOH was prepared as described in 'Scaled up synthesis of $Zr_6(BDC)_4(TDC)_2$ -DMF'. Once the methanol washing was complete, the methanol was poured out of the centrifuge tube, and the loss of the methanol from the solid was monitored by ^{1}H solution NMR of the digested framework. The ^{1}H NMR spectra measured are also provided in Figure S20. Table S10 provides details of the timings and results of the measurements, namely the integration of the methanol solvent signal for each spectrum are also provided in Table S10. The results demonstrate that much of the methanol, expected to be exist on the surface of the solid and within the pores of the framework, is lost within 30 minutes. However a smaller amount of methanol remains in the sample for longer, which then is replaced by water overnight resulting in $Zr_6(BDC)_4(TDC)_2$ -H_2O.

These results pertain to a sample of 0.5 g, however we expect the process to occur more rapidly for smaller samples which are spread over a larger area. ^{1}H and ^{13}C magic angle spinning (MAS) NMR confirms this and shows the presence of water in the pores (Figure S22). The powder was subsequently activated under vacuum (10^{-3} mbar) at 150 °C for 16 hours to remove all guest species. A N_2 isotherm was measured on the activated material.

The sample used for the ^{1}H and ^{13}C MAS NMR spectra of $Zr_6(BDC)_4(TDC)_2$-DMF, was prepared as follows. The as-made solid was washed with DMF three times over 24 hours, then the supernatant DMF was replaced with THF which was immediately decanted after centrifugation. The sample was dried under a N_2 gas flow and then packed into the rotor for ^{1}H and ^{13}C MAS NMR measurements. THF is a non-protic solvent and has a lower boiling point compared to DMF, and as such, allows for the preparation of a material without the structural transition observed from methanol washing.

Names of compounds

The new compounds presented in this work are named as **$Zr_6(BDC)_4(TDC)_2$-guest** because in all cases the ideal formula of the framework (without counting defects) contains one $Zr_6O_4(OH)_4$ cluster, four BDC linkers, and two TDC linkers. As the structure of the material changes after treatment with different solvents the name of each compound includes the solvent that is incorporated as guest in the pores.

$Zr_6(BDC)_4(TDC)_2$-DMF: The as-made material that contains DMF in the pores of the framework.

$Zr_6(BDC)_4(TDC)_2$-MeOH: The material after the treatment in methanol and the complete exchange of DMF.

$Zr_6(BDC)_4(TDC)_2$-H_2O: The material after the exchange of methanol with atmospheric water.

$Zr_6(BDC)_4(TDC)_2$: Activated material without any guests in the pores.

Characterisation Techniques

Liquid state NMR spectroscopy. All ^1H NMR spectra were recorded in solution using either a Bruker Avance III HD 400 MHz or a Bruker Avance III HD 500 MHz NMR spectrometer. Spectra were recorded in a solvent mixture containing D_2O (640 µL) and NaOD (60 µL). Chemical shifts are in ppm, and with the residual solvent peak of D_2O (4.79 ppm). ^1H NMR measurements were used to determine the ratio of incorporated BDC and TDC linkers in the obtained solids from the relative integrations of their corresponding peaks.

Thermal analysis. Thermal Gravimetric Analysis (TGA) was performed under air atmosphere on a TA Instruments Q600 or a TA Instruments Q500 between 25 and 800 °C, with a scan rate of 5 °C min^{-1} and gas flow of 60 mL min^{-1}.

Gas sorption analysis. Nitrogen adsorption-desorption isotherms were collected at 77 K on a Micrometrics 3Flex Surface Characterisation. Samples were heated under vacuum to 150 °C for 16 hours, to remove guest species prior to the sorption measurement as detailed in the experimental section. The BET surface area was calculated from the adsorption branch of the isotherm using the pressure range $0.003 < P/P_0 < 0.04$, which was selected using the consistency criteria. The total pore volume was determined at P/P_0 of 0.95.

The theoretical values for $Zr_6(BDC)_4(TDC)_2$ were obtained using Zeo++ for a spherical probe of 3.64 Å in diameter (the kinetic diameter of a dinitrogen molecule) for reported CIF file of $Zr_6(BDC)_4(TDC)_2$.[2]

Scanning Electron Microscopy (SEM) imaging SEM was performed on a Hitachi S4800 cold-cathode field-emission electron microscope.

Laboratory PXRD. Laboratory based powder X-ray diffraction data were collected at room temperature on Bruker D8 Advance diffractometers with a monochromated Cu radiation source (Cu $K_{\alpha1}$, $\lambda = 1.5406$ Å). The measurements were run in transmission mode and the samples were placed either in capillaries with the liquid guest of each phase, or in the powder specimen holders as obtained from Bruker. The wet powder was placed between two sheets of mylar film and sealed within the batch mode holder for measurement.

Synchrotron PXRD. Synchrotron powder X-ray diffraction data were collected at the I11 beam line at Diamond Light Source. Data were collected at room temperature using the Position Sensitive Detector (PSD, Mythen-2), with the samples sealed in borosilicate capillaries. The capillaries containing the activated material (guest-free) were sealed in an Ar-filled glovebox. The PXRD structural refinement details are discussed in further detail in the structure determination section of the SI. Variable temperature PXRD data was collected using the cryo and PSD detector on open-ended capillaries with the powder secured by a small amount of glass wool.

Three-dimensional electron diffraction crystal structure determination. Electron diffraction measurements for $Zr_6(BDC)_4(TDC)_2$-H_2O were collected using a Rigaku XtaLAB Synergy-ED equipped with a Rigaku HyPix-ED detector optimised for operation in the continuous rotation 3D ED experimental setup.[3, 4] The instrument was operated and the diffraction data were processed in the program CrysAlis^{Pro}.[5] A multi-scan absorption correction was performed using spherical harmonics implemented in SCALE3 ABSPACK scaling algorithm in CrysAlis^{Pro}. The structure was solved using ShelXT,[6] and subsequently, refined with kinematical approximation using ShelXL[7] in the crystallographic program suite Olex2.[8, 9] A solution of 5 mg of MOF and 20 mL of methanol was sonicated for 20 minutes, then a few drops of the solution were left to airdry on a grid before handling in the cryo-transfer system prior to measurement. Cryo-transfer means freezing of samples prior to introduction to vacuum. It was applied using a Gatan ELSA (Model 698) specimen holder measuring at 100 K. As electron diffraction requires samples to be studied under high vacuum, the cryo-transfer technique is essential for many sensitive compounds, such as solvent-containing MOFs or proteins. Next to stabilization in vacuo, other benefits are improving resolution, reducing disorder, and reducing beam damage. The structure determination of electron diffraction data section includes further details on the refinement model.

Solid state NMR. Solid State magic-angle spinning (MAS) NMR was performed using a Bruker Avance III HD 400 MHz NMR spectrometer equipped with a 4 mm triple-resonance HXY MAS probe in double-resonance mode tuned to ^1H at $\upsilon_0(^1H)$ = 400.1 MHz and ^{13}C at $\upsilon_0(^{13}C)$ = 100.6 MHz. The samples were packed into 4 mm ZrO_2 rotors and spun at a MAS rate of υ_r = 10 kHz. ^1H NMR spectra were detected using the Hahn echo experiment, synchronized to a single rotor period. The ^1H and ^{13}C radio frequency (rf) field amplitudes for 90° pulses were 52.6 kHz and 45.4 kHz, respectively. $^1H \rightarrow {}^{13}C$ cross polarisation (CP) experiments were carried out using Hartman-Hahn matched rf field amplitudes of 48 kHz (with a 70 to 100% linear amplitude ramp) and 27.8 kHz for ^1H and ^{13}C, respectively. The CP contact times were varied between 50 µs and 2 ms. SPINAL-64 decoupling[10] was applied during ^{13}C NMR signal acquisition with a ^1H rf field amplitude of 52.6 kHz. The CP experiments used a recycle delay of at least 1.3 times the ^1H spin-lattice relaxation time (T_1), as measured using saturation recovery, while quantitative directly excited ^{13}C spectra used a recycle delay of 5 times the ^{13}C T_1, as measured using CP inversion. The ^1H and ^{13}C spectra were externally referenced to adamantane at 1.8 ppm[11,12] and 29.45[13] ppm, respectively. Two-dimensional (2D) HETeronuclear CORrelation (HETCOR) experiments were carried out by applying Frequency-Switched Lee-Goldburg (FSLG)[14] homonuclear decoupling at a rf field amplitude of 68 kHz and a LG offset of 0 Hz during the ^1H evolution time. A ^1H chemical shift scaling factor, λ_{exp}, of 0.7 was obtained by comparing the ^1H spectrum of L-alanine to its HETCOR projection, which was then used to recover the full ^1H chemical shifts from the scaled-down shifts resulting from FSLG decoupling.

Vapour adsorption. The three vapour isotherms were collected by using an IGA system (Intelligent Gravimetric Analyzer), Hiden Isochema Ltd., Warrington, UK). The hexane and cyclohexane measurements were ran with HISORP Systems Software Version 5, and the benzene measurement with IGASwin Systems Software V1.05.12. Prior to each isotherm collection, the MOF sample was activated at 150 °C under dynamic vacuum for 16 hours. The

measurements of hexane and benzene both had a maximum of 180 minutes per pressure point measured. However, owing to the slower kinetics of cyclohexane in the MOF, the maximum time was increased to 10 hours for the first 3 pressure points (where the majority of the uptake occurred), following those points the maximum time was 180 minutes which was suitable to reach equilibrium. The pressure ramp rate was 1.66 mbar/minute for all measurements.

Separation in Gas Chromatography

All separations were carried out using an Agilent 6890N gas chromatographic system equipped with a flame ionisation detector (FID). Helium was the carrier gas for all cases. The data evaluation was conducted with the ChemStation software from Agilent Technologies.

The material for the column was prepared as follows: the activated material was pelletised under 5 tonne pressure. The 2.5 cm wide pellet was carefully broken, and sieved with a 80/100 mesh to give 149-177 µm sized binderless pellets of the MOF. 485 mg of the material was used to pack a stainless-steel column of 22 cm with an internal diameter of 2.1 mm. Prior to the measurements, the $Zr_6BDC_4TDC_2$ column was activated and conditioned by heating to 200 °C at a heating rate of 2 °C min^{-1} in helium flow and keeping this final temperature for 12 hours. This was followed by heating to 250 °C at a heating rate of 2 °C min^{-1} in helium flow and keeping this final temperature for 3 hours. This was to ensure that the material was fully desolvated. An inert carrier gas (He) was used to inject liquid samples (0.2 µL) of equimolar hexane/cyclohexane/benzene mixtures into a chromatographic column, and a flame ionisation detector (FID) was used to measure the response curve. The optimised temperature for the separation was 250 °C.

Variable temperature pulse gas chromatography: Gas-phase adsorption at zero surface coverage was studied using the pulse chromatographic technique employing a gas chromatograph and the $Zr_6(BDC)_4(TDC)_2$ column. The individual injections were injected separately in order to accurately determine their retention time at four different temperatures (493.15 K, 503.15 K, 513.15 K, 523.15 K). The dead volume of the system was calculated using the retention time of methane at the four different temperatures. The results were used to calculate the thermodynamic parameters of adsorption for hexane, cyclohexane, and benzene on $Zr_6(BDC)_4(TDC)_2$.

Batch 1 Synthesis Tables and PXRD Data (Tables S1-S2 and Figure S1)

A stock solution of the metal source was prepared by dissolving $ZrOCl_2•8H_2O$ (7.882 mmol, 2.54 g) in DMF (300 mL). Linker stock solutions with five different BDC:TDC molar ratios, BDC:TDC = 0.75:0.25, 0.67:0.33, 0.5:0.5, 0.33:0.67 and 0.25:0.75, were prepared at a total linker (BDC+TDC) concentration of 0.15 M in DMF (25 mL). The quantities of BDC and TDC used in each solution are in Table S1.

Table S1 Compositions, BDC:TDC, of stock solutions used in the first batch synthesis and the quantities of BDC and TDC used to prepare 0.15 M solutions of each in 25 mL of DMF.

BDC:TDC	BDC		TDC	
Molar Ratio	mmol	g	mmol	g
0.75:0.25	2.81	0.47	0.94	0.16
0.67:0.33	2.48	0.41	1.24	0.21
0.5:0.5	1.88	0.31	1.88	0.32
0.33:0.67	1.24	0.21	2.48	0.43
0.25:0.75	0.94	0.16	2.81	0.48

The order of addition for the reaction components was: formic acid – $ZrOCl_2•8H_2O$ stock – BDC:TDC linker stock – DMF, and this was fixed for each of the 60 individual reaction mixtures in the batch, with the process of automated dispensation lasting 2 hours. Selected powder products were collected by centrifugation and washed as discussed in 'Solvent Exchange and Activation'.

The synthesis compositions of the 60 reaction mixtures for batch 1 synthesis exploring the system $ZrOCl_2·H_2O$/BDC/TDC/FA/DMF are given in Table S2. All the ratios (BDC:TDC, Zr:(BDC +TDC), FA:Zr) are molar ratios. The linker compositions varied from 0.75:0.25, 0.67:0.33, 0.50:0.50, 0.33:0.67, 0.25:0.75. The formic acid (FA) to $ZrOCl_2•8H_2O$ ratio varied by 500, 310, and 120. The $ZrOCl_2•8H_2O$ to combined linker ratio varied by 0.67:0.33, 0.50:0.50, 0.40:0.60, 0.33:0.67. The ratio 0.60:0.40 was not included as it would have made the batch too large (75 points). The results of batch 1 (Figure 1 and Figure S1) demonstrated that the interesting part of the chemical space was around the included 0.40:0.60 ratio and for this reason the 0.60:0.40 ratio was not included as part of batch two experiments. Although, we recognise that in other systems it might have been necessary to explore this ratio. The HTS exploration is not implemented here as a brute force methodology, instead it is built on as each system requires careful consideration on the selection of reaction compositions.

Table S2 Compositions of the 60 reaction mixtures. For each composition, the molar quantities of the components used are given, along with the BDC:TDC molar ratio of the BDC:TDC linker stock solution used and the volume of each stock solution dispensed. Numbers are given to two decimal places, aside from the BDC (mmol) and TDC (mmol) data columns which are given to three decimal places. Compositions of the pure phase, as marked by stars in Figure 1 and Figure S1, are highlighted in green.

Zr:(BDC +TDC)	BDC:TDC (molar)	FA:Zr	$ZrOCl_2$ •$8H_2O$ (mmol)	BDC (mmol)	TDC (mmol)	$ZrOCl_2$ •$8H_2O$ Stock (mL)	BDC:TDC Stock (mL)	FA (mL)	DMF (mL)	Solid? (Y/N)
0.67:0.33	0.25:0.75	120	0.12	0.015	0.045	5.33	0.4	0.54	3.73	Y
0.67:0.33	0.25:0.75	310	0.12	0.015	0.045	5.33	0.4	1.40	2.87	Y
0.67:0.33	0.25:0.75	500	0.12	0.015	0.045	5.33	0.4	2.26	2.01	N
0.67:0.33	0.33:0.67	120	0.12	0.020	0.040	5.33	0.4	0.54	3.73	Y
0.67:0.33	0.33:0.67	310	0.12	0.020	0.040	5.33	0.4	1.40	2.87	Y
0.67:0.33	0.33:0.67	500	0.12	0.020	0.040	5.33	0.4	2.26	2.01	N
0.67:0.33	0.5:0.5	120	0.12	0.030	0.030	5.33	0.4	0.54	3.73	Y
0.67:0.33	0.5:0.5	310	0.12	0.030	0.030	5.33	0.4	1.40	2.87	Y
0.67:0.33	0.5:0.5	500	0.12	0.030	0.030	5.33	0.4	2.26	2.01	N
0.67:0.33	0.67:0.33	120	0.12	0.040	0.020	5.33	0.4	0.54	3.73	Y
0.67:0.33	0.67:0.33	310	0.12	0.040	0.020	5.33	0.4	1.40	2.87	Y
0.67:0.33	0.67:0.33	500	0.12	0.040	0.020	5.33	0.4	2.26	2.01	N
0.67:0.33	0.75:0.25	120	0.12	0.045	0.015	5.33	0.4	0.54	3.73	Y
0.67:0.33	0.75:0.25	310	0.12	0.045	0.015	5.33	0.4	1.40	2.87	Y
0.67:0.33	0.75:0.25	500	0.12	0.045	0.015	5.33	0.4	2.26	2.01	N
0.50:0.50	0.25:0.75	120	0.12	0.030	0.090	5.33	0.8	0.54	3.33	Y
0.50:0.50	0.25:0.75	310	0.12	0.030	0.090	5.33	0.8	1.40	2.47	Y
0.50:0.50	0.25:0.75	500	0.12	0.030	0.090	5.33	0.8	2.26	1.61	Y
0.50:0.50	0.33:0.67	120	0.12	0.040	0.080	5.33	0.8	0.54	3.33	Y
0.50:0.50	0.33:0.67	310	0.12	0.040	0.080	5.33	0.8	1.40	2.47	Y
0.50:0.50	0.33:0.67	500	0.12	0.040	0.080	5.33	0.8	2.26	1.61	Y
0.50:0.50	0.5:0.5	120	0.12	0.060	0.060	5.33	0.8	0.54	3.33	Y
**0.50:0.50**	**0.5:0.5**	**310**	**0.12**	**0.060**	**0.060**	**5.33**	**0.8**	**1.40**	**2.47**	**Y**
0.50:0.50	0.5:0.5	500	0.12	0.060	0.060	5.33	0.8	2.26	1.61	Y
0.50:0.50	0.67:0.33	120	0.12	0.079	0.041	5.33	0.8	0.54	3.33	Y
0.50:0.50	0.67:0.33	310	0.12	0.079	0.041	5.33	0.8	1.40	2.47	Y
0.50:0.50	0.67:0.33	500	0.12	0.079	0.041	5.33	0.8	2.26	1.61	Y
0.50:0.50	0.75:0.25	120	0.12	0.090	0.030	5.33	0.8	0.54	3.33	Y
0.50:0.50	0.75:0.25	310	0.12	0.090	0.030	5.33	0.8	1.40	2.47	Y
0.50:0.50	0.75:0.25	500	0.12	0.090	0.030	5.33	0.8	2.26	1.61	Y
0.40:0.60	0.25:0.75	120	0.12	0.045	0.135	5.33	1.2	0.54	2.93	Y
0.40:0.60	0.25:0.75	310	0.12	0.045	0.135	5.33	1.2	1.40	2.07	Y
0.40:0.60	0.25:0.75	500	0.12	0.045	0.135	5.33	1.2	2.26	1.21	Y
0.40:0.60	0.33:0.67	120	0.12	0.059	0.121	5.33	1.2	0.54	2.93	Y
0.40:0.60	0.33:0.67	310	0.12	0.059	0.121	5.33	1.2	1.40	2.07	Y
0.40:0.60	0.33:0.67	500	0.12	0.059	0.121	5.33	1.2	2.26	1.21	Y
0.40:0.60	0.5:0.5	120	0.12	0.090	0.090	5.33	1.2	0.54	2.93	Y
**0.40:0.60**	**0.5:0.5**	**310**	**0.12**	**0.090**	**0.090**	**5.33**	**1.2**	**1.40**	**2.07**	**Y**
**0.40:0.60**	**0.5:0.5**	**500**	**0.12**	**0.090**	**0.090**	**5.33**	**1.2**	**2.26**	**1.21**	**Y**
0.40:0.60	0.67:0.33	120	0.12	0.119	0.061	5.33	1.2	0.54	2.93	Y
0.40:0.60	0.67:0.33	310	0.12	0.119	0.061	5.33	1.2	1.40	2.07	Y
0.40:0.60	0.67:0.33	500	0.12	0.119	0.061	5.33	1.2	2.26	1.21	Y
0.40:0.60	0.75:0.25	120	0.12	0.135	0.045	5.33	1.2	0.54	2.93	Y
0.40:0.60	0.75:0.25	310	0.12	0.135	0.045	5.33	1.2	1.40	2.07	Y
0.40:0.60	0.75:0.25	500	0.12	0.135	0.045	5.33	1.2	2.26	1.21	Y
0.33:0.67	0.25:0.75	120	0.12	0.060	0.180	5.33	1.6	0.54	2.53	Y
0.33:0.67	0.25:0.75	310	0.12	0.060	0.180	5.33	1.6	1.40	1.67	Y

0.33:0.67	0.25:0.75	500	0.12	0.060	0.180	5.33	1.6	2.26	0.81	Y
0.33:0.67	0.33:0.67	120	0.12	0.079	0.161	5.33	1.6	0.54	2.53	Y
0.33:0.67	0.33:0.67	310	0.12	0.079	0.161	5.33	1.6	1.40	1.67	Y
0.33:0.67	0.33:0.67	500	0.12	0.079	0.161	5.33	1.6	2.26	0.81	Y
0.33:0.67	0.5:0.5	120	0.12	0.120	0.120	5.33	1.6	0.54	2.53	Y
0.33:0.67	0.5:0.5	310	0.12	0.120	0.120	5.33	1.6	1.40	1.67	Y
**0.33:0.67**	**0.5:0.5**	**500**	**0.12**	**0.120**	**0.120**	**5.33**	**1.6**	**2.26**	**0.81**	**Y**
0.33:0.67	0.67:0.33	120	0.12	0.158	0.082	5.33	1.6	0.54	2.53	Y
0.33:0.67	0.67:0.33	310	0.12	0.158	0.082	5.33	1.6	1.40	1.67	Y
0.33:0.67	0.67:0.33	500	0.12	0.158	0.082	5.33	1.6	2.26	0.81	Y
0.33:0.67	0.75:0.25	120	0.12	0.180	0.060	5.33	1.6	0.54	2.53	Y
0.33:0.67	0.75:0.25	310	0.12	0.180	0.060	5.33	1.6	1.40	1.67	Y
0.33:0.67	0.75:0.25	500	0.12	0.180	0.060	5.33	1.6	2.26	0.81	Y

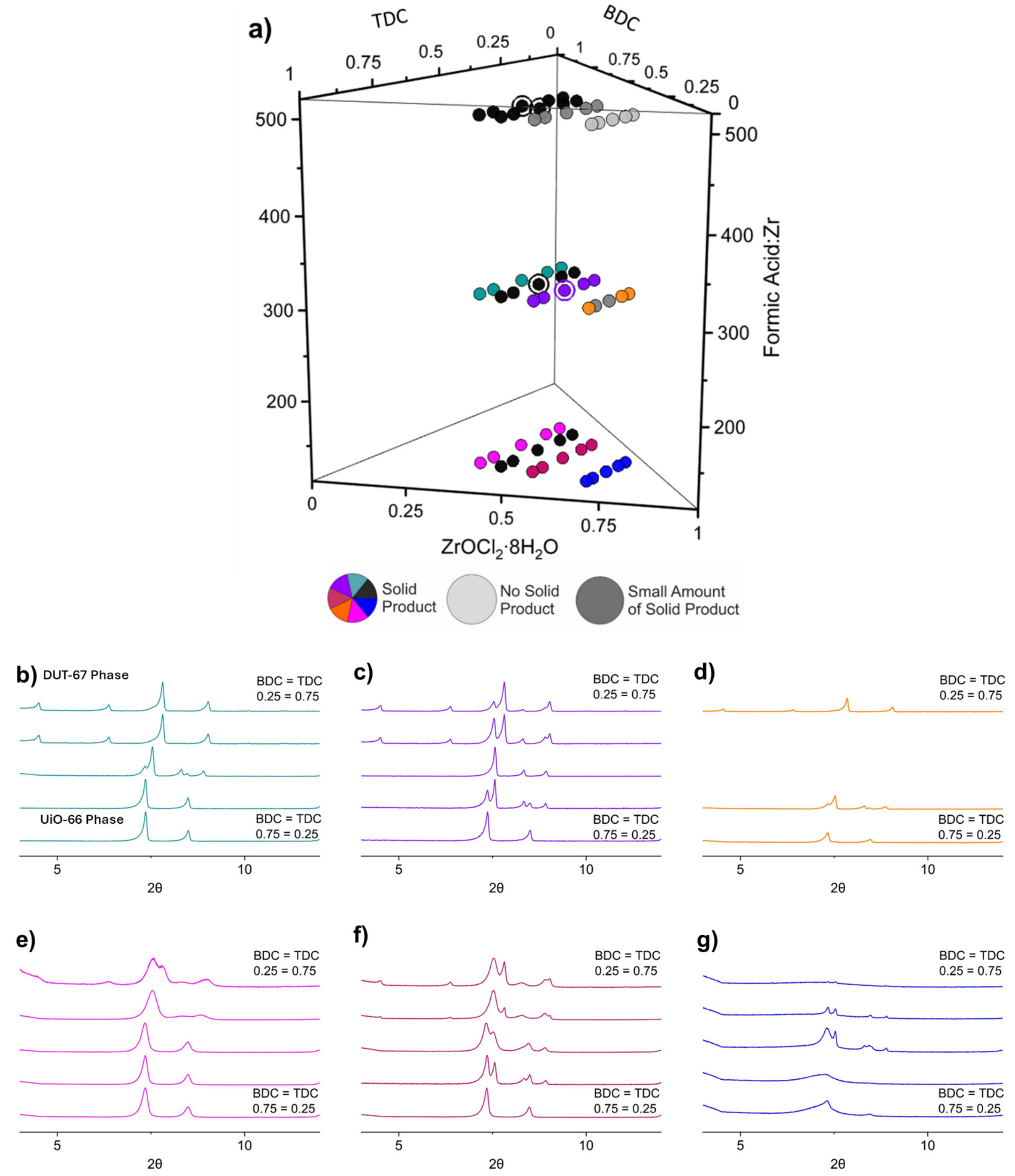

Figure S1 (a) Compositions of the 60 reaction mixtures selected for the initial batch synthesis to explore the system $ZrOCl_2$·$8H_2O$/BDC/TDC/FA/DMF. The light grey symbols identify reactions which lead to no solid product. The remaining symbols identify reactions which lead to a solid product, and are colour coded to correspond to the stack of PXRD patterns of the same colour in panels (b) – (e). PXRD patterns for the black points are given in Figure 1 of the main text. (b) – (e) contain the PXRD (Cu $K_{\alpha 1}$, λ = 1.5406 Å) patterns for the remaining 28 samples, identified by the black points in Figure 1. In each stack of PXRD patterns, the only variable is the BDC:TDC ratio. The patterns corresponding to the single linker phases (DUT-67 and UiO-66) are noted in (b).

Batch 2 Synthesis Tables and PXRD Data (Tables S3-S4 and Figure S2-S3)

Selection of compositions for the reactions of Batch 2

A set of 42 reactions were chosen to further elucidate the chemical space where the pure phase can be formed. This is not an exhaustive search of the space where the framework can be formed. Instead, it is merely to illustrate that the material can be made over a wide chemical space, in particular about the Zr: (BDC+TDC) and BDC:TDC ratios, compared to some multicomponent MOFs which can only be synthesised at restricted parameters.[1] The FA:Zr ratio was explored between 310 – 575, which cover the range of middle and high values of first batch, with five selected values (310, 375, 440, 500, and 575). No points were selected below FA:Zr = 310 because in the results of the first batch it was observed that $Zr_6(BDC)_4(TDC)_2$-DMF made at low FA:Zr ratio, 120, exhibit low crystallinity. For each FA:Zr ratio, the Zr:(BDC+TDC) molar ratio adopted three values, aside from the FA:Zr =440 layer which had two. The linker molar ratio, BDC:TDC, was explored with three unique values for each combination of Zr: (BDC+TDC) and FA:Zr because in the results of batch one the relative amount of the new phase compared to the known phases changes depending on the Zr:(BDC+TDC) and FA:Zr compositions. Therefore, in batch two the BDC:TDC ratios were chosen to be reflective of this and range from BDC:TDC=0.40:0.60 to BDC:TDC=0.60:0.40 (Table S4). The rest of the reaction conditions were consistent with the first batch. All 42 reactions yielded powder products.

Batch 2 Experimental

A stock solution of the Zr precursor was prepared by dissolving $ZrOCl_2 \bullet 8H_2O$ (5.63 mmol, 1.81 g) in DMF (250 mL). Linker stock solutions with eight different BDC:TDC molar ratios, BDC:TDC = 0.47:0.53, 0.5:0.5, 0.57:0.43, 0.45:0.55, 0.55:0.45, 0.43:0.57, 0.53:0.47 and 0.4:0.6, were prepared at a total linker (BDC+TDC) concentration of 0.15 M in DMF (25 mL). The quantities of BDC and TDC used in each solution are summarised in Table S3.

Table S3 Compositions, BDC:TDC, of stock solutions used in the second batch synthesis and the quantities of BDC and TDC used to prepare 0.15 M solutions of each in 25 mL of DMF.

BDC:TDC Molar Ratio	BDC		TDC	
	mmol	g	mmol	g
0.47:0.53	1.76	0.29	1.99	0.34
0.5:0.5	1.88	0.31	1.88	0.32
0.57:0.43	2.14	0.36	1.61	0.28
0.45:0.55	1.69	0.28	2.06	0.36
0.55:0.45	2.06	0.34	1.69	0.29
0.43:0.57	1.61	0.27	2.14	0.37
0.53:0.47	1.99	0.33	1.76	0.30
0.40:0.60	1.50	0.25	2.25	0.39

Table S4 Compositions of the 42 reaction mixtures from batch 2. For each composition, the molar quantities of the components used are given, along with the BDC:TDC molar ratio of the BDC:TDC linker stock solution used and the volume of each stock solution dispensed. Numbers are given to two decimal places, aside from the BDC (mmol) and TDC (mmol) data columns which are given to three decimal places. If phase pure = N, then a low intensity DUT-67 peaks are present alongside the new phase. Compositions that gave the pure phase (from PXRD) are highlighted in green. All of the points produced solid material. The PXRD data is plotted in Figure S2 below.

Zr:(BDC +TDC)	BDC:TDC (molar)	FA:Zr	$ZrOCl_2$ •$8H_2O$ (mmol)	BDC (mmol)	TDC (mmol)	$ZrOCl_2$ •$8H_2O$ Stock (mL)	BDC:TDC Stock (mL)	FA (mL)	DMF (mL)
0.50:0.50	0.47:0.53	310	0.12	0.056	0.064	5.33	0.80	1.40	2.47
0.50:0.50	0.50:0.50	310	0.12	0.060	0.060	5.33	0.80	1.40	2.47
0.50:0.50	0.57:0.43	310	0.12	0.068	0.052	5.33	0.80	1.40	2.47
0.50:0.50	0.47:0.53	375	0.12	0.056	0.064	5.33	0.80	1.70	2.17
0.50:0.50	0.50:0.50	375	0.12	0.060	0.060	5.33	0.80	1.70	2.17
0.50:0.50	0.57:0.43	375	0.12	0.068	0.052	5.33	0.80	1.70	2.17
0.44:0.56	0.45:0.55	310	0.12	0.068	0.083	5.33	1.00	1.40	2.27
0.44:0.56	0.50:0.50	310	0.12	0.075	0.075	5.33	1.00	1.40	2.27
0.44:0.56	0.55:0.45	310	0.12	0.083	0.068	5.33	1.00	1.40	2.27
0.44:0.56	0.45:0.55	375	0.12	0.068	0.083	5.33	1.00	1.70	1.97
0.44:0.56	0.50:0.50	375	0.12	0.075	0.075	5.33	1.00	1.70	1.97
0.44:0.56	0.55:0.45	375	0.12	0.083	0.068	5.33	1.00	1.70	1.97
0.40:0.60	0.43:0.57	310	0.12	0.077	0.103	5.33	1.20	1.40	2.07
0.40:0.60	0.50:0.50	310	0.12	0.090	0.090	5.33	1.20	1.40	2.07
0.40:0.60	0.53:0.47	310	0.12	0.095	0.085	5.33	1.20	1.40	2.07
0.40:0.60	0.43:0.57	375	0.12	0.077	0.103	5.33	1.20	1.70	1.77
0.40:0.60	0.50:0.50	375	0.12	0.090	0.090	5.33	1.20	1.70	1.77
0.40:0.60	0.53:0.47	375	0.12	0.095	0.085	5.33	1.20	1.70	1.77
0.40:0.60	0.47:0.53	440	0.12	0.085	0.095	5.33	1.20	1.99	1.48
0.40:0.60	0.50:0.50	440	0.12	0.090	0.090	5.33	1.20	1.99	1.48
0.40:0.60	0.57:0.43	440	0.12	0.103	0.077	5.33	1.20	1.99	1.48
0.40:0.60	0.47:0.53	500	0.12	0.085	0.095	5.33	1.20	2.26	1.21
0.40:0.60	0.50:0.50	500	0.12	0.090	0.090	5.33	1.20	2.26	1.21
0.40:0.60	0.57:0.43	500	0.12	0.103	0.077	5.33	1.20	2.26	1.21
0.40:0.60	0.47:0.53	575	0.12	0.085	0.095	5.33	1.20	2.60	0.87
0.40:0.60	0.50:0.50	575	0.12	0.090	0.090	5.33	1.20	2.60	0.87
0.40:0.60	0.57:0.43	575	0.12	0.103	0.077	5.33	1.20	2.60	0.87
0.33:0.67	0.43:0.57	440	0.12	0.103	0.137	5.33	1.60	1.99	1.08
0.33:0.67	0.50:0.50	440	0.12	0.120	0.120	5.33	1.60	1.99	1.08
0.33:0.67	0.53:0.47	440	0.12	0.127	0.113	5.33	1.60	1.99	1.08
0.33:0.67	0.43:0.57	500	0.12	0.103	0.137	5.33	1.60	2.26	0.81
0.33:0.67	0.50:0.50	500	0.12	0.120	0.120	5.33	1.60	2.26	0.81
0.33:0.67	0.53:0.47	500	0.12	0.127	0.113	5.33	1.60	2.26	0.81
0.33:0.67	0.43:0.57	575	0.12	0.103	0.137	5.33	1.60	2.60	0.47
0.33:0.67	0.50:0.50	575	0.12	0.120	0.120	5.33	1.60	2.60	0.47
0.33:0.67	0.53:0.47	575	0.12	0.127	0.113	5.33	1.60	2.60	0.47
0.29:0.71	0.4:0.60	500	0.12	0.120	0.180	5.33	2.00	2.26	0.41
0.29:0.71	0.45:0.55	500	0.12	0.135	0.165	5.33	2.00	2.26	0.41
0.29:0.71	0.50:0.50	500	0.12	0.150	0.150	5.33	2.00	2.26	0.41
0.29:0.71	0.40:0.60	575	0.12	0.120	0.180	5.33	2.00	2.60	0.07
0.29:0.71	0.45:0.55	575	0.12	0.135	0.165	5.33	2.00	2.60	0.07
0.29:0.71	0.50:0.50	575	0.12	0.150	0.150	5.33	2.00	2.60	0.07

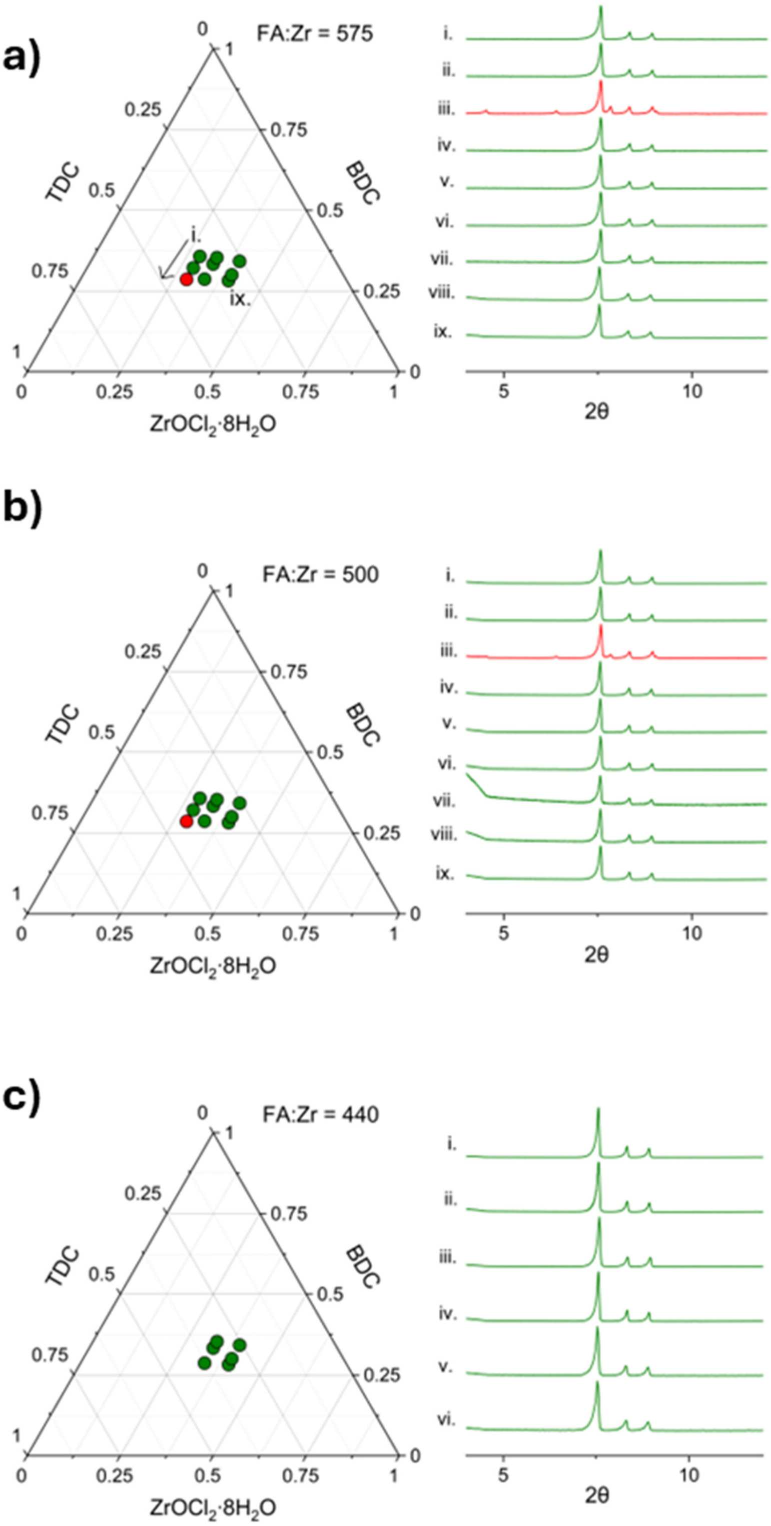

Figure S2 (a) (b) and (c) are ternary plots containing the batch 2 points alongside the corresponding PXRD data as measured with a Cu Bruker Diffractometer (as noted in roman numerals on the ternary plot of (a)). The green points represent the Batch 2 reactions that yielded the pure new phase, and the red points represent the reactions that yielded the new phase alongside a small impurity of a DUT-67 phase.

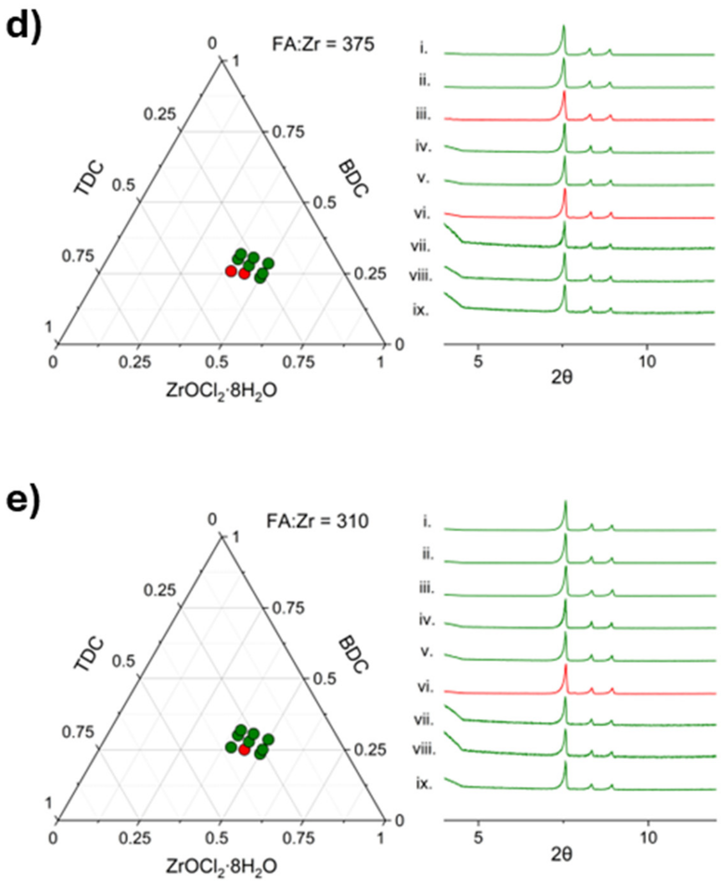

Figure S2 (d) and (e) are ternary plots containing the batch 2 points alongside the corresponding PXRD data as measured with a Cu Bruker Diffractometer. The green points represent the Batch 2 reactions that yielded the pure new phase, and the red points represent that reactions that yielded the new phase alongside a small impurity of a DUT-67 phase.

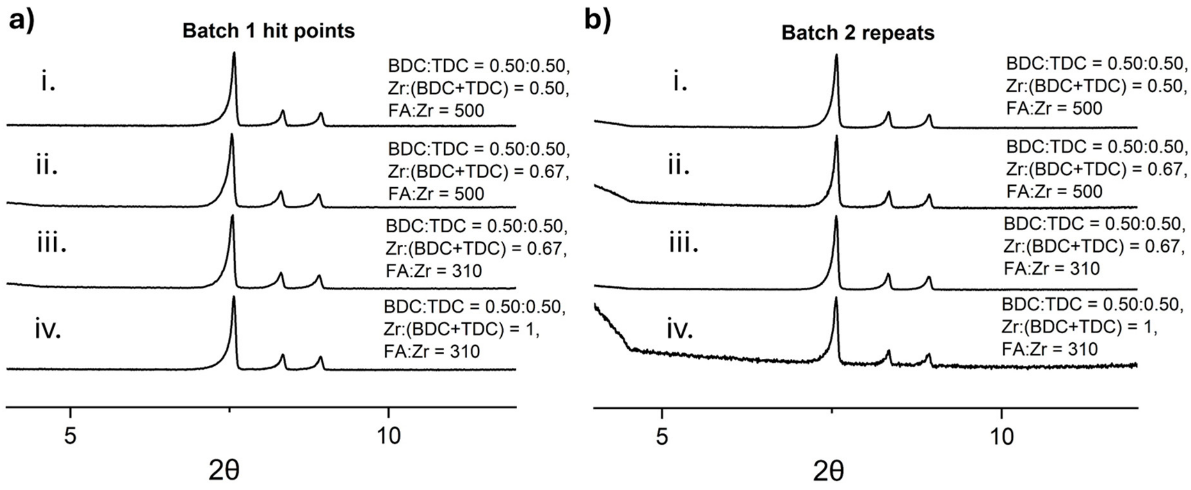

Figure S3 (a) i-iv. The in-house PXRD data of the hit points from Batch 1. (b) i-iv. The in-house PXRD data of the same points, however repeated in the batch 2 synthesis. These results show the reproducibility of the synthetic methodology.

^{1}H NMR Data of Batch 1 and 2 (Table S5 and Figure S4)

Table S5 Four reactions were chosen to be representative of the chemical space of the phase pure material to explore the linker incorporation in the resulting framework. This table provides the reaction mixture compositions: FA:Zr, Zr:(BDC+TDC), and BDC:TDC ratio of four reactions. The solids were washed with DMF 3 times over 24 hours, and methanol 3 times over 24 hours. The ^{1}H NMR spectra of the digested frameworks (Figure S4 a-d below) provided the 'NMR determined BDC:TDC' ratio; the area ratio of the peaks of BDC (7.73 ppm corresponds to the four aromatic protons) and TDC (7.35 to the two aromatic protons of TDC) is normalised to the number of protons. The results show the framework BDC:TDC ratio is dependent on the reaction mixture compositions. The 'NMR determined BDC:TDC' ranges from 0.64:0.36 to 0.53:0.47 BDC:TDC, when including the results from Figure S16 (^{1}H NMR of the sample which is the focus of the paper).

Zr:(BDC + TDC)	FA:Zr	Nominal BDC:TDC	NMR determined BDC:TDC	NMR spectrum
1	310	0.57:0.43	0.63:0.37	Figure S4 (a)
0.8	375	0.5:0.5	0.60:0.40	Figure S4 (b)
0.67	310	0.43:0.57	0.53:0.47	Figure S4 (c)
0.5	575	0.43:0.57	0.56:0.44	Figure S4 (d)

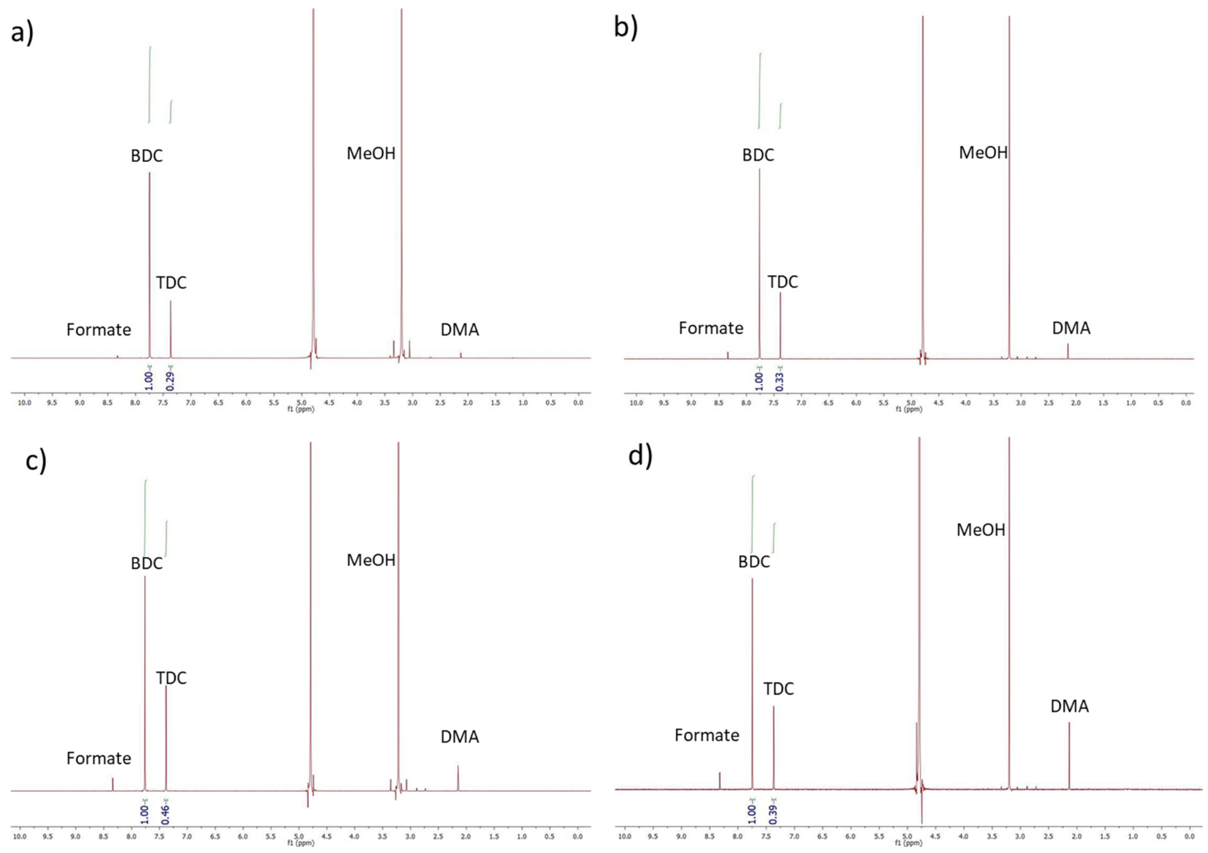

Figure S4 ^{1}H NMR spectra of the samples given in Table S5. The peak at 7.73 ppm is assigned to four aromatic protons of BDC, the peak at 7.4 ppm is assigned to two aromatic protons of TDC and the peak at 3.22 ppm is assigned to three methyl protons of MeOH. There are also residual formate (8.3 ppm), DMF (2.71 and 2.86 ppm), and dimethylamine (2.15 ppm) related peaks in the spectrum, this is as the solvent exchange to methanol was not fully successful. Dimethylamine and part of formate are derived from the decomposition of DMF during the digestion of the sample. The washing procedure for the chosen sample for further measurements, is given in the 'Solvent Exchange and Activation' section of the SI.

SEM image of the crystals (Figure S5)

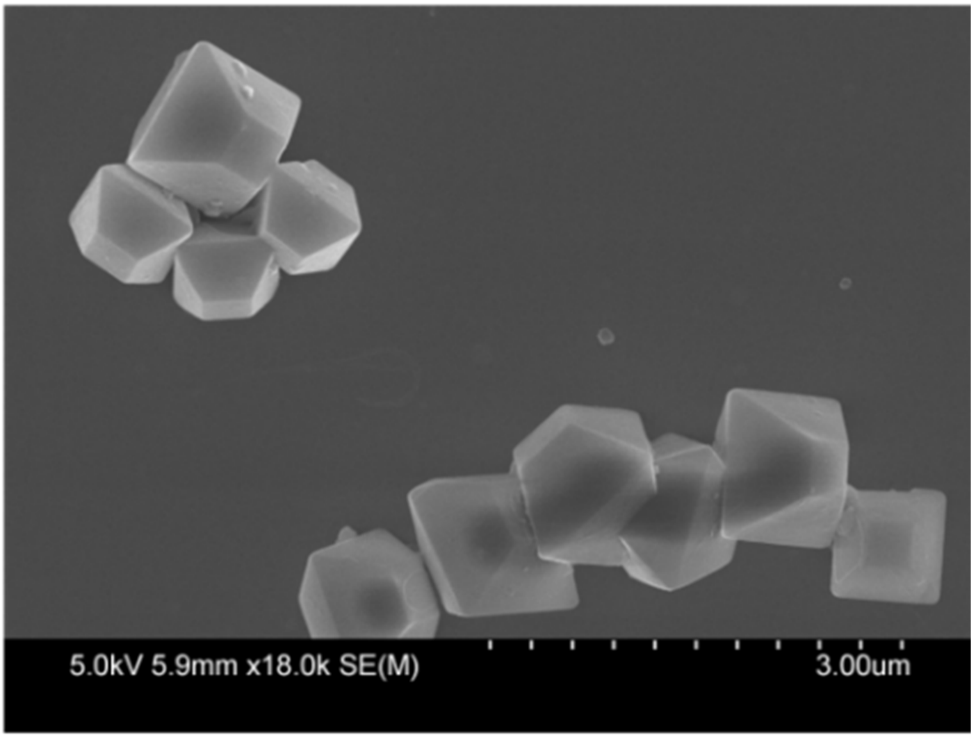

Figure S5 SEM image of $Zr_6(BDC)_4(TDC)_2$-H_2O showing the partially truncated octahedral crystallites. The scale bar on the image is 3.00 µm.

Structure Determination

$Zr_6(BDC)_4(TDC)_2$-DMF (Figures S6-S7)

Synchrotron PXRD was performed at Diamond Light Source U.K, on high-resolution beamline I11, with an incident beam wavelength of 0.825379(1) Å using the Mythen position-sensitive detector (PSD). The material in DMF (the solvent was exchanged three times to remove any unreacted starting material) is the focus for the overall structure discussion in the main text. PXRD data was indexed using the Singular Value Decomposition algorithm[15] as implemented in TOPAS-Academic V5[16] to the tetragonal unit cell a = 19.8708 Å, c = 42.4808 Å and V = 16773.493 Å^3 (GOF = 61.14). The space group was assigned based on the observed systematic absences which were consistent with tetragonal space group, $I4_1/acd$ (space group no. 142). Pawley refinement[17] was performed to confirm that the suggested combination of unit cell and space group provides the best fit, as well as to model the background using a 24-term Chebyshev polynomial function and describe the peak profile with a Pseudo-Voigt function. The parameters of the tetragonal unit cell were determined from the Pawley refinement as a = 19.88064(8) Å and c = 42.5000(2) Å and V = 16797.6(2) Å^3. The structural model was constructed based on the analogy of the determined unit cell to a double unit cell of UiO-66, a cubic crystal structure with a = 20.7004 Å. [18, 19] The positions of the $Zr_6O_4(OH)_4(COO)_{12}$ clusters (Figure S6a) are analogous to the cluster positions in a double UiO-66 unit cell and the centre of the clusters corresponds to Wyckoff sites 8a of the $I4_1/acd$ space group. These positions were confirmed by simulated annealing of 8 clusters in a unit cell with the same dimensions and $P1$ symmetry (Figure S6b).[20]

Each cluster has four adjacent coplanar clusters at a distance 14.058 Å and eight others, four above and four below at distance 14.550 Å (Figure S6c). These distances are similar to those observed in DUT-67 and UiO-66 which are 13.827 Å and 14.668 Å respectively (distances measured from the two centroids of two Zr_6 clusters). The locations of the linkers in the new phase were placed between the pair of clusters based on the distances corresponding to the single linker MOFs, BDC is placed in the larger distance and TDC in the shorter one. There are two independent crystallographic sites for BDC, BDC1 and BDC2 centring on the Wyckoff sites 16c and 16e respectively. TDC is centred on the Wyckoff site 16f. The crystallographically independent portion of the Zr_6O_8 cluster and of the BDC and TDC linkers were modelled by rigid bodies through the z-matrix formalism with dummy atoms corresponding to the centres of the Zr cluster and linker molecules. The structure refinement was then carried out with the Rietveld method[21] as implemented in TOPAS-Academic V5. For each rigid body, dummy atoms fixed at the corresponding Wyckoff sites, the atomic coordinates were allowed to refine by translation and rotation about the crystallographic axes that would not affect their molecular shape and the multiplicity of their atoms. Particularly, for the Zr cluster the atomic coordinates were refined by rotation about the c-axis; for BDC1, by rotation about each crystallographic axis; for BDC2, by both translation on and rotation about the b-axis; and for TDC, by translation on the a- and b-axes under the restriction of $x + y = 0.5$. The occupancy of TDC was fixed at 0.98 and the occupancies of BDC1 and BDC2 were refined in the range 0.78 to 1 having their sum fixed at 1.78 in order to be consistent with the composition of the sample $Zr_6O_4(OH)_4(BDC)_{3.56}(TDC)_{1.96}(HCOO)_{0.96}\cdot 6.99DMF$ as determined from the digestion NMR of a solid sample combined with TGA results (see section Chemical formula of Bulk

Samples). Bond distances and angles between atoms fixed initially in the rigid bodies at values observed in UiO-66 and DUT-67 crystal structures (RUBRAK and XICNOO respectively on the CCDC database) were allowed to refine within constraints at the final stages of the Rietveld refinement. Simulated annealing was also used to find initial locations and orientations of three DMF molecules in the pores of the framework. Each DMF was described as rigid body, and their location and orientation were allowed to refine at the final stages of the Rietveld refinement. Finally, atomic displacement parameters were refined anisotropically for each element in Zr cluster to have a single value and for all atoms to have the same value in each linker rigid body. The final Rietveld refinement is shown in Figure S7, and refinement parameters are listed in Table S6.

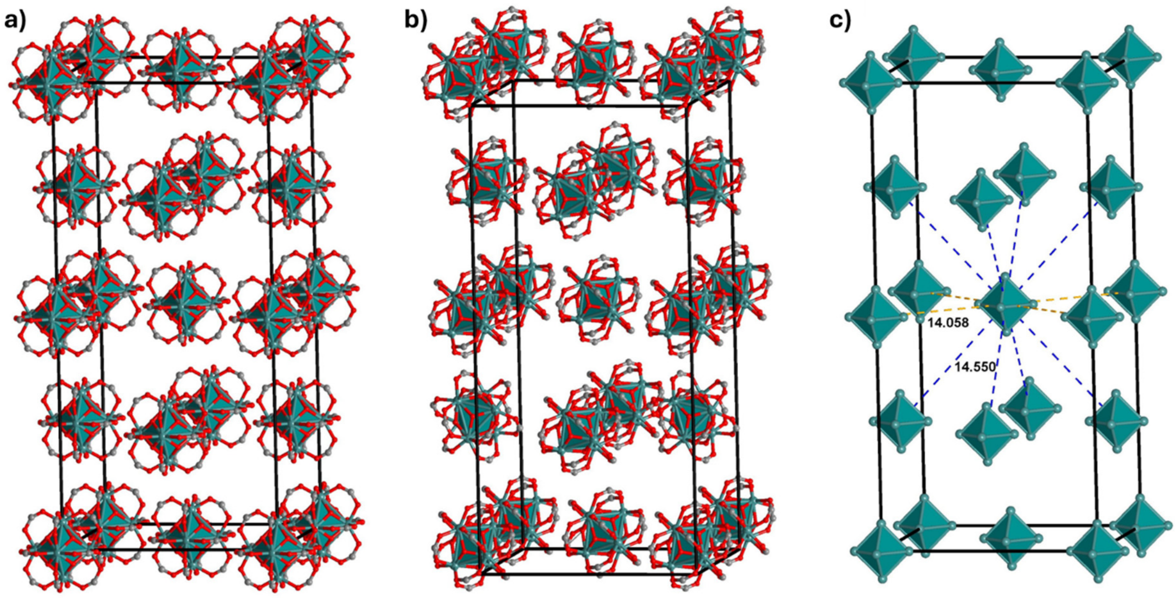

Figure S6 (a) $Zr_6O_4(OH)_4(COO)_{12}$ clusters in the unit cell of $Zr_6(BDC)_4(TDC)_2$-DMF at sites analogous to the cluster sites of UiO-66 structure in a doubled unit cell. (b) Sites of 8 independent $Zr_6O_4(OH)_4(COO)_{12}$ clusters in *P*1 unit cell obtained from simulated annealing of the PXRD pattern of $Zr_6(BDC)_4(TDC)_2$-DMF. (c) Distances between clusters in the unit cell of $Zr_6(BDC)_4(TDC)_2$-DMF. Each cluster has four adjacent coplanar clusters at a distance 14.058 Å and eight others, four above and four below at distance 14.550 Å.

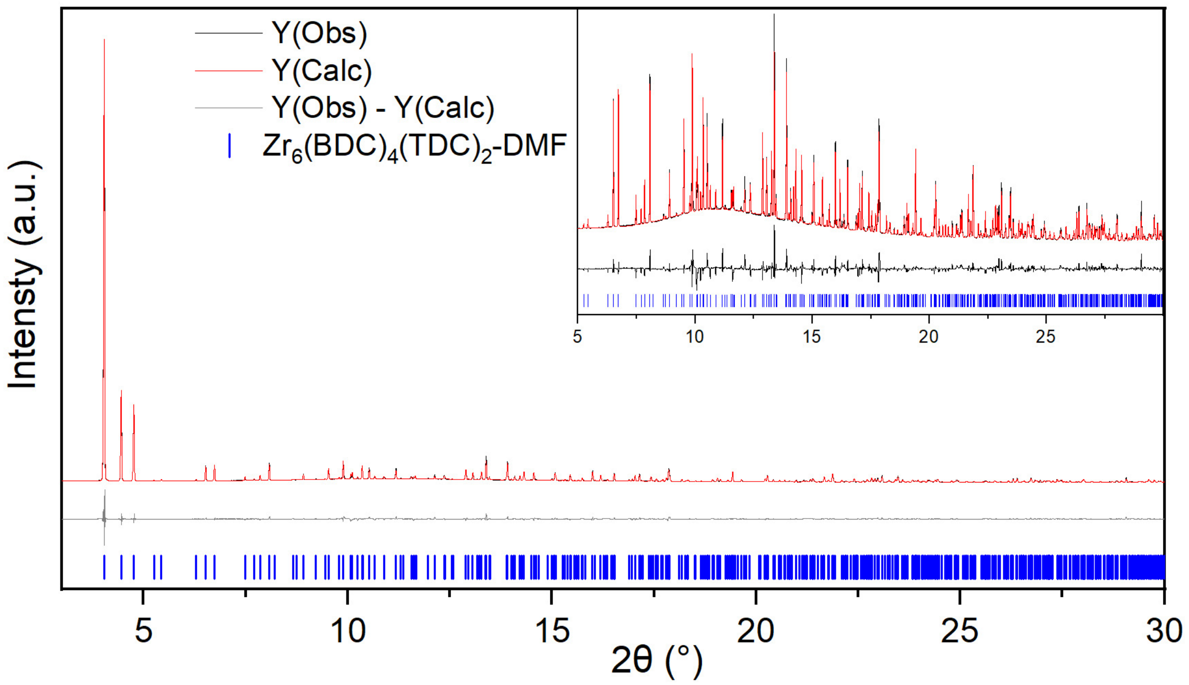

Figure S7 Rietveld analysis of the PXRD data measured for $Zr_6(BDC)_4(TDC)_2$-DMF.

$Zr_6(BDC)_4(TDC)_2$-MeOH (Figure S8)

The PXRD pattern of the $Zr_6(BDC)_4(TDC)_2$-MeOH was indexed using the Singular Value Decomposition algorithm as implemented in TOPAS-Academic V5 to the tetragonal unit cell *a* = 19.9025 Å, *c* = 42.6402 Å and V = 16890.236 Å^3 (GOF = 91.72). The space group was assigned based on the observed systematic absences which were consistent with tetragonal space group, $I4_1/acd$ (space group no. 142). Pawley refinement was performed to confirm the unit cell and the space group and to model the background with a 24 term Chebyshev polynomial function and the peak profile based on Pseudo-Voigt function. The parameters of the tetragonal unit cell determined from the Pawley refinement as *a* = 19.9137(1) Å and *c* = 42.6611(3) Å and V = 16917.5(3) Å^3. Rietveld refinement attempts on the pattern using the Zr cluster and linker positions from the structure models of $Zr_6(BDC)_4(TDC)_2$-DMF did not provide a satisfactory fitting. Instead, the refinement on a model that was based on $Zr_6(BDC)_4(TDC)_2$-H_2O structure, obtained by electron diffraction (see below), was successful. In this model, TDC does not link coplanar Zr clusters through coordination bonds, but it is positioned between the planes of Zr clusters. BDC and Zr clusters occupy the same sites as in $Zr_6(BDC)_4(TDC)_2$-DMF. The model of $Zr_6(BDC)_4(TDC)_2$-MeOH was completed by adding MeOH as capping ligand on the sites of Zr clusters previously occupied by TDC carboxylates and MeO^-/MeOH capping pairs on the sites of missing linkers, which were occupied by formate anions in the $Zr_6(BDC)_4(TDC)_2$-DMF structure. Adsorbed MeOH and H_2O for the electron density observed in the pores. Simulated annealing was used to find initial locations and orientations of these species. The formula of the refined model, $Zr_6O_4(OH)_4(BDC)_{3.56}(TDC)_{1.96}(MeO^-)_{0.96}(MeOH)_{8.8}\cdot1.1MeOH\cdot8H_2O$, was determined by the combination of ^{1}H NMR of the digested sample and TGA (See SI section Chemical formula of Bulk Samples). The structure refinement was then carried out with the Rietveld method as implemented in TOPAS-Academic V5 and following the same procedure as in the Rietveld refinement of $Zr_6(BDC)_4(TDC)_2$-DMF (Figure S8). The crystallographic data table for the structure refinement against powder diffraction data of $Zr_6(BDC)_4(TDC)_2$-MeOH is provided in Table S7.

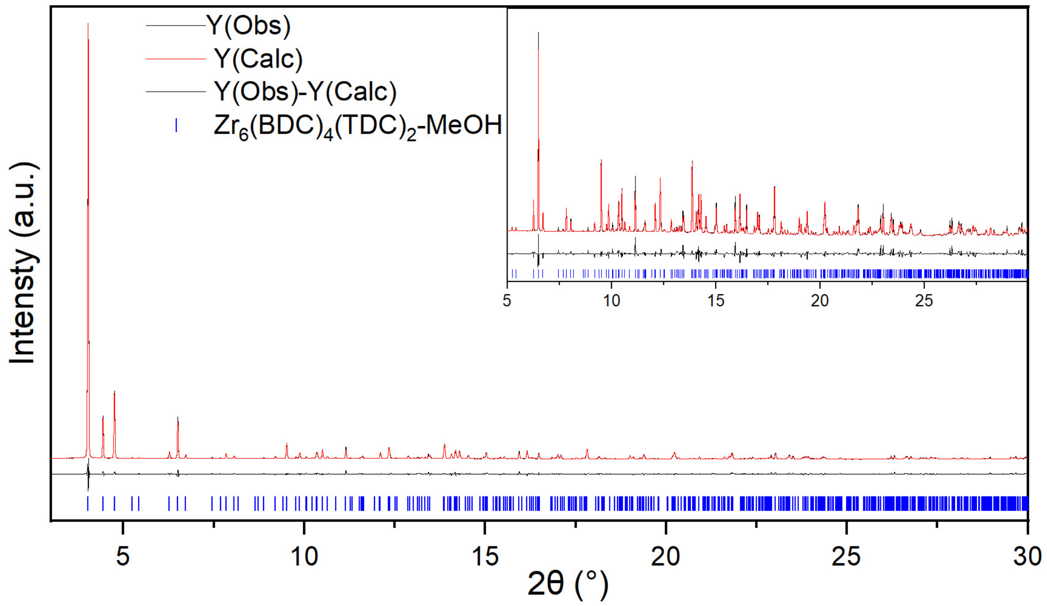

Figure S8 Rietveld analysis of the PXRD data measured for $Zr_6(BDC)_4(TDC)_2$-MeOH.

3D Electron Diffraction of $Zr_6(BDC)_4(TDC)_2$-H_2O (Figure S9)

The sample $Zr_6(BDC)_4(TDC)_2$-H_2O was a colourless, crystalline solid. The sample consisted of plate-like crystallites of a few 100 nanometres in thickness. The dataset was measured with a wavelength of 0.0251 Å at 100 K. Collecting data at low temperature helped to reduce beam damage, stabilize the sample in vacuo, and especially improve the resolution. As 3D ED requires samples to be studied in vacuo, cryo-transfer technique is essential for sensitive compounds like solvent containing MOFs. With the help of a cryo-transfer-station, crystals were ultimately flash-cooled and measured at 100 K under high vacuum improving the diffraction limit up to 1.10 Å (diffracted only to ca. 1.9 Å at RT and without applying cryo-transfer). The single measurement lasted less than 2 minutes (tilt range: -50° to +50°, scan width: 0.20°, exposure time: 0.20 s, total dose: 0.12 e-/(Å^2)). The structure was solved and refined with kinematical approximation using the standard ShelX[6, 7] programs in the latest version of Olex2.[8] Accurate structure factors for electron beams are automatically added to the *.ins file for refinement. Refinement results of electron diffraction data are affected by dynamical diffraction and the special nature of charged particles. Due to these effects, R-values are systematically higher than for single crystal X-ray diffraction. However, within the context of electron diffraction data, not taking these effects into account yet, the results are satisfactory. The three-dimensional metal-organic framework of $Zr_6(BDC)_4(TDC)_2$-H_2O was refined with the help of restraints (DFIX, RIGU, SIMU, FLAT, and ISOR).[22, 23] The weight factors were left intentionally at 0.1 and 0, respectively, giving the GOF = 2.330, as the refinement was conducted with kinematical approximation. As the structure was solved and refined with tools (kinematical approximation) developed for X-ray diffraction, and we are dealing with 3DED data, full dynamical computations were not considered for refinement as it is computationally expensive and only used for absolute structure determination. A PLATON Squeeze analysis was performed to remove the remaining residual electron density from the pores. The calculation gave a 6192 Å^3 guest accessible volume, removing 484 electrons from the Fourier difference map equating to 48 H_2O molecules per unit cell in the pores. No further restraints were applied during the refinement of the structure and all non-H atoms were refined anisotropically. All H-atoms in the structure were placed in idealized positions and included as riding. The crystallographic data and structure refinement details of $Zr_6(BDC)_4(TDC)_2$-H_2O are in Table S8 below. CCDC 2425621 contains the supplementary crystallographic data for this publication. The PXRD pattern of $Zr_6(BDC)_4(TDC)_2$-H_2O is provided in the main text in Figure 7a.iii.

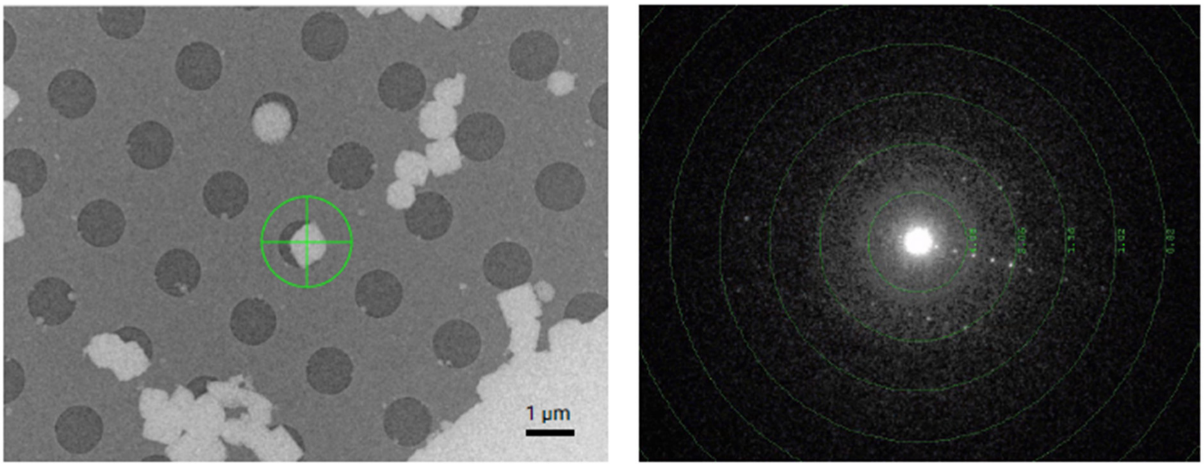

Figure S9 (Left) Transmission electron microscope image of the studied grain, and (right) exemplary diffraction pattern.

$Zr_6(BDC)_4(TDC)_2$ (Figures S10-11)

The PXRD pattern of the $Zr_6(BDC)_4(TDC)_2$ was indexed using the Singular Value Decomposition algorithm as implemented in TOPAS-Academic V5 to the tetragonal unit cell a = 20.0101 Å, c = 42.1392 Å and V = 16872.664 Å^3 (GOF = 103.68). The space group was assigned based on the observed systematic absences which were consistent with tetragonal space group, $I4_1/acd$ (space group no. 142). Pawley refinement was performed to confirm the unit cell and the space group and to model the background with a 24 term Chebyshev polynomial function and the peak profile based on Pseudo-Voigt function. The parameters of the tetragonal unit cell determined from the Pawley refinement as a = 20.02145(8) Å and c = 42.1572(2) Å and V = 16899.1(2) Å^3. Rietveld refinements on the pattern using the Zr cluster and linker positions from the structure models of $Zr_6(BDC)_4(TDC)_2$-DMF and $Zr_6(BDC)_4(TDC)_2$-H_2O were attempted. The solution demonstrated that $Zr_6(BDC)_4(TDC)_2$ has the same arrangement of Zr clusters and linkers as $Zr_6(BDC)_4(TDC)_2$-DMF with TDC linking clusters through Zr-O coordination bonds. The location of TDC was also confirmed by the inspection of the electron density Fourier Map, F(obs) – F(calc), using structure model only with Zr clusters and BDC linkers (Figure S11). The residual electron density is located on the (004) planes of the unit cell and has the shape of TDC. Having confirmed the arrangement of clusters and linkers, the Rietveld refinement of $Zr_6(BDC)_4(TDC)_2$ was progressed in the same way as for $Zr_6(BDC)_4(TDC)_2$-DMF refining the coordinates and orientation of each rigid body and the distances and angles between atoms in each rigid body at the final stages of the refinement. The occupancies of the linkers were fixed to satisfy the experimentally determined formula of the sample $Zr_6O_4(OH)_4(BDC)_{3.48}(TDC)_{1.93}(OH)_{1.18}$. The atomic displacement parameters were refined anisotropically by restraining the parameters to a single value for each atom type in the Zr cluster and a single value for all atoms in each linker. The crystallographic data table for the structure refinement against powder diffraction data of $Zr_6(BDC)_4(TDC)_2$ is provided in Table S9.

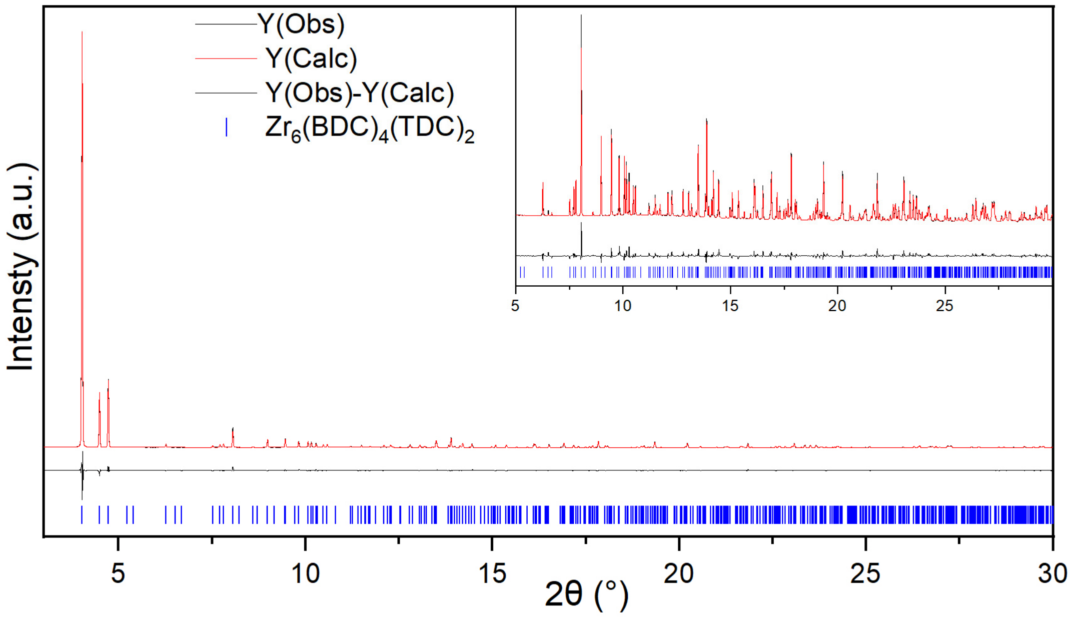

Figure S10 Rietveld analysis of the PXRD data measured for $Zr_6(BDC)_4(TDC)_2$.

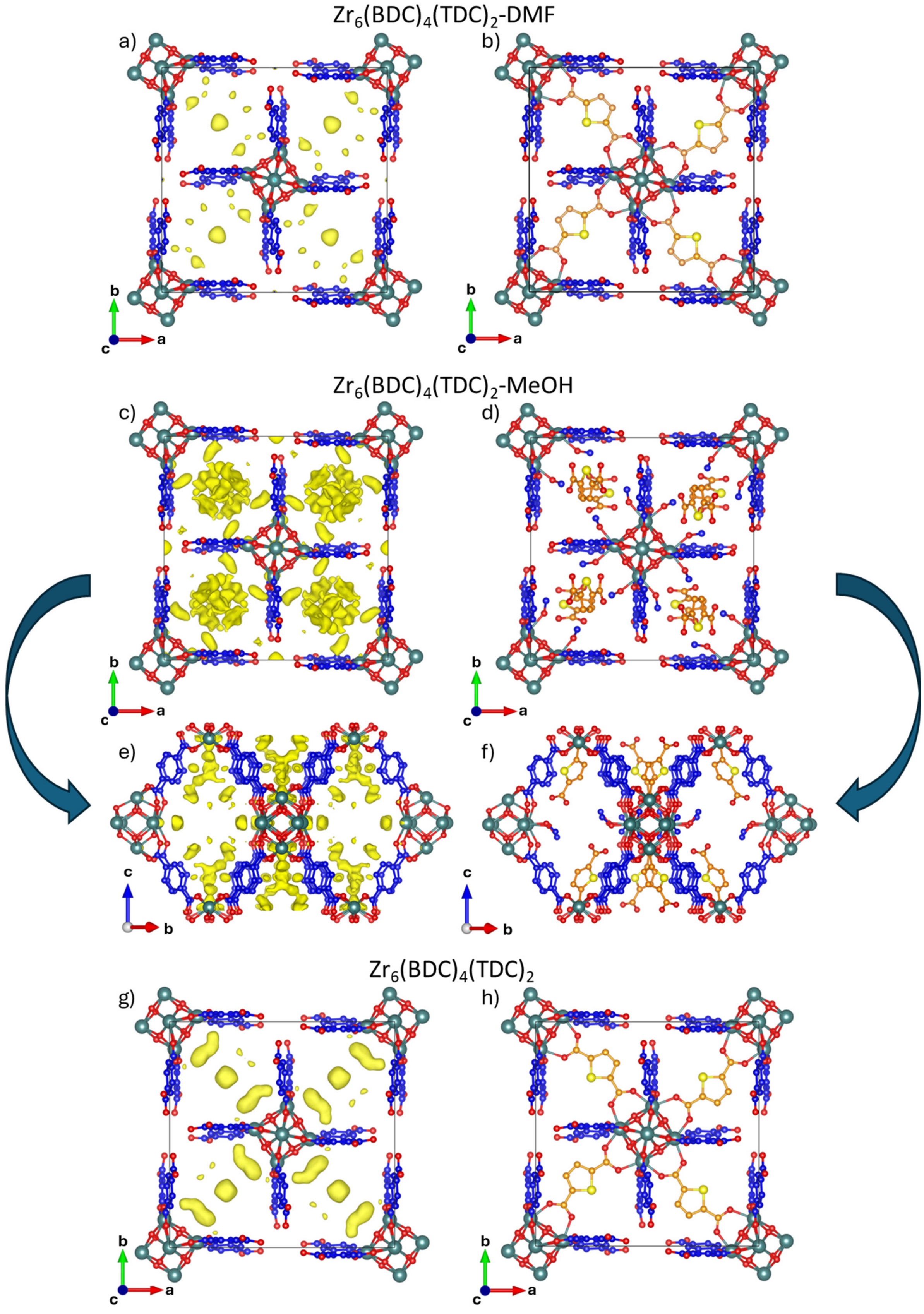
Zr$_6$(BDC)$_4$(TDC)$_2$-DMF
a)
b)
Zr$_6$(BDC)$_4$(TDC)$_2$-MeOH
c)
d)
e)
f)
Zr$_6$(BDC)$_4$(TDC)$_2$
g)
h)
b
a
c

Figure S11 The electron density Fourier Maps, F(obs) – F(calc), using the structure model of each compound containing only with Zr_6 clusters and BDC linkers, demonstrate the electron density associated with TDC in each structure as observed by PXRD measurements and are compared with the location of TDC in each refined crystal structure. (a) and (b) The Fourier map and the structure representation of $Zr_6(BDC)_4(TDC)_2$-DMF viewed down the *c*-axis show TDC connecting Zr_6 clusters on a plane perpendicular to *c*-axis. (c) and (d) The Fourier map and the structure representation of $Zr_6(BDC)_4(TDC)_2$-MeOH viewed down the *c*-axis show that TDC is not lying on a plane perpendicular to *c*-axis. (e) and (f) The Fourier map and the structure representation of $Zr_6(BDC)_4(TDC)_2$-MeOH viewed perpendicular to the *c*-axis show that TDC is located in the space between the planes of Zr_6 clusters. (g) and (h) The Fourier map and the structure representation of $Zr_6(BDC)_4(TDC)_2$ viewed down the *c*-axis show TDC connecting Zr_6 clusters on a plane perpendicular to *c*-axis. In all representations the residual electron density is shown yellow, the Zr atoms in teal, oxygen atoms in red, and the carbons of the BDC linker in blue. The structure representations contain also TDC with the carbons in orange and the sulfur atom in yellow for emphasis.

Crystallographic Data Tables (Tables S6 –S9)

Table S6 Structure refinement against powder diffraction data of $Zr_6(BDC)_4(TDC)_2$-DMF.

	**$Zr_6(BDC)_4(TDC)_2$-DMF**
**Empirical Formula**	$C_{30.95}H_{35.23}O_{19.45}S_{0.98}N_{3.45}Zr_3$
**Formula Weight (g mol^{-1})**	1072.02
**Space Group**	$I4_1/acd$(n. 142)
**Z**	16
**Density (g cm^{-3})**	1.696
**Temperature (K)**	298.15
**Wavelength (Å)**	0.826596 (10)
**d – spacing range (Å)**	0.57307 – 14.78028
**Number of reflections**	11848
**Number of refined parameters**	88
***a* (Å)**	19.88173(5)
***c* (Å)**	42.49860(13)
**Volume (Å^3)**	16798.99(10)
**R_p**	3.21
**R_{wp}**	4.85
**R_{exp}**	0.92
**GOF**	5.29
**CCDC**	2440844

Table S7 Structure refinement against powder diffraction data of $Zr_6(BDC)_4(TDC)_2$-MeOH.

	$Zr_6(BDC)_4(TDC)_2$-MeOH
**Empirical Formula**	$C_{24.67}H_{37.28}O_{24.47}S_{0.98}Zr_3$
**Formula Weight (g mol^{-1})**	1030.46
**Space Group**	$I4_1/acd$ (n. 142)
**Z**	16
**Density (g cm^{-3})**	1.618
**Temperature (K)**	298.15
**Wavelength (Å)**	0.826596 (10)
**d – spacing range (Å)**	0.57307 – 15.76538
**Number of reflections**	11935
**Number of refined parameters**	92
***a* (Å)**	19.91546(12)
***c* (Å)**	42.6593(4)
**Volume (Å^3)**	16919.8(3)
**R_p**	6.02
**R_{wp}**	9.33
**R_{exp}**	1.16
**GOF**	8.05
**CCDC**	2440845

Table S8 Crystallographic data and structure refinement details of $Zr_6(BDC)_4(TDC)_2$-H_2O.

	**$Zr_6(BDC)_4(TDC)_2$-H_2O**
**Empirical Formula**	$C_{22}H_{26}O_{23}SZr_3$
**Formula Weight (g mol^{-1})**	910.10
**Crystal Description**	Colourless plate
**Space Group**	$I4_1/acd$ (no. 142)
a **(Å)**	19.94339(15)
c **(Å)**	42.51118(19)
**Volume (Å^3)**	16908.3(3)
**Z**	16
**Z'**	0.5
**Density (g cm^{-3})**	1.430
**Temperature (K)**	100
**Wavelength (Å)**	0.0251
**Completeness, %**	98.8
**Resolution, Å**	1.35
**Number of reflections (observed)**	6931(471)
**Number of refined parameters (data)**	212(892)
**R_{int}, %**	0.3201
**R_1, %, wR_2, %, GoF for $F^2>2s(F^2)$**	0.1945, 0.4488, 2.330
**Largest diff. peak/hole /e Å^{-3}**	0.365, -0.430
**CCDC**	2425621

Table S9 Structure refinement against powder diffraction data of $Zr_6(BDC)_4(TDC)_2$.

	**$Zr_6(BDC)_4(TDC)_2$**
**Empirical Formula**	$C_{19.71}H_{10.89}O_{15.41}S_{0.97}Zr_3$
**Formula Weight (g mol^{-1})**	798.85
**Space Group**	$I4_1/acd$ (n. 142)
**Z**	16
**Density (g cm^{-3})**	1.256
**Temperature (K)**	298.15
**Wavelength (Å)**	0.825005
**d – spacing range (Å)**	0.57281 – 17.50877
**Number of reflections**	11940
**Number of refined parameters**	87
***a* (Å)**	20.02237(5)
***c* (Å)**	42.15947(13)
**Volume (Å^3)**	16901.54(10)
**R_p**	3.86
**R_{wp}**	5.340
**R_{exp}**	0.42
**GOF**	12.78
**CCDC**	2440846

Further Details of the Crystal Structures (Figures S12 – S15)

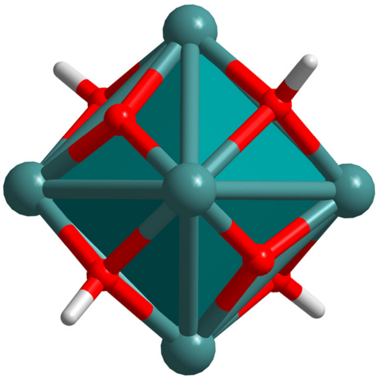

Figure S12 The $Zr_6O_4(OH)_4(COO)_{12}$ cluster of the $Zr_6(BDC)_4(TDC)_2$-DMF, with the hydroxides and oxides shown. All the eight faces of the octahedron are isosceles triangles. Four of the triangular faces have a μ_3-O^{2-} located above them, and the remaining four μ_3-OH^- ligands are located above the other four faces, as typical for $[Zr_6O_4(OH)_4]^{12+}$ clusters. The Zr1-Zr2 edge is 3.4914(11) Å and is occupied by the carboxylates of BDC, and the Zr2-Zr2 one is 3.5087(14) Å and is occupied by the carboxylates of TDC. Zr atoms are teal, oxygen atoms are red, hydrogen atoms are light grey, the carboxylate carbon of BDC is blue, and the carboxylate carbon of TDC is orange.

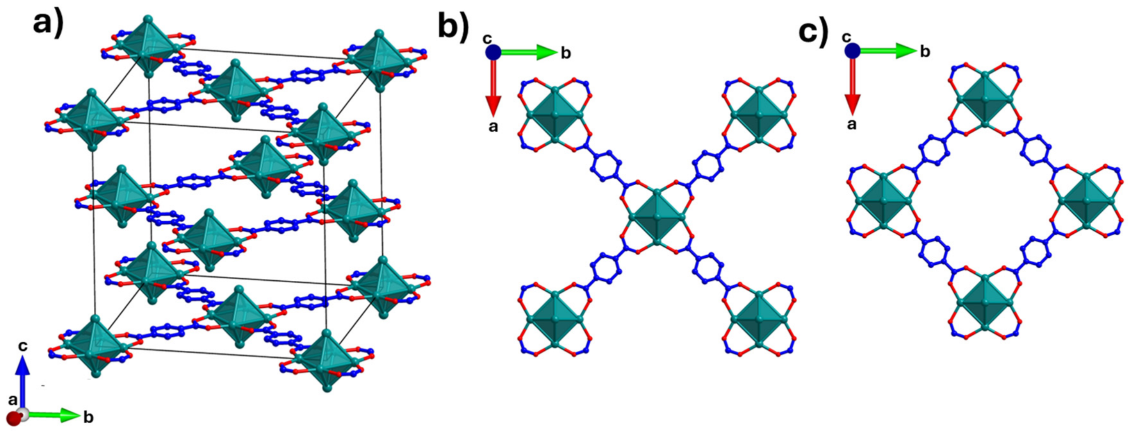

Figure S13 (a) UiO-66 depicted with Zr_6 octahedra and BDC linkers.[18] (b) and (c) are the BDC layers, with (b) corresponding to the layer at the top of the unit cell. This is to highlight that the incorporation of the bent TDC in $Zr_6(BDC)_4(TDC)_2$-DMF results in the doubling of the *c*-axis compared to UiO-66 owing to the two orientations of the TDC around the Zr_6 cluster, as shown in Figure 4.

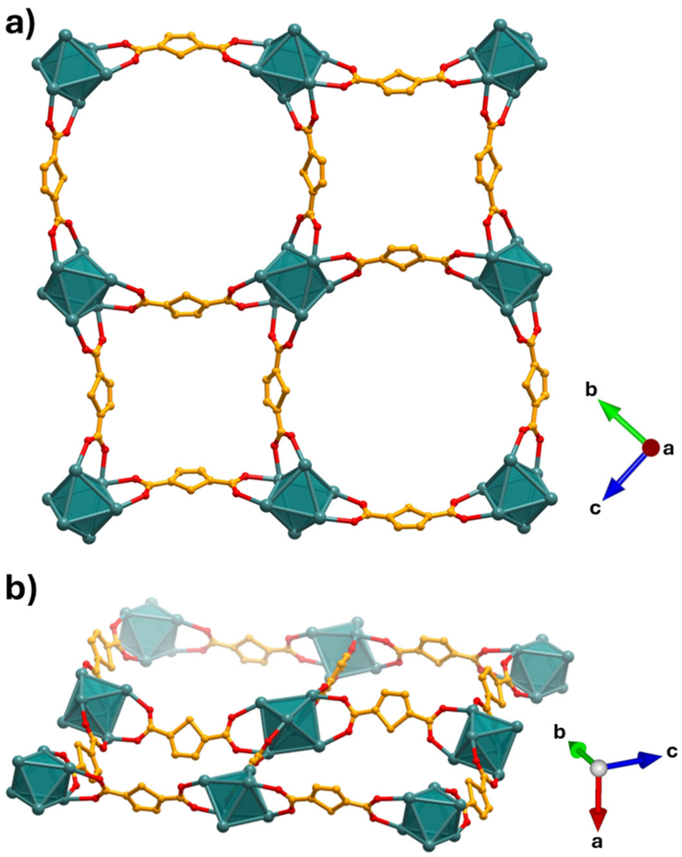

Figure S14 (a) $Zr_6(TDC)_2$ layer in DUT-67.[24] The layer consists of two types of rings formed by the arrangement of Zr_6 octahedra and TDC, in contrast to the $Zr_6(TDC)_2$ layer in $Zr_6(BDC)_4(TDC)_2$-DMF which has one ring. (b) The same layer from a different viewpoint. Notably, the Zr_6 clusters are rotated in all axes, and thus the TDC linkers and Zr_6 nodes are not coplanar, this can be directly compared to Figure 5a in the main text, which depicts the $Zr_6(TDC)_2$ layer of $Zr_6(BDC)_4(TDC)_2$-DMF.

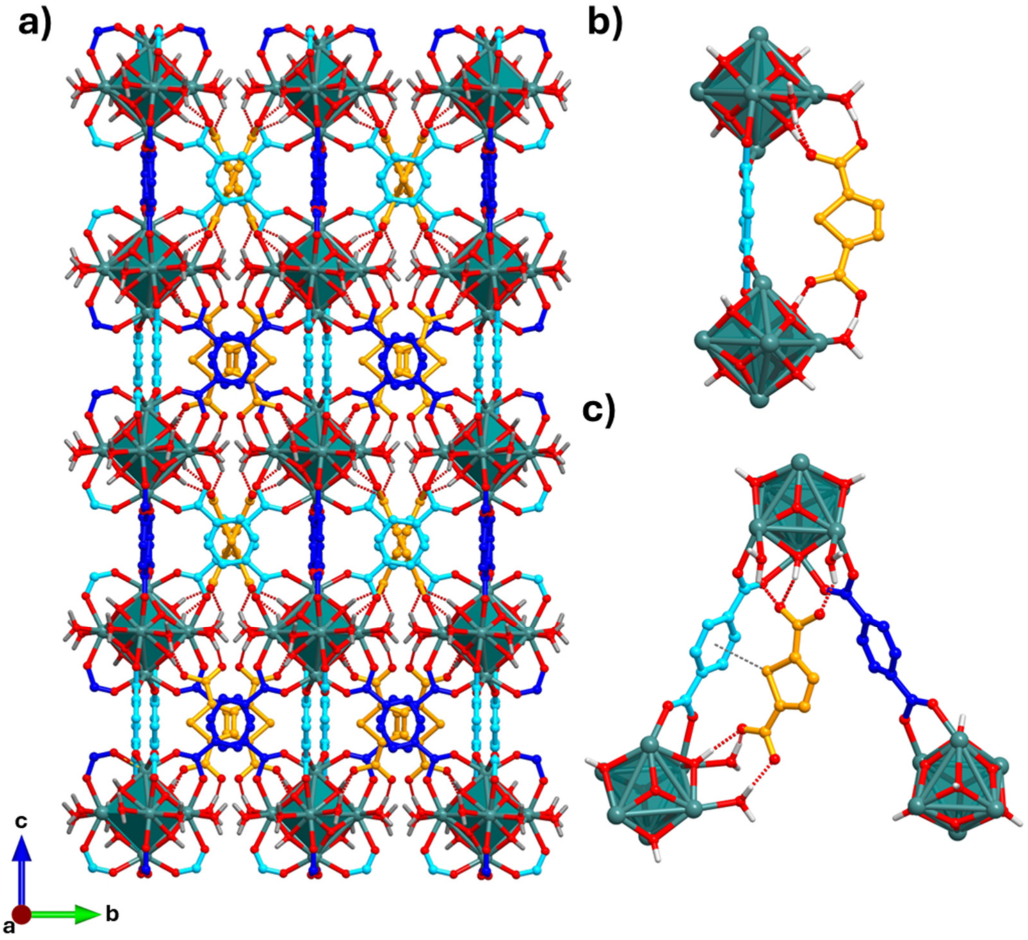

Figure S15 (a) The unit cell of $Zr_6(BDC)_4(TDC)_2$-H_2O crystal structure. This is similar to the $Zr_6(BDC)_4(TDC)_2$-MeOH structure as provided in the main text (Figure 6), however with coordinated water molecules on the Zr_6 octahedra rather than methanol (b) The TDC linker is connected through hydrogen bonds to hydroxides and coordinated water on two Zr_6 clusters that are linked by BDC site 2. (c) The position of the TDC relative to BDC site 1 and BDC site 2. An interaction of the S atom of TDC with the aromatic ring of BDC site 2 is shown with a dashed grey line for emphasis. For (b) and (c) only the water molecules involved in the TDC H-bonding are shown. For all depictions, the Zr_6 nodes are shown as teal octahedra with the oxides and hydroxides included, to show their interaction with the TDC. The water coordinated to the Zr_6 clusters is shown. The C atoms of BDC site 1 in blue and cyan for BDC site 2, and the TDC linkers have orange C and S atoms. The oxygen atoms are red. Hydrogens are light grey. The red dotted lines represent hydrogen bonds between the TDC, and the solvent coordinated to the cluster and the cluster hydroxides. The solvent in the pores and the H atoms of both the linkers' carbon atoms are omitted for clarity.

Chemical Formula of Bulk Samples (Figures S16 – S19)

The chemical formula of bulk samples was derived from the combination of TGA and ^{1}H NMR measurements, which provided the relative amounts of Zr cluster, BDC and TDC linkers in each sample. The measurements performed on $Zr_6(BDC)_4(TDC)_2$-MeOH that had been washed with DMF and MeOH to remove unreacted chemicals from the surface and the pores of the material. The results of these measurements were worked backwards to obtain the formula of the as-made $Zr_6(BDC)_4(TDC)_2$-DMF. The same analyses have been also performed on the activated sample $Zr_6(BDC)_4(TDC)_2$.

The ratio between BDC and TDC linkers in each sample was determined by ^{1}H NMR of the digested samples in a mixture of NaOD (60 µL) and D_2O (640 µL). The molar ratio of the two linkers was derived from the relative areas of the aromatic proton peaks normalised to the number of protons per molecule.

The ^{1}H NMR spectra of $Zr_6(BDC)_4(TDC)_2$-MeOH is given in Figure S16. The peak at 7.73 ppm corresponds to the four aromatic protons of BDC and the peak at 7.35 to the two aromatic protons of TDC. The area ratio of these two peaks (BDC_{area}:TDC_{area} = 1:0.275) is normalised to the number of protons provides the molar ratio of BDC:TDC = 1:0.55, which can be written as BDC:TDC = 0.645:0.355. The peak at 3.22 ppm is assigned to the methyl protons of MeOH and the peak area ratio to BDC is BDC_{area}:$MeOH_{area}$ = 1:2.303, which corresponds to molar ratio BDC:MeOH = 1:3.07. The absence of any other proton signals and particularly those related to DMF and formic acid, which were used as solvent and modulator respectively, demonstrates that MeOH has exchanged all DMF from the pores and the formate ions that usually occupy the missing liker sites on the Zr cluster. It is anticipated that each formate ion has been replaced by a pair of MeO^-/MeOH.

The TGA under air of the previously discussed sample is shown in Figure S18. The mass loss up to 140 °C, 22%, corresponds to MeOH and adsorbed H_2O in the pores of the material and the small step around 250 °C corresponds to dehydroxylation of $[Zr_6O_4(OH)_4]^{12+}$ cluster to $[Zr_6O_6]^{12+}$. The mass loss between 400-800 °C, 39.57 %, is associated to the thermal decomposition of the organic linkers and the transformation of a compound with general formula $Zr_6O_6(BDC)_x(TDC)_y$ to ZrO_2 with residual mass of 35.63 %. The total amount of linkers was calculated by the TGA results and using the molar ratio of linkers BDC:TDC = 0.645:0.355 it is derived that there are 3.56 BDC and 1.96 TDC linkers per $[Zr_6O_4(OH)_4]^{12+}$ cluster. This composition corresponds to a formula $[Zr_6O_4(OH)_4(BDC)_{3.56}(TDC)_{1.96}]^{0.96+}$, where 0.48 linkers are missing from the ideal formula of the **fcu** net with 6 dicarboxylate linkers per cluster. In the $Zr_6(BDC)_4(TDC)_2$-DMF material it is expected that formate ions occupy the sites of carboxylates of missing linkers and the complete formula of the $Zr_6(BDC)_4(TDC)_2$-DMF framework can be expressed as $Zr_6O_4(OH)_4(BDC)_{3.56}(TDC)_{1.96}(HCOO)_{0.96}$. In $Zr_6(BDC)_4(TDC)_2$-MeOH material TDC has been disconnected from the cluster and replaced by four MeOH terminal ligands. Also, each formate is replaced by a pair of MeO^-/MeOH. The formula of the $Zr_6(BDC)_4(TDC)_2$-MeOH the framework is $Zr_6O_4(OH)_4(BDC)_{3.56}(TDC)_{1.96}(MeO^-)_{0.96}(MeOH)_{8.8}$. The amount of MeOH in $Zr_6(BDC)_4(TDC)_2$-MeOH analysed by NMR (BDC:MeOH = 1:3.07 or 10.9 MeOH per formula unit) exceeds the coordinate MeO^-/MeOH species, 9.8 in total per formula unit, and indicates that there is 1.1 MeOH per formula unit in the pores of $Zr_6(BDC)_4(TDC)_2$-MeOH. The mass loss of 22% up to 140°C, observed in TGA, accounts for the adsorbed and coordinated MeO^-/MeOH species (10.9 per formula unit) and 8 adsorbed

molecules of H_2O. The complete formula of the $Zr_6(BDC)_4(TDC)_2$-MeOH is $Zr_6O_4(OH)_4(BDC)_{3.56}(TDC)_{1.96}(MeO^-)_{0.96}(MeOH)_{8.8}\cdot 1.1MeOH\cdot 8H_2O$.

The composition analysis of the activated $Zr_6(BDC)_4(TDC)_2$ material has been also derived from the combination of TGA and ^1H NMR measurements. The ^1H NMR spectra of this sample (Figure S17) has a peak area ratio (BDC_{area}:TDC_{area} = 1:0.278) that corresponds to molar ratio BDC:TDC = 0.643:0.357 and it also shows the absence of any other organic components, e. g. formic acid or methanol, which can occupy the defect sites from missing linkers. TGA under air (Figure S19) shows mass loss of adsorbed H_2O up to 90 ^oC and the dehydroxylation step of the $[Zr_6O_4(OH)_4]^{12+}$ cluster at 250 ^oC. The mass loss between 400-800 °C, 46.73 %, is associated to the thermal decomposition of the organic linkers and the transformation of a compound with general formula $Zr_6O_6(BDC)_x(TDC)_y$ to ZrO_2 with residual mass of 42.98 %. The total amount of linkers was calculated by the TGA results and using the molar ratio of linkers BDC:TDC = 0.643:0.357 it is derived that there are 3.48 BDC and 1.93 TDC linkers per $[Zr_6O_4(OH)_4]^{12+}$ cluster. This composition corresponds to a formula $[Zr_6O_4(OH)_4(BDC)_{3.48}(TDC)_{1.93}]^{1.18+}$, where 0.59 linkers are missing from the ideal formula of the **fcu** net with 6 dicarboxylate linkers per cluster. In the activated $Zr_6(BDC)_4(TDC)_2$ material hydroxyl ions are occupying half of carboxylate O sites of missing linkers. The complete formula of the $Zr_6(BDC)_4(TDC)_2$ framework can be expressed as $Zr_6O_4(OH)_4(BDC)_{3.48}(TDC)_{1.93}(OH)_{1.18}$. It is commonly accepted that carboxylate O sites missing linker defects are occupied by OH^-/H_2O pairs, however the ^1H MAS NMR spectra of the activated $Zr_6(BDC)_4(TDC)_2$ (Figure S22) exhibited the presence of hydroxide (2.2 ppm) and aromatic (7.8 ppm) protons and confirmed the absence of H_2O. The ratio of peak areas of these two signals, Aromatic area: OH area = 3.2, is very close to the ratio of protons in the above formula, (BDC+TDC):OH = 3.43.

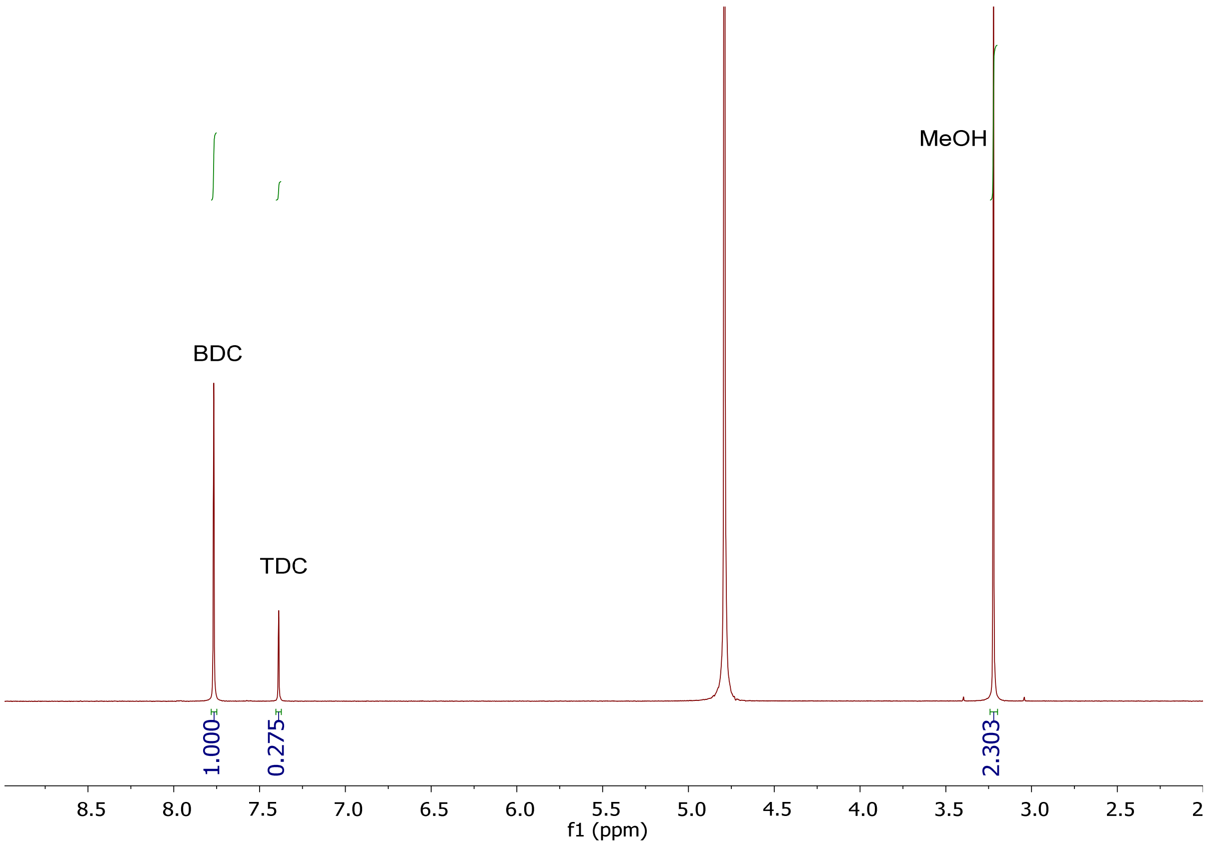

Figure S16 ^{1}H NMR spectrum of the sample of $Zr_6(BDC)_4(TDC)_2$-MeOH. The peak at 7.73 ppm is assigned to four aromatic protons of BDC, the peak at 7.4 ppm is assigned to two aromatic protons of TDC and the peak at 3.22 ppm is assigned to three methyl protons of MeOH. There are no formate (8.3 ppm), DMF (2.71 and 2.86 ppm), or dimethylamine (DMA, 2.15 ppm) related peaks in the spectrum, therefore the solvent exchange to MeOH was successful.

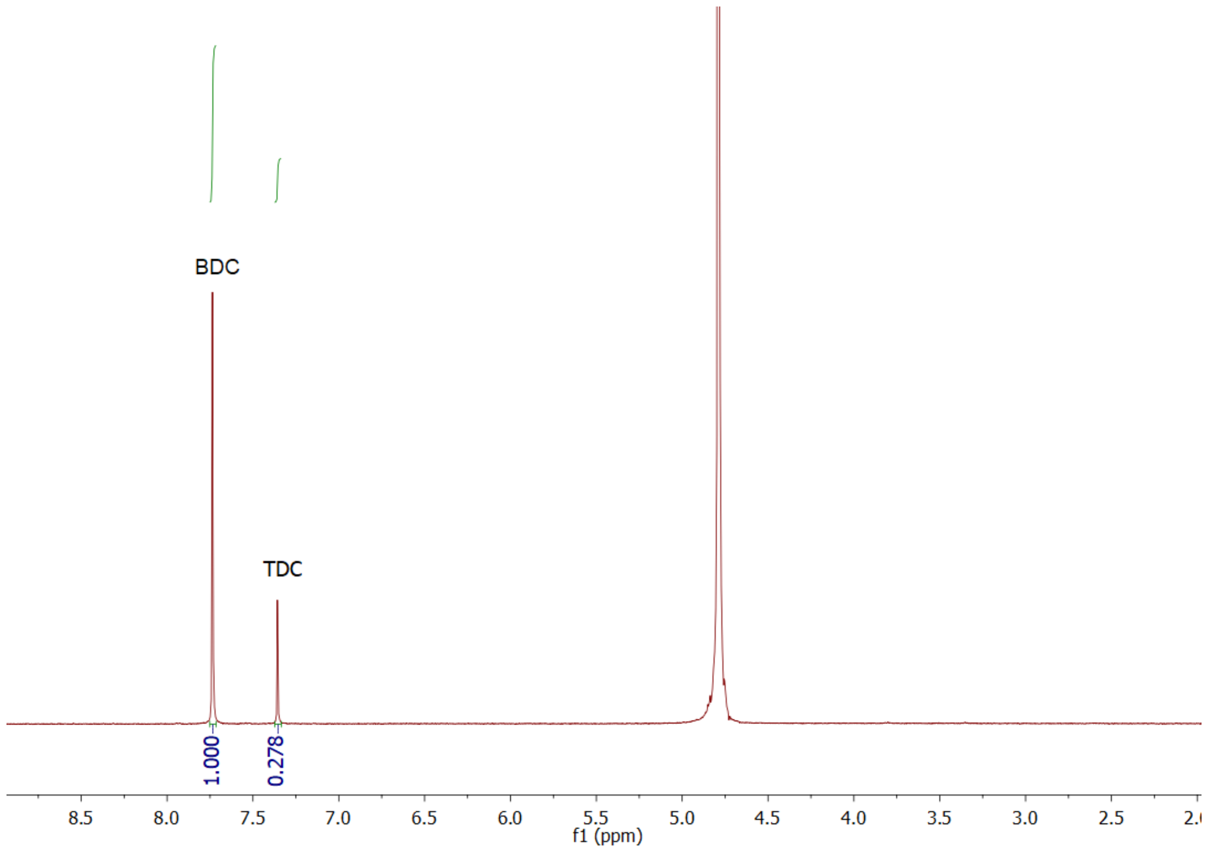

Figure S17 ^{1}H NMR spectrum of the sample of $Zr_6(BDC)_4(TDC)_2$. The peak at 7.73 ppm is assigned to four aromatic protons of BDC, the peak at 7.4 ppm is assigned to two aromatic protons of TDC.

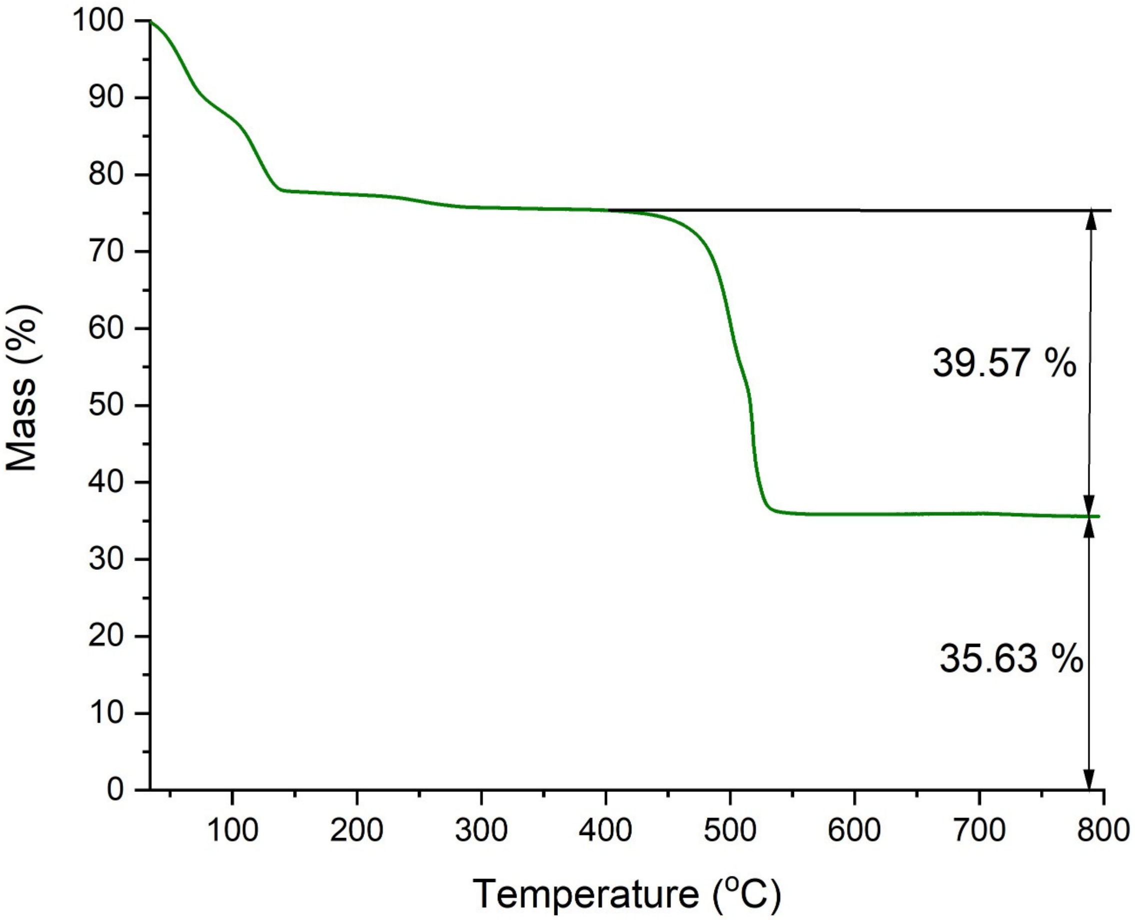

Figure S18 TGA of the $Zr_6(BDC)_4(TDC)_2$-MeOH sample under air at a flow rate of 60 mL min^{-1} and heating rate was 5 °C min^{-1}. The initial mass loss of 22% up to 140 °C is attributed to loss of methanol and adsorbed water from the material and the mass loss from 200 °C to 300 °C is attributed to the dehydroxylation of the clusters $[Zr_6O_4(OH)_4]^{12+}$ to $[Zr_6O_6]^{12+}$. The following mass loss is attributed to thermal decomposition of the organic linkers and is completed at 800 °C with mass loss of 39.57%. The residual 35.63% residual mass is accounted for by the ZrO_2 produced from the complete decomposition of the starting material.

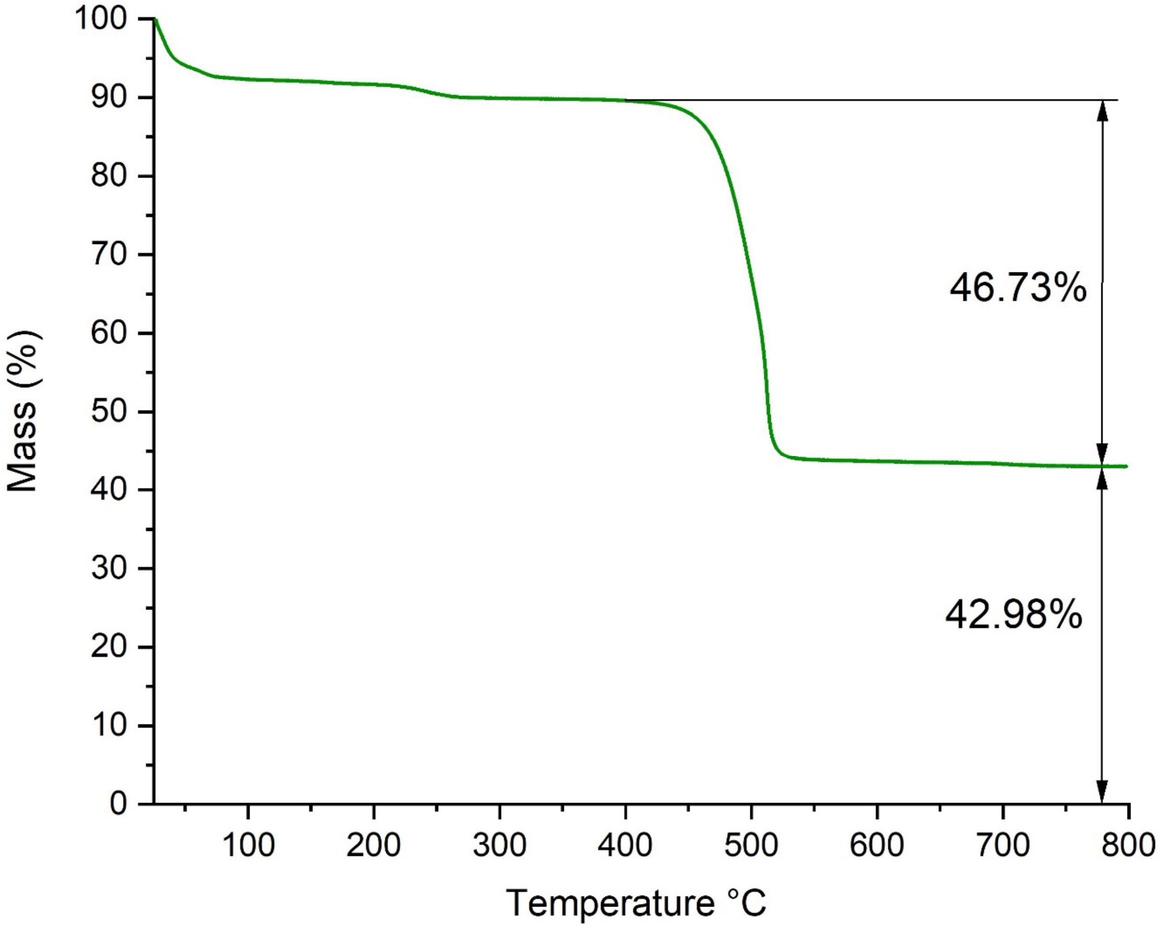

Figure S19 TGA of the activated $Zr_6(BDC)_4(TDC)_2$ sample under air at a flow rate of 60 mL min^{-1} and heating rate of 5 °C min^{-1}. The initial mass loss of 8% up to 90 °C is attributed to loss of adsorbed atmospheric water from the material and the mass loss of 2% from 200 °C to 300 °C is attributed to the dehydroxylation of the clusters $[Zr_6O_4(OH)_4]^{12+}$ to $[Zr_6O_6]^{12+}$. The following mass loss is attributed to thermal decomposition of the organic linkers and is completed at 800 °C with mass loss of 46.73%. The residual 42.98% residual mass is accounted for by the ZrO_2 produced from the complete decomposition of the starting material.

^1H NMR Data to Monitor the Replacement of Methanol by Atmospheric Water (Table S10 and Figure S20)

Table S10 Details of the experiments to monitor the replacement of methanol by atmospheric water including the different times of measurement, and the integration of the methanol signal from the measured ^1H NMR data (Figure S20 below).

^1H NMR spectrum	The length of time the sample was left open to atmosphere before prepared for digestion ^1H NMR (minutes)	Integration of MeOH signal
Figure S20 (a)	5	4.695
Figure S20 (b)	30	1.850
Figure S20 (c)	90	1.505
Figure S20 (d)	300	0.534
Figure S20 (e)	1440	0

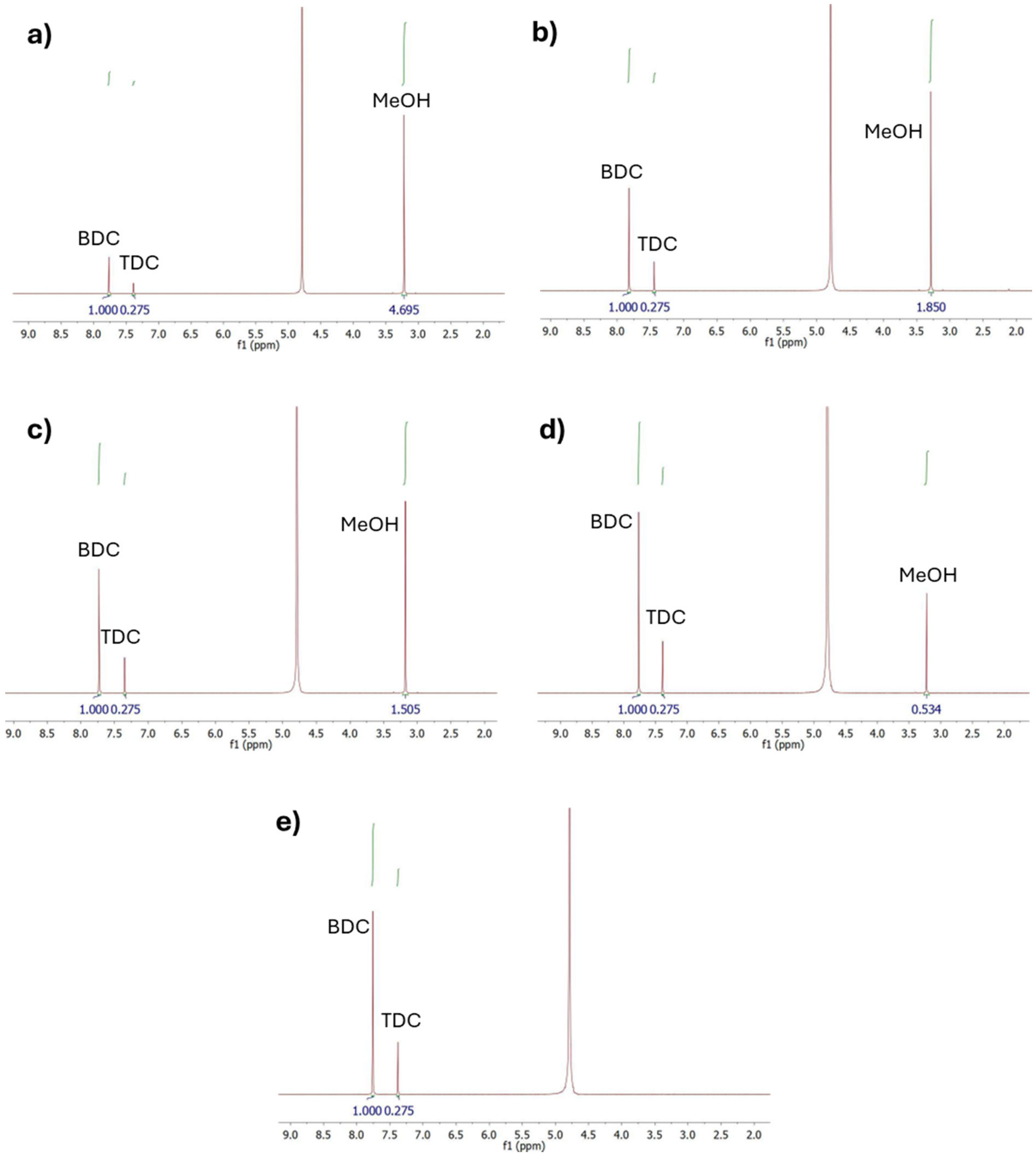

Figure S20 ^{1}H NMR spectra of the samples given in Table S10. The peak at 7.73 ppm is assigned to four aromatic protons of BDC, the peak at 7.4 ppm is assigned to two aromatic protons of TDC and the peak at 3.22 ppm is assigned to three methyl protons of MeOH.

Shorter Reaction Synthesis Results and PXRD Data (Table S11 and Figure S21)

Table S11 The details and observations of the reactions exploring shorter synthesis times of $Zr_6(BDC)_4(TDC)_2$-DMF. * This was a control reaction whereby the reaction was left to cool at the usual ramp down rate of 0.1 °C/min.

Quenching Time (hr)	Vial Observations
1	Opaque white solution *(small amount of solid obtained for measurement via centrifugation)*
3	A fine white powder settled at the bottom of the vial.
8	A fine white powder settled at the bottom of the vial, and a slight film on the side of the vial.
24	A fine white powder settled at the bottom of the vial, and a slight film on the side of the vial.
48	A fine white powder settled at the bottom of the vial, and a film on the side of the vial.
48*	A fine white powder settled at the bottom of the vial, and a film on the side of the vial.

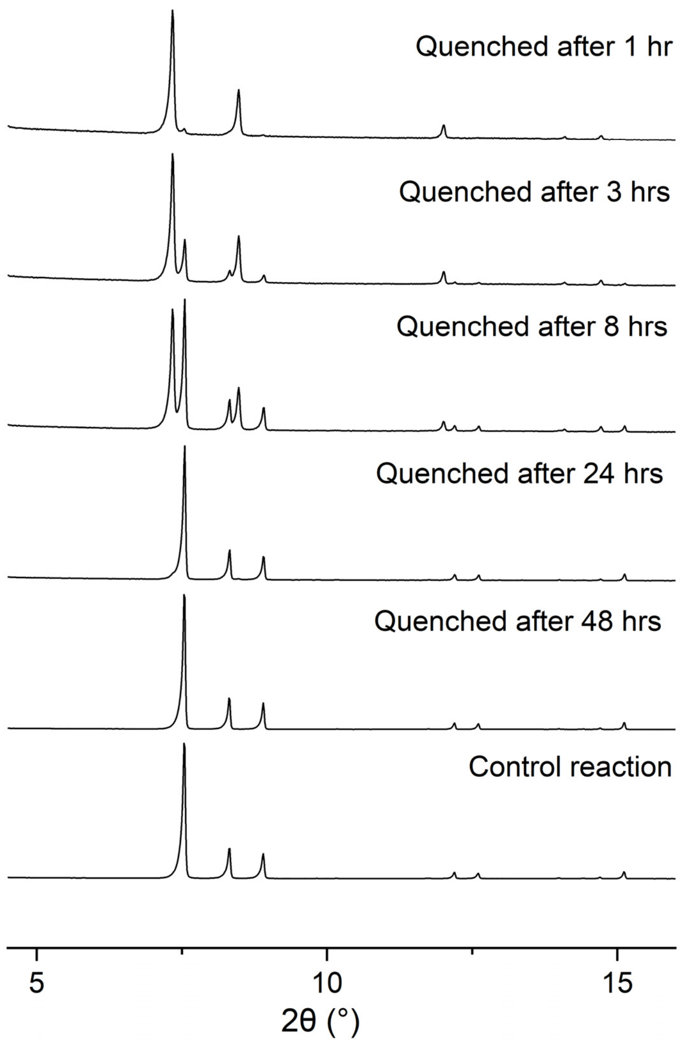

Figure S21 The in-house PXRD measurements (Cu $K_{\alpha1}$, λ = 1.5406 Å) of the quenched, and control, reactions of the $Zr_6(BDC)_4(TDC)_2$-DMF synthesis. Notably, despite the rapid formation of a UiO-66 phase, there is not the appearance of a solid solution type disordered cubic phase that could contain both linkers. Instead, a small amount of UiO-66 is formed first and gradually disappears as $Zr_6(BDC)_4(TDC)_2$ -DMF forms and become the only phase present in the reaction mixture.

Solid State MAS NMR Spectra (Figures S22 – S27 and Table S12)

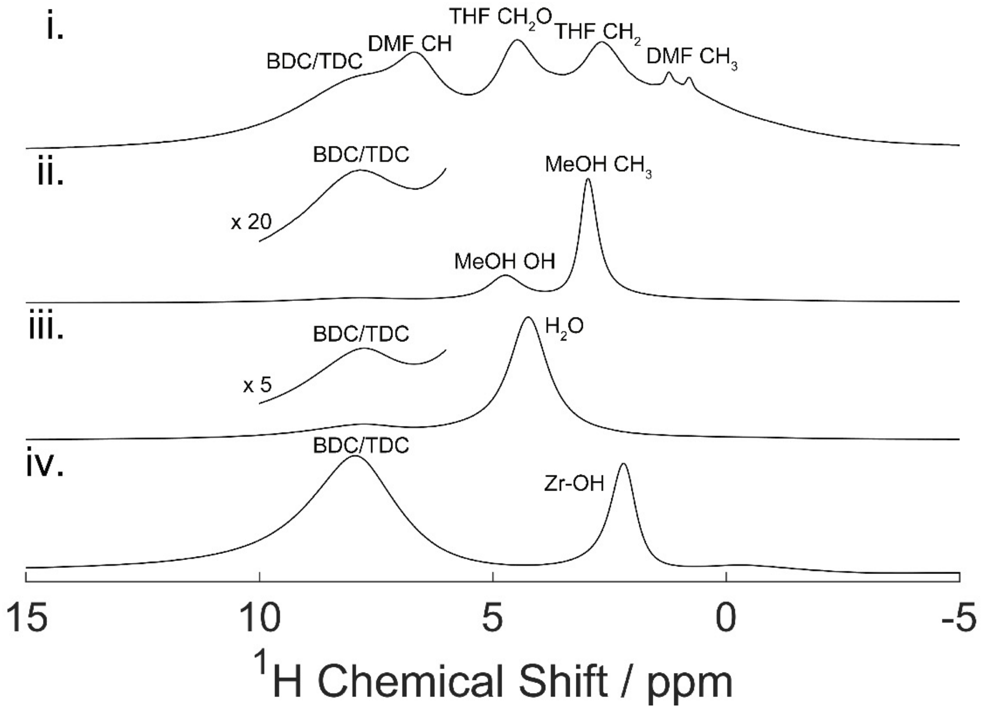

Figure S22 Hahn-echo detected ^{1}H MAS NMR spectra of i. $Zr_6(BDC)_4(TDC)_2$-DMF, ii, $Zr_6(BDC)_4(TDC)_2$-MeOH, iii. $Zr_6(BDC)_4(TDC)_2$-H_2O, and iv. $Zr_6(BDC)_4(TDC)_2$. All signal assignments are given in Table S12. The insets (10 to 6 ppm) in ii. and iii. show magnified views of the linker signals scaled up by the given factors to enhance visibility. The intensity ratio between the two signals in iv. is aromatic: Zr-OH = 3.2: 1.

Table S12 Signal assignments for the ^{1}H and ^{13}C MAS NMR spectra of all MOF samples. ^{13}C labels are given in the corresponding figures.

MOF Sample	^{1}H chemical shift / ppm	Assignment	^{13}C chemical shift / ppm	Assignment
$Zr_6(BDC)_4(TDC)_2$-DMF	7.8	Aromatic BDC and TDC protons	171.5	BDC-1
	6.7	DMF CH	171	BDC-1
	4.5	THF CH_2O	167	TDC-1
	2.7	THF CH_2	163	DMF CH
	1.2	DMF CH_3	145	TDC-2
	0.8	DMF CH_3	138	BDC-2
			133	TDC-3
			130	BDC-3
			69	THF CH_2O
			35	DMF CH_3
			31	DMF CH_3
			27	THF CH_2
$Zr_6(BDC)_4(TDC)_2$-MeOH	8.0	Aromatic BDC and TDC protons	171	BDC/TDC-1
	4.7	MeOH OH	144	TDC-2
	2.9	MeOH CH_3	138	BDC-2
			130	BDC/TDC-3
			54	MeOH (bound)
			52	MeOH (bound)
			50	MeOH (free)
$Zr_6(BDC)_4(TDC)_2$-H_2O	7.8	Aromatic BDC and TDC protons	172	BDC-1
	4.2	H_2O	171	TDC-1
			170	BDC-1
			144	TDC-2
			138	BDC-2
			137	BDC-2
			131	TDC-3
			129	BDC-3
$Zr_6(BDC)_4(TDC)_2$	7.8	Aromatic BDC and TDC protons	171	BDC-1
	2.2	Zr-OH	172	BDC-1
			168	TDC-2
			144	BDC-2
			136	TDC-3
			132	BDC-3

129	BDC-1

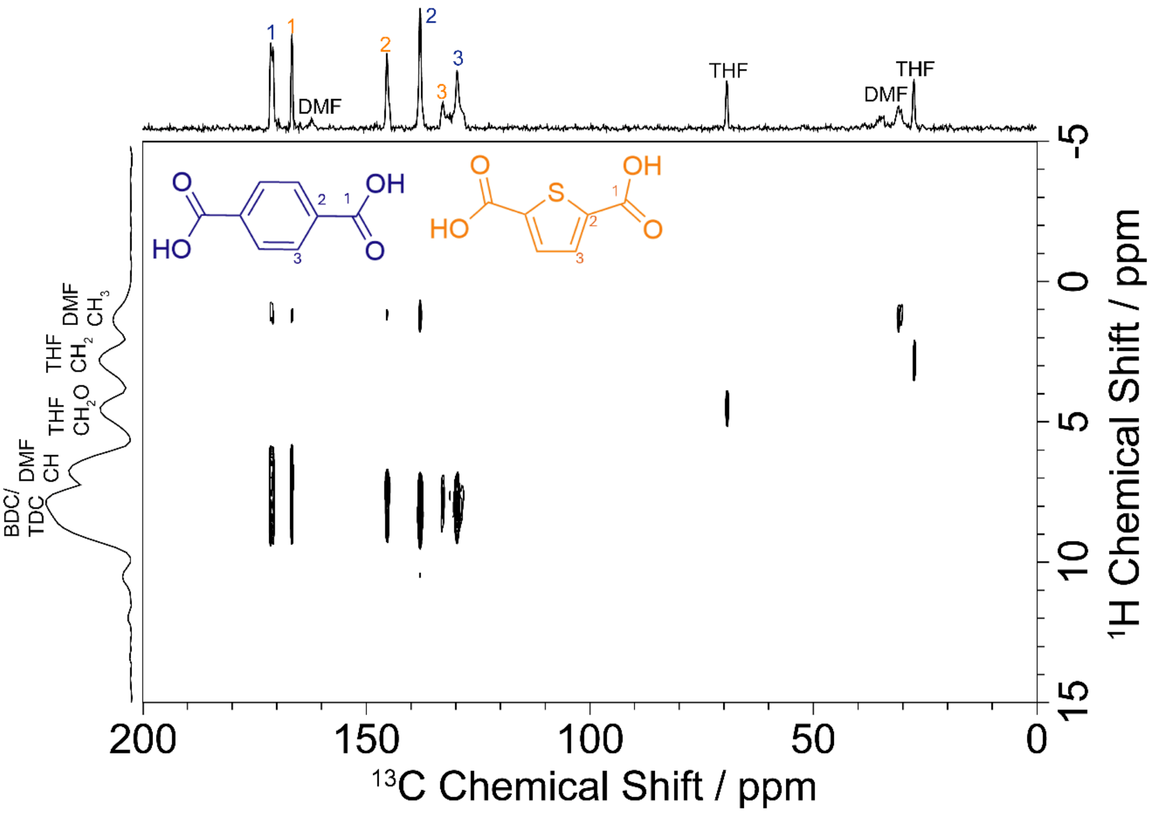

Figure S23 2D ^1H-^{13}C HETCOR NMR spectrum of $Zr_6(BDC)_4(TDC)_2$-DMF, including projections in both dimensions, measured using a CP contact time of 2 ms. Assignment of the ^1H solvent signals is aided by correlations to the ^{13}C solvent signals.

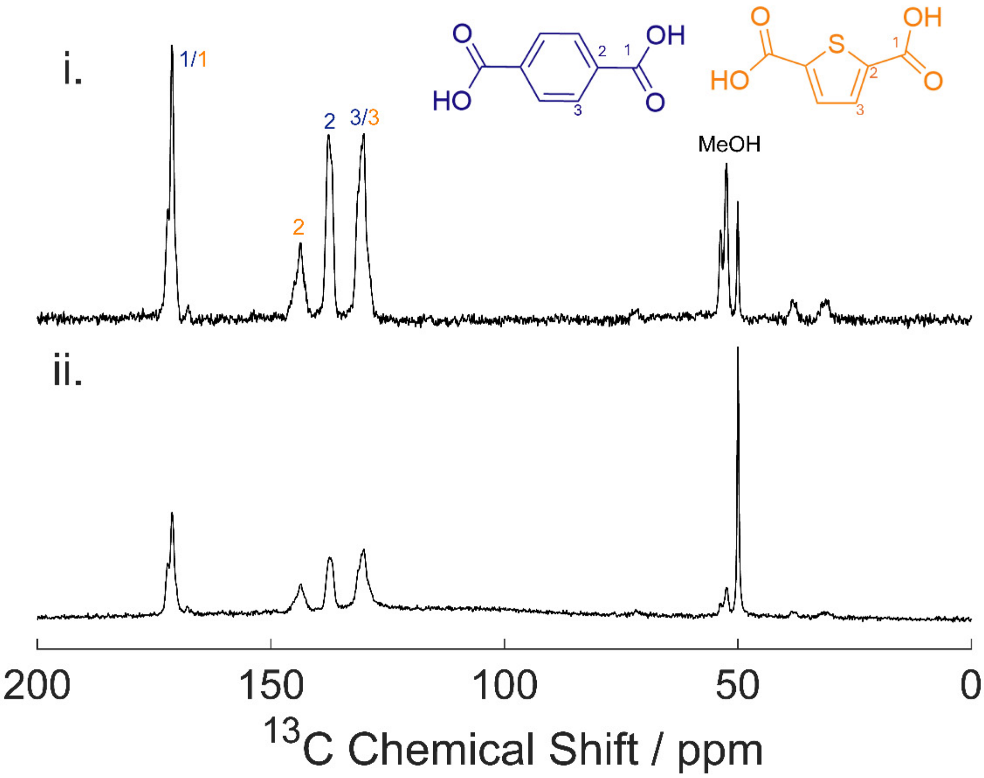

Figure S24 i. CP and ii. directly excited ^{13}C MAS NMR spectra of $Zr_6(BDC)_4(TDC)_2$-MeOH. The relative signal intensity of non-coordinated methanol at 50 ppm is larger in the directly excited spectrum than in the CP spectrum, as rapid tumbling limits the CP efficiency.

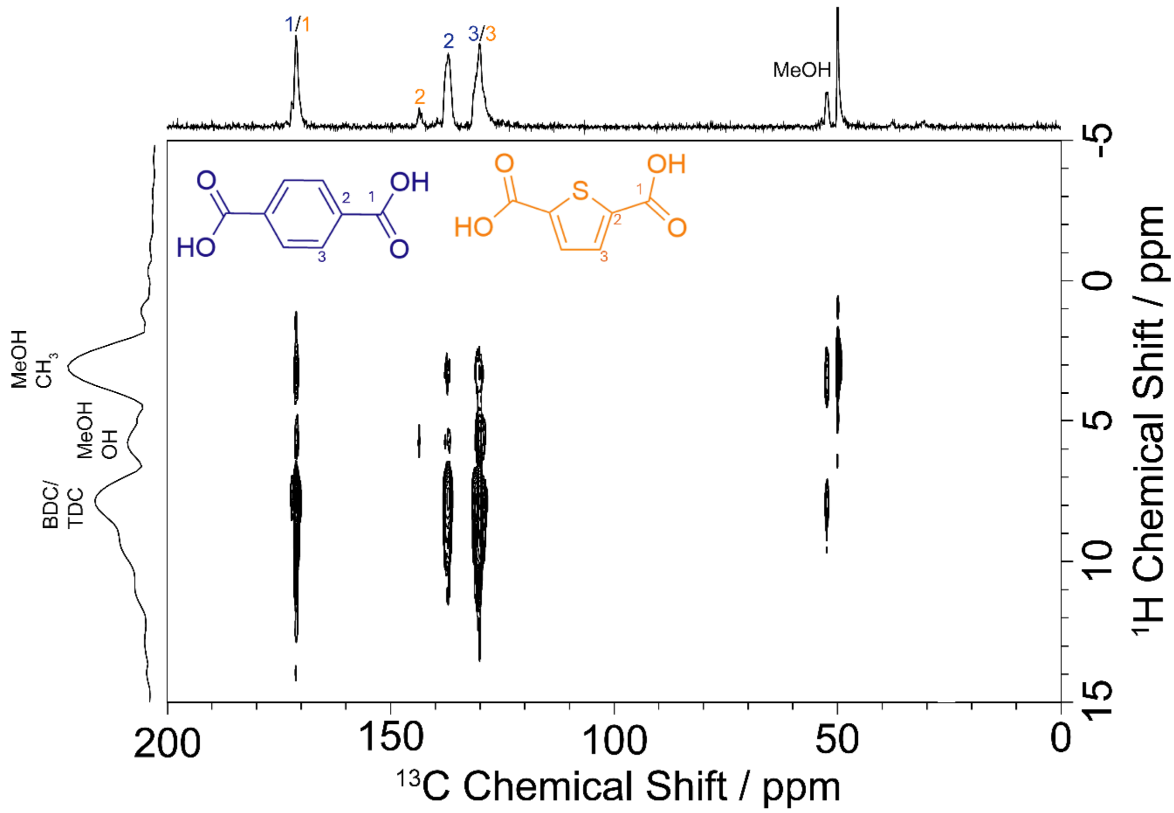

Figure S25 2D ^1H-^{13}C HETCOR NMR spectrum of $Zr_6(BDC)_4(TDC)_2$-MeOH, including projections in both dimensions, measured using a CP contact time of 2 ms. Correlations are seen between the carbons of bound methanol at 52 ppm, and aromatic linker protons at 8 ppm, as well between the methanol ^1H signals (2.9 and 5.7 ppm, with the latter being obscured by the more intense unbound methanol signal at 4.8 ppm in the ^1H Hahn echo spectrum, Figure S22) and the carboxylate carbons (171 ppm).

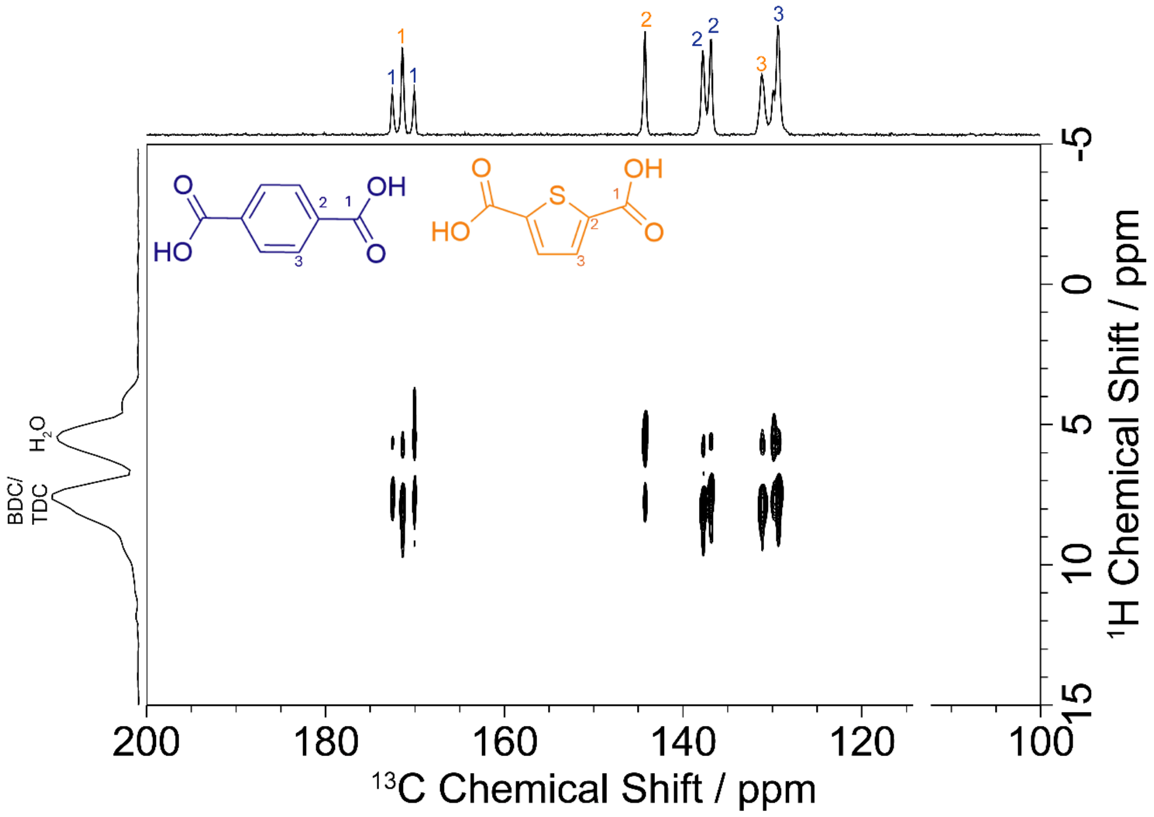

Figure S26 2D ^1H-^{13}C HETCOR NMR spectrum of $Zr_6(BDC)_4(TDC)_2$-H_2O, including projections in both dimensions, measured using a CP contact time of 2 ms. Correlations are seen between H_2O bound to Zr_6 cluster (5.4 ppm, obscured by the unbound H_2O signal at 4.2 ppm in the ^1H Hahn echo spectrum, Figure S22) and all ^{13}C linker signals.

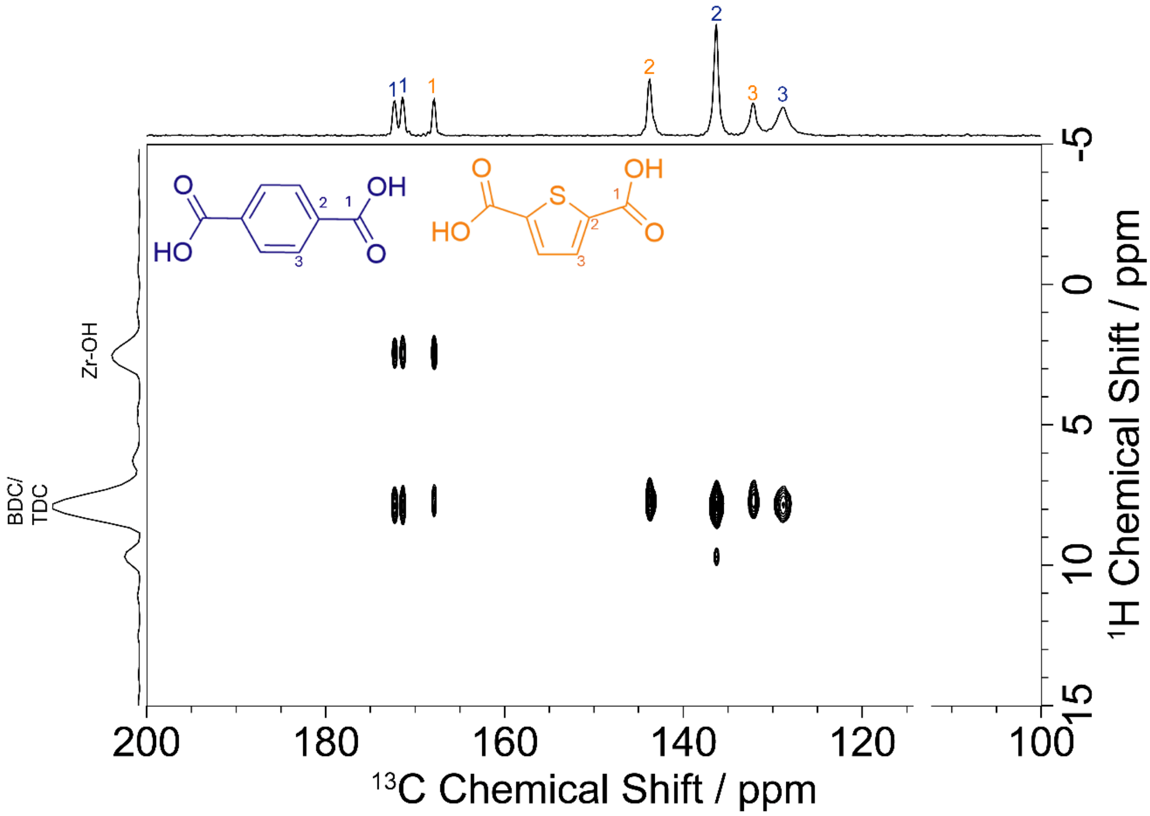

Figure S27 2D ^{1}H-^{13}C HETCOR NMR spectrum of $Zr_6(BDC)_4(TDC)_2$, including projections in both dimensions, measured using a CP contact time of 2 ms. Correlations are seen between the carboxylate carbons (175-165 ppm) and the μ_3-OH protons (2.2 ppm).

Variable temperature PXRD data of $Zr_6(BDC)_4(TDC)_2$-H_2O (Figure S28)

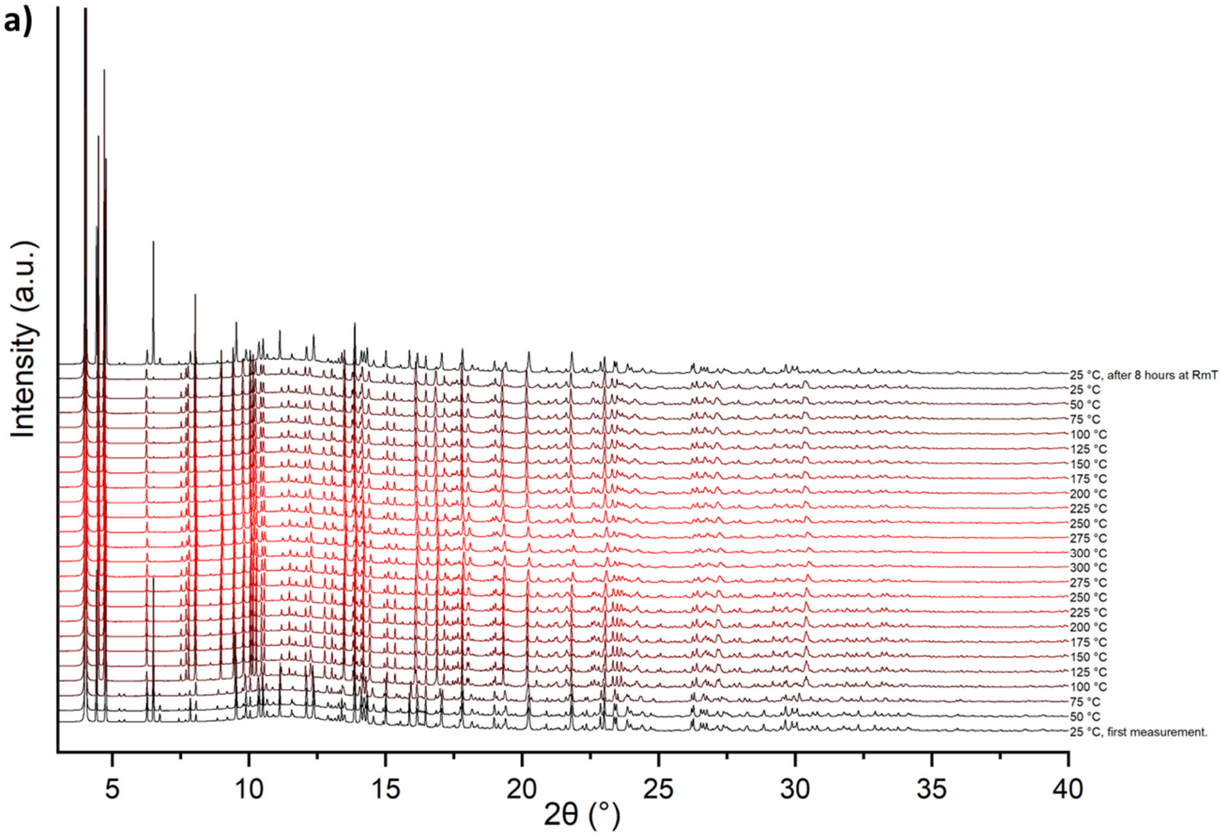

Figure S28 In situ variable temperature synchrotron PXRD measurements on an open-ended capillary, with the powder secured by a small amount of glass wool. The PXRD patterns in a) were measured from 25 - 300, 300 - 25 °C in steps of 25 °C. The data is coloured with a black - red - black gradient. The bottom pattern was the first room temperature measurement. The top pattern in a) was measured after the open capillary was left 8 hours at room temperature. The ramp rate was 12 °C/min and each temperature was held for 5 minutes before the measurement.

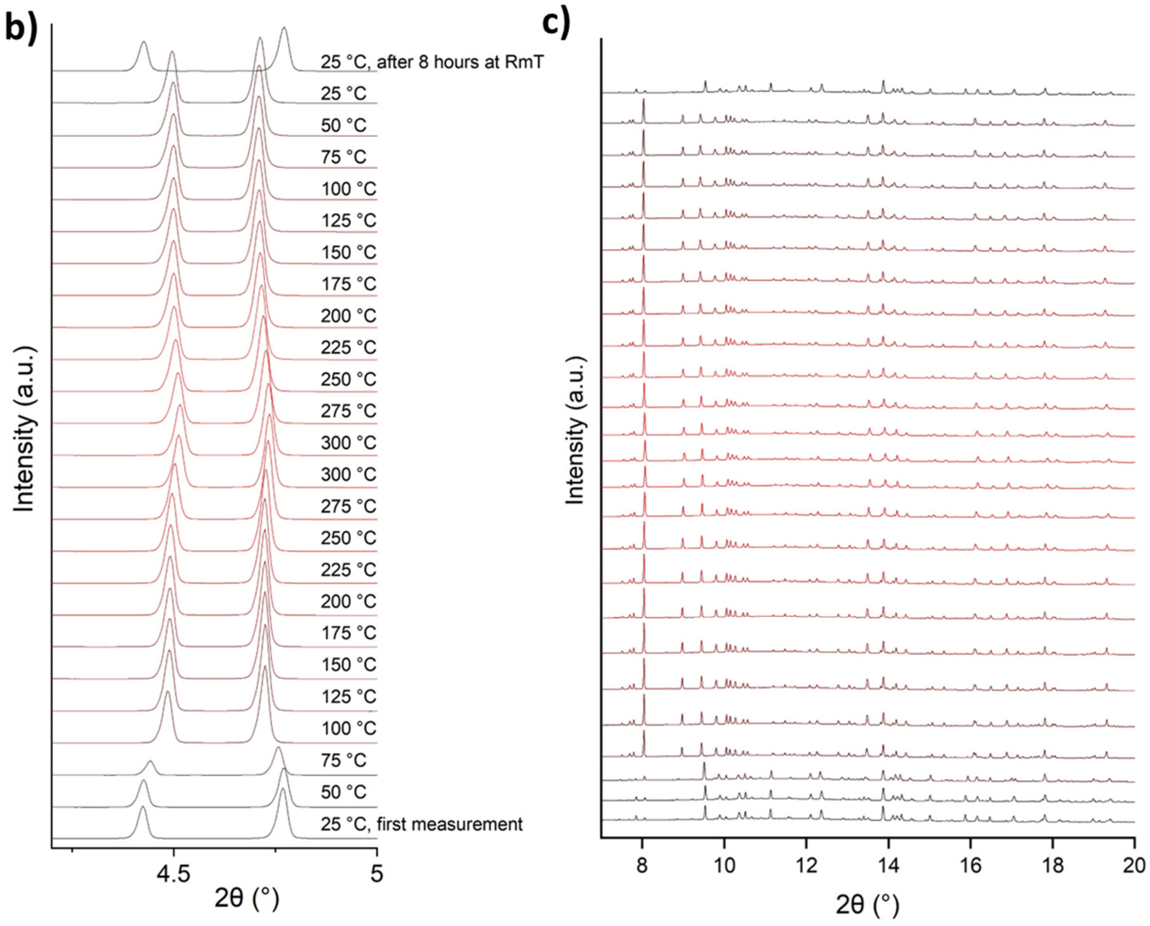

Figure S28. b) The same PXRD patterns from S24 (a), shown in the 4 -5 2θ range, and c) the 7-20 2θ range to show the transition from $Zr_6(BDC)_4(TDC)_2$-H_2O to $Zr_6(BDC)_4(TDC)_2$ and back to $Zr_6(BDC)_4(TDC)_2$-H_2O as atmospheric water re-enters the framework.

Pore size distribution of $Zr_6(BDC)_4(TDC)_2$ (Figure S29)

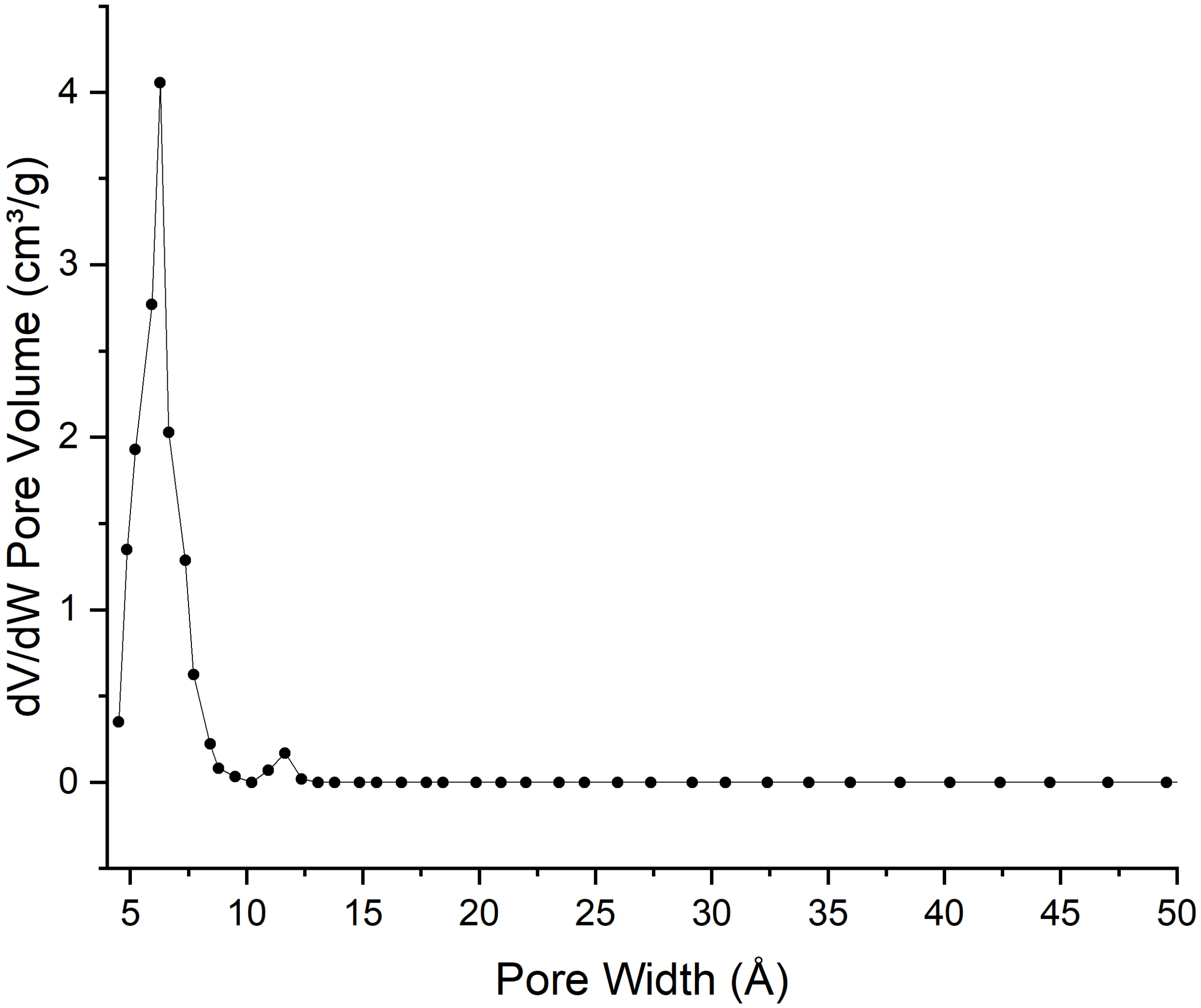

Figure S29 The pore size distribution of the $Zr_6(BDC)_4(TDC)_2$ N_2 isotherm (Figure 9a) as calculated with the model N_2 - Tarazona NLDFT. The maximum of the distribution is at 6 Å.

Porous Features of UiO-66, DUT-67, and $Zr_6(BDC)_4(TDC)_2$ (Figure S30)

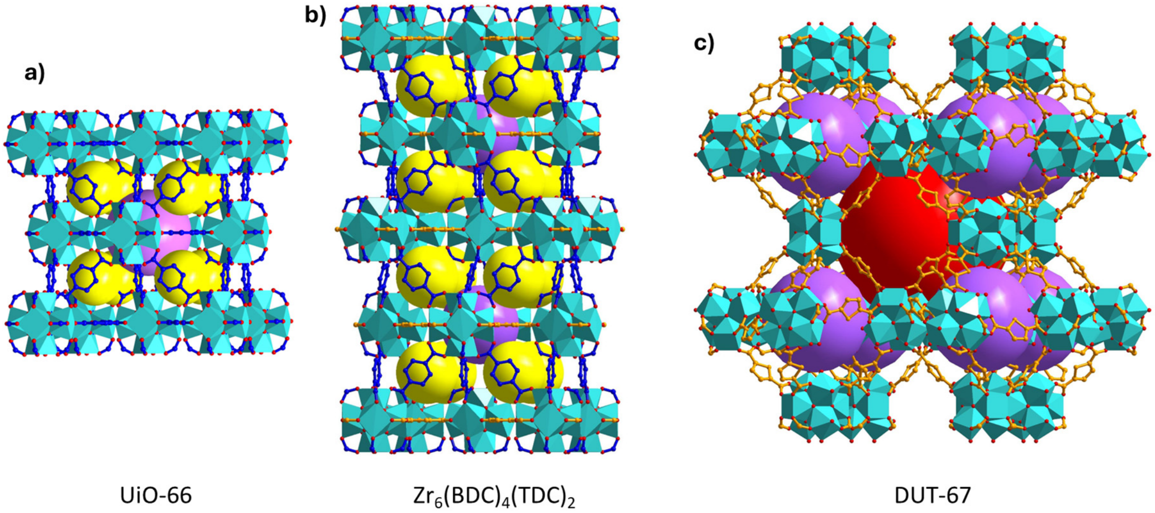

Figure S30 Structural representation to highlight the porous features of (a) UiO-66, (b) $Zr_6(BDC)_4(TDC)_2$, and (c) DUT-67. UiO-66 and $Zr_6(BDC)_4(TDC)_2$ are both **fcu** topology, and contain tetrahedral (yellow spheres) and octahedral (purple spheres) cages. DUT-67 is **reo** topology and contains octahedral (purple spheres) and cuboctahedral (red spheres) cages. In UiO-66 and $Zr_6(BDC)_4(TDC)_2$ the narrowest part of the guest diffusion path is the triangular window that connects the two cages. In DUT-67 the diffusion of a guest can occur through the square shaped window of the cuboctahedral cage.

Gas Chromatography of $Zr_6(BDC)_4(TDC)_2$ (Tables S13– S15 and Figure S31)

Thermodynamic parameters of the adsorption process

The zero-coverage thermodynamic parameters of the adsorption process were calculated using a van’t Hoff type analysis employing isothermal chromatographic measurements (Supplementary Tables S13-15 and Supplementary Figure S31). The retention volumes were corrected taking into account the volume expansion of the gas entering the capillary due to the temperature increase according to

$$V_N = (t_r - t_m) \cdot F_a \cdot (T/T_a) \cdot j$$

where, V_N = net retention volume (mL), t_r = retention time (min), t_m = dead time (min), F_a = volumetric constant flow-rate measures at ambient temperature (mL·min^{-1}), T = column temperature (K), T_a = ambient temperature (K), j = James–Martin gas compressibility correction = $[3(p_i/p_0)^2-1][(2(p_i/p_0)^3-1)]^{-1}$, where p_i = pressure of gas applied to the chromatogram and p_0 = pressure of gas at outlet.

Using the data of V_N at different temperatures of each analyte, the isosteric heat of adsorption ΔH_{iso} and the entropy of adsorption ΔS_{ads} can be calculated via a van't Hoff type analysis, according to

$$\ln(V_N) = \ln(RTn_s) + \Delta S_{ads}/R - \Delta H_{diff}/(RT')$$

In standard gas chromatographic experiments, the term $\ln(RTn_s)$ is usually small and can be neglected in the determination of ΔS_{ads}. In addition to the ΔH_{diff} value obtained from the van’t Hoff plot the isosteric heat of adsorption (ΔH_{iso}) was determined according to the relation

$$|\Delta H_{iso}| = |\Delta H_{diff}| + RT_{ave}.$$

Table S13 Adjusted retention times t_R ($t_r - t_m$), logarithm of the retention volumes (ln V_N) of the studied analytes at different temperatures for $Zr_6(BDC)_4(TDC)_2$.

C6 Analyte	T (K)	t_r (min)	t_m (min)	t_R (min)	ln V_N
n-Hexane	493.15	11.89	0.33	11.56	3.908
	503.15	8.876	0.315	8.561	3.613
	513.15	6.856	0.302	6.554	3.351
	523.15	5.296	0.291	5.005	3.086
Benzene	493.15	36.956	0.33	36.626	5.062
	503.15	27.839	0.315	27.524	4.781
	513.15	21.478	0.302	21.176	4.524
	523.15	16.542	0.291	16.251	4.264
Cyclohexane	493.15	121.414	0.33	121.08	6.257
	503.15	87.676	0.315	87.361	5.936
	513.15	65.982	0.302	65.68	5.656
	523.15	51.455	0.291	51.164	5.411

Table S14 Linear regression functions corresponding to the van't Hoff type analyses of the studied analytes for the determination of ΔH_{iso} and ΔS_{ads}.

C6 Analyte	T_{ave} (K)	Equation	R^2
n-Hexane	508.15	ln V_N = 7041.58 ± 73.21/T -10.37 ± 0.14	0.9998
Benzene	508.15	ln V_N = 6838.17 ± 46.42/T -8.81 ± 0.09	0.9999
Cyclohexane	508.15	ln V_N = 7281.25 ± 220.38/T -8.52 ± 0.43	0.9982

Table S15 Values of enthalpy, entropy and Gibbs free energy of adsorption of the studied analytes were obtained from the variable temperature pulse gas chromatographic studies using the $Zr_6(BDC)_4(TDC)_2$ packed column. The free energy was calculated using $\Delta G = \Delta H_{iso} - T_{ave} \cdot \Delta S_{ads}$. The ΔH_{iso} and ΔS_{ads} values for UiO-66 were obtained from T. Duerinck et al.[25] These were used to calculate ΔG. The error (δ) for ΔG was calculated by $\delta \Delta G = \sqrt{(\delta \Delta H)^2 + (T_{\text{ave}} * \delta \Delta S)^2}$. The reported results were measured on a 35 cm column with an internal diameter of 0.3175 cm with an average temperature of 523 K. Values marked with an '*' did not have errors reported.

C6 Analyte	T_{ave} (K)	ΔH_{iso} (kJ mol^{-1})	ΔS_{ads} (J K^{-1} mol^{-1})	ΔG (kJ mol^{-1})
		$Zr_6(BDC)_4(TDC)_2$		
n-Hexane	508.15	-62.8 ± 0.6	-86.3 ± 1.2	-19.0 ± 0.9
Benzene	508.15	-61.1 ± 0.4	-73.2 ± 0.8	-23.9 ± 0.5
Cyclohexane	508.15	-64.8 ± 1.1	-70.8 ± 3.6	-28.8 ± 2.1
		UiO-66 Literature Values		
n-Hexane	523	-52.8±0.7	-66.3±1.3	-18.13±0.98
Benzene	523	-51.60*	-73.30*	-24.36*
Cyclohexane	523	-59.9±0.7	-63.8±1.2	-26.53±0.94

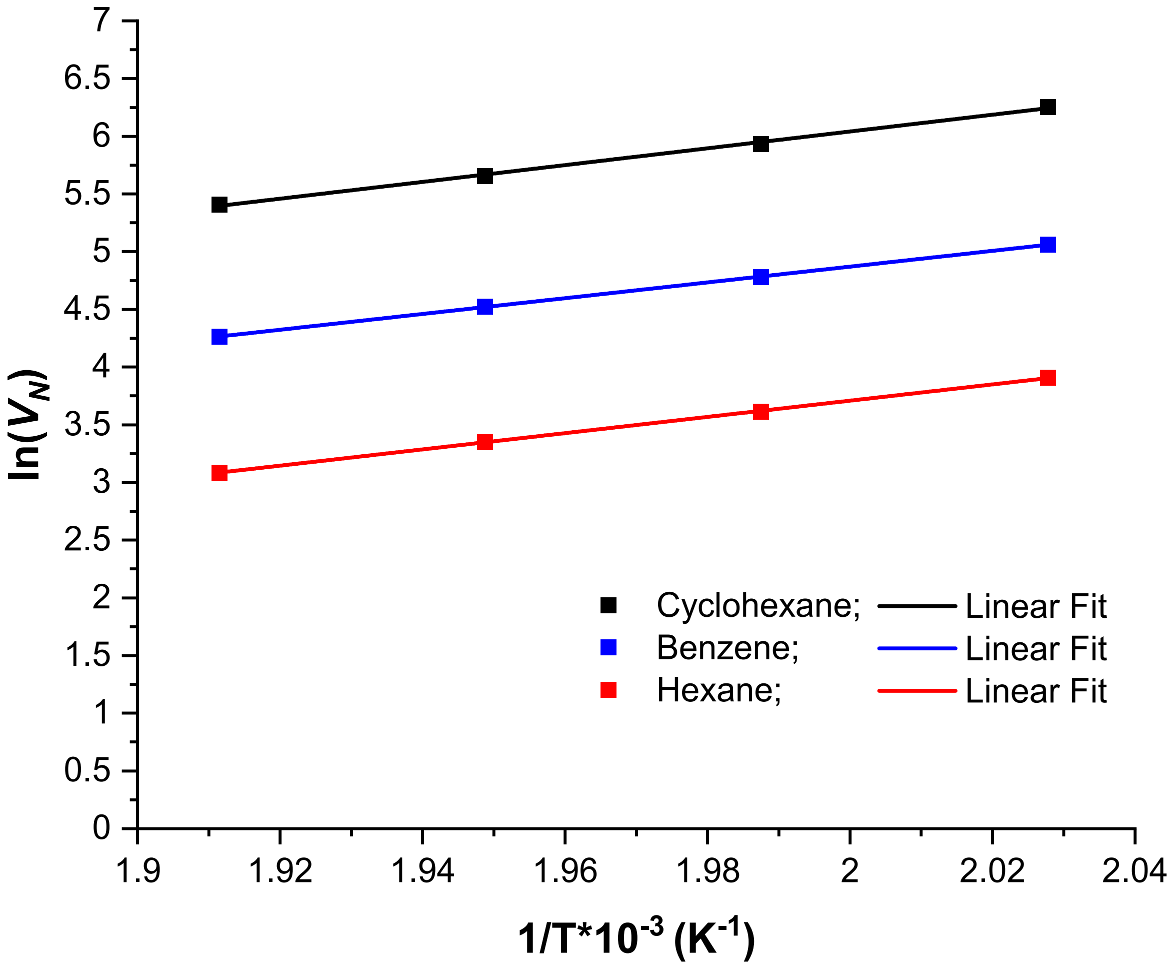

Figure S31 The fitting of the variation of the retention volume, ln(V_N) (from Table 13 above), as a function of the adsorption temperature (493.15 K, 503.15 K, 513.15 K, and 523.15 K) for the $Zr_6(BDC)_4(TDC)_2$ column.

Vapour adsorption-desorption isotherms (Figure S32)

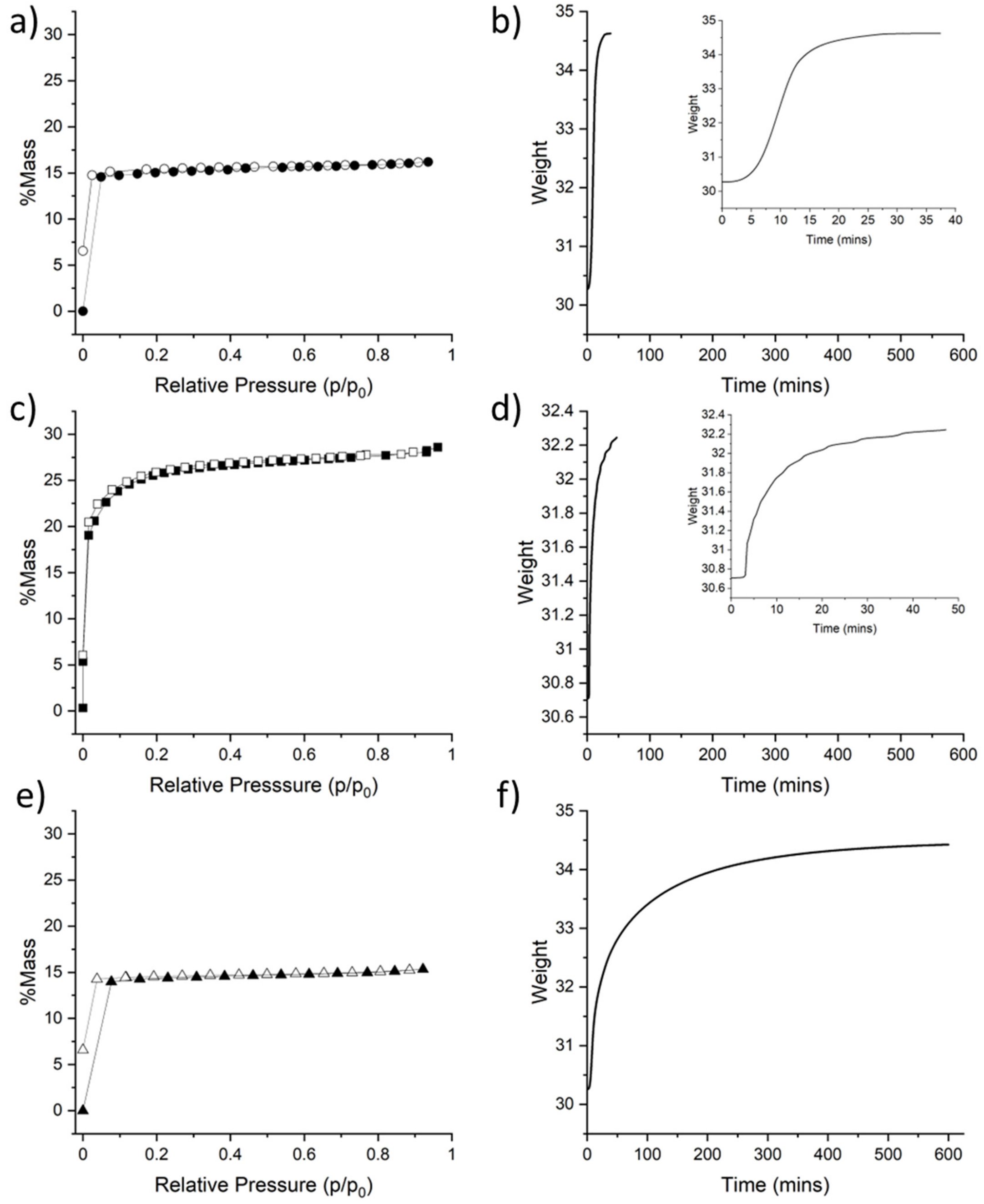

Figure S32 (a) The vapour adsorption – desorption isotherm of *n*-hexane in $Zr_6(BDC)_4(TDC)_2$, upon saturation at P/P_0 = 0.8 the total uptake was 15.9 %mass. The closed circles correspond to the adsorption branch and the open circles to the desorption branch. (b). The plot of weight (mg) vs time to show the kinetics of the first pressure point of the *n*-hexane isotherm, whereby the system reached equilibrium after 35 minutes. (c) The vapour adsorption – desorption isotherm of benzene in $Zr_6(BDC)_4(TDC)_2$, upon saturation at P/P_0 = 0.8 the total uptake was 27.8 %mass. The closed squares correspond to the adsorption branch and the open squares to the desorption branch. (d). The plot of weight (mg) vs time to show the kinetics of the first pressure point of the benzene isotherm, whereby the system reached equilibrium after 48 minutes. (e) The vapour adsorption – desorption isotherm of cyclohexane

in $Zr_6(BDC)_4(TDC)_2$, upon saturation at P/P_0 = 0.8 the total uptake was 15.1 %mass. The closed triangles correspond to the adsorption branch and the open triangles to the desorption branch. (f). The plot of weight (mg) vs time to show the kinetics of the first pressure point of the cyclohexane hexane isotherm, whereby the system reached equilibrium in 600 minutes. The total uptake of the guests relates to the packing of the molecules within the framework and the available porous space. The weight vs time plots are to illustrate the relative kinetics of the different isotherms. The faster kinetics of hexane and benzene are shown with insets in (b) and (d).

Molecular Dynamics Simulations (Table S16)

To compare guest dynamics in UiO-66 and $Zr_6(BDC)_4(TDC)_2$, we performed MD simulations under constant NVT conditions using machine learnt potentials mace-mp-0b3 with dispersion correction included.[26] All calculations were performed using Atomic Simulation Environment (ASE)[27] on Isambard 3 Tier-2 HPC Facility using a single node per run. A typical 50 ps MD run for a system of over 1000 atoms required approximately a week to compute.

The simulation cell for $Zr_6(BDC)_4(TDC)_2$ consisted of a single unit cell of this material with 864 atoms. To match the number of linkers and SBU units, we used a 1x1x2 supercell of UiO-66 containing 912 atoms. Multiple guest molecules of *n*-hexane, benzene and cyclohexane were placed in these simulations cell, either in tetrahedral or octahedral cages to make it possible to observe the trajectories of multiple guests while still being in the low-loading regime where diffusion of each guest molecule is not affected by the presence of other guest molecules.

Once a predefined number of guests (12 for tetrahedral cages and 8 for octahedral cages) have been individually placed in the cages of a specific type, each host+guests configuration was optimised using BFGS method with the default force tolerance threshold of 0.05 eV/Å. These optimised configurations were then used to start MD simulations.

The MD simulations were performed using Langevin dynamics with temperature set to T=523 K and friction parameter set to 0.002, providing a very weak coupling to the heat bath and ensuring that the thermostat has little effect on guest trajectories. Each MD run consisted of 50,000 MD steps, 1 fs timestep, the locations of the guests were recorded every 10 timesteps. To minimise thermal equilibration time when each MD run began, we initialised Maxwell Boltzmann distribution of velocities at 2T rather than T. This allowed the injection of energy equal to $3Nk_BT$ into the system which within 10 MD steps got distributed equally between kinetic and potential degrees of freedom,[28] while maintaining the total energy close to the value expected at T=523 K. To calculate mean residence time of guests in octahedral cage shown in Figure S16, we repeated each MD run three times starting with a different random guest orientation in the centre of every tetrahedral cage in a cell each time. Thus 3x8=24 individual guest trajectories starting in an octahedral cage and finishing in a tetrahedral cage were analysed for each of the three guests in each MOF.

Table S16. The mean simulated time for a guest molecule located in the centre of the octahedral cage to migrate to a neighbouring tetrahedral cage at 523 K. Each residence time is averaged over 24 guest trajectories with the error bars giving the standard deviation of the mean. The numbers in brackets for $Zr_6(BDC)_4(TDC)_2$ give the number of times the guest entered a tetrahedral cage through the wide window vs the narrow window that are presented in Figures 8a and b respectively.

	n-Hexane	Benzene	Cyclohexane
$Zr_6(BDC)_4(TDC)_2$	4.4±0.7 ps (15 vs 9)	6.5±1.1 ps (22 vs 2)	13.3±2.9 ps (16 vs 8)
UiO-66	3.3±0.7 ps	5.7±0.7 ps	5.5±0.9 ps

References

(1) Tollitt, A. M.; Vismara, R.; Daniels, L. M.; Antypov, D.; Gaultois, M. W.; Katsoulidis, A. P.; Rosseinsky, M. J. High-throughput discovery of a rhombohedral twelve-connected zirconium-based metal-organic framework with ordered terephthalate and fumarate linkers. *Angewandte Chemie International Edition* **2021**, *4*, 1-17. DOI: 10.1002/anie.202108150.

(2) Willems, T. F.; Rycroft, C. H.; Kazi, M.; Meza, J. C.; Haranczyk, M. Algorithms and tools for high-throughput geometry-based analysis of crystalline porous materials. *Microporous and Mesoporous Materials* **2012**, *149* (1), 134-141. DOI: 10.1016/j.micromeso.2011.08.020.

(3) Ito, S.; White, F. J.; Okunishi, E.; Aoyama, Y.; Yamano, A.; Sato, H.; Ferrara, J. D.; Jasnowski, M.; Meyer, M. Structure determination of small molecule compounds by an electron diffractometer for 3D ED/MicroED. *CrystEngComm* **2021**, *23* (48), 8622-8630. DOI: 10.1039/d1ce01172c.

(4) Truong, K.-N.; Ito, S.; Wojciechowski, J. M.; Göb, C. R.; Schürmann, C. J.; Yamano, A.; Del Campo, M.; Okunishi, E.; Aoyama, Y.; Mihira, T.; Hosogi, N.; Benet-Buchholz, J.; Escudero-Adán, E. C.; White, F. J.; Ferrara, J. D.; Bücker, R. Making the Most of 3D Electron Diffraction: Best Practices to Handle a New Tool. *Symmetry* **2023**, *15* (8). DOI: 10.3390/sym15081555.

(5) Rigaku Oxford Diffraction, CrysAlis^{Pro} software system, Rigaku Corporation, Wrocław, Poland, **2025** (version 1.171.44.103a)

(6) Sheldrick, G. SHELXT - Integrated space-group and crystal-structure determination. *Acta Crystallographica Section A* **2015**, *71* (1), 3-8. DOI: 10.1107/S2053273314026370.

(7) Sheldrick, G. Crystal structure refinement with SHELXL. *Acta Crystallographica Section C* **2015**, *71* (1), 3-8. DOI: 10.1107/S2053229614024218.

(8) Dolomanov, O. V.; Bourhis, L. J.; Gildea, R. J.; Howard, J. A. K.; Puschmann, H. OLEX2: A Complete Structure Solution, Refinement and Analysis Program. *Journal of Applied Crystallography* **2009**, *42* (2), 339-341. DOI: 10.1107/S0021889808042726.

(9) Rigaku Oxford Diffraction, AutoChem 6 software system in conjunction with OLEX2, Rigaku Corporation, Wrocław, Poland, **2025** (version 1.5-ac7-013).

(10) Fung, B. M.; Khitrin, A. K.; Ermolaev, K. An Improved Broadband Decoupling Sequence for Liquid Crystals and Solids. *Journal of Magnetic Resonance* **2000**, *142* (1), 97-101. DOI: 10.1006/jmre.1999.1896.

(11) Harris, R. K.; Becker, E. D.; Cabral De Menezes, S. M.; Granger, P.; Hoffman, R. E.; Zilm, K. W.; International Union of, P.; Applied Chemistry, P.; Biophysical Chemistry, D. Further conventions for NMR shielding and chemical shifts IUPAC recommendations 2008. *Solid State Nucl Magn Reson* **2008**, *33* (3), 41-56. DOI: 10.1016/j.ssnmr.2008.02.004.

(12) Hirsh, D. A.; Wijesekara, A. V.; Carnahan, S. L.; Hung, I.; Lubach, J. W.; Nagapudi, K.; Rossini, A. J. Rapid Characterization of Formulated Pharmaceuticals Using Fast MAS (1)H Solid-State NMR Spectroscopy. *Mol Pharm* **2019**, *16* (7), 3121-3132. DOI: 10.1021/acs.molpharmaceut.9b00343.

(13) Morcombe, C. R.; Zilm, K. W. Chemical shift referencing in MAS solid state NMR. *J Magn Reson* **2003**, *162* (2), 479-486. DOI: 10.1016/s1090-7807(03)00082-x.

(14) van Rossum, B. J.; Förster, H.; de Groot, H. J. M. High-Field and High-Speed CP-MAS13C NMR Heteronuclear Dipolar-Correlation Spectroscopy of Solids with Frequency-Switched Lee–Goldburg Homonuclear Decoupling. *Journal of Magnetic Resonance* **1997**, *124* (2), 516-519. DOI: 10.1006/jmre.1996.1089

(15) Coelho, A. Indexing of powder diffraction patterns by iterative use of singular value decomposition. *Journal of Applied Crystallography* **2003**, *36* (1), 86-95. DOI: 10.1107/S0021889802019878.
(16) Coelho, A. A. *TOPAS Academic V5*. Coelho Software, 2012
(17) Pawley, G. S. Unit-cell refinement from powder diffraction scans. *Journal of Applied Crystallography* **1981**, *14* (6), 357-361. DOI: 10.1107/s0021889881009618.
(18) Cavka, J. H.; Jakobsen, S.; Olsbye, U.; Guillou, N.; Lamberti, C.; Bordiga, S.; Lillerud, K. P. A new zirconium inorganic building brick forming metal organic frameworks with exceptional stability. *Journal of the American Chemical Society* **2008**, *130* (42), 13850-13851. DOI: 10.1021/ja8057953.
(19) Øien, S.; Wragg, D.; Reinsch, H.; Svelle, S.; Bordiga, S.; Lamberti, C.; Lillerud, K. P. Detailed structure analysis of atomic positions and defects in zirconium metal-organic frameworks. *Crystal Growth and Design* **2014**, *14* (11), 5370-5372. DOI: 10.1021/cg501386j.
(20) Coelho, A. Whole-profile structure solution from powder diffraction data using simulated annealing. *Journal of Applied Crystallography* **2000**, *33* (3), 899-908. DOI: 10.1107/S002188980000248X.
(21) Young, R. A. *The Rietveld Method*; Oxford University Press, 1981.
(22) Hirshfeld, F. Can X-ray data distinguish bonding effects from vibrational smearing? *Acta Crystallographica Section A* **1976**, *32* (2), 239-244. DOI: doi:10.1107/S0567739476000533.
(23) Thorn, A.; Dittrich, B.; Sheldrick, G. M. Enhanced rigid-bond restraints. *Acta Crystallographica Section A Foundations of Crystallography* **2012**, *68* (4), 448-451. DOI: 10.1107/s0108767312014535.
(24) Bon, V.; Senkovska, I.; Baburin, I. A.; Kaskel, S. Zr- and Hf-Based Metal–Organic Frameworks: Tracking Down the Polymorphism. *Crystal Growth & Design* **2013**, *13* (3), 1231-1237. DOI: 10.1021/cg301691d.
(25) Duerinck, T.; Bueno-Perez, R.; Vermoortele, F.; De Vos, D. E.; Calero, S.; Baron, G. V.; Denayer, J. F. M. Understanding Hydrocarbon Adsorption in the UiO-66 Metal–Organic Framework: Separation of (Un)saturated Linear, Branched, Cyclic Adsorbates, Including Stereoisomers. *The Journal of Physical Chemistry C* **2013**, *117* (24), 12567-12578. DOI: 10.1021/jp402294h.
(26) Batatia I, B. P., Chiang Y, Elena AM, Kovács DP, Riebesell J, Advincula XR, Asta M, Avaylon M, Baldwin WJ, Berger F. A. A foundation model for atomistic materials chemistry. *arXiv preprint,* December 29*,* **2023**. DOI: 10.48550/arXiv.2401.00096.
(27) Hjorth Larsen, A.; Jorgen Mortensen, J.; Blomqvist, J.; Castelli, I. E.; Christensen, R.; Dulak, M.; Friis, J.; Groves, M. N.; Hammer, B.; Hargus, C.; Hermes, E. D.; Jennings, P. C.; Bjerre Jensen, P.; Kermode, J.; Kitchin, J. R.; Leonhard Kolsbjerg, E.; Kubal, J.; Kaasbjerg, K.; Lysgaard, S.; Bergmann Maronsson, J.; Maxson, T.; Olsen, T.; Pastewka, L.; Peterson, A.; Rostgaard, C.; Schiotz, J.; Schutt, O.; Strange, M.; Thygesen, K. S.; Vegge, T.; Vilhelmsen, L.; Walter, M.; Zeng, Z.; Jacobsen, K. W. The atomic simulation environment-a Python library for working with atoms. *J Phys Condens Matter* **2017**, *29* (27), 273002. DOI: 10.1088/1361-648X/aa680e.
(28) Frenkel, D., Smit, B. *Understanding Molecular Simulation,* 2nd ed*;* Academic Press, 2002.
